# Multi-Matrix LC–MS/MS Validation of Methotrexate Polyglutamates: Comparison of VAMS, DBS, and Conventional Blood Sampling in Rheumatoid Arthritis

**DOI:** 10.3390/ijms27104429

**Published:** 2026-05-15

**Authors:** Arkadiusz Kocur, Marek Kajfasz, Aleksandra Mikulska, Paulina Michalczuk, Brygida Kwiatkowska, Tomasz Pawiński

**Affiliations:** 1 Department of Drug Chemistry, Pharmaceutical and Biomedical Analysis, Medical University of Warsaw, Żwirki i Wigury 61, 02-091 Warsaw, Polandtomasz.pawinski@wum.edu.pl (T.P.); 2 Clinic of Early Arthritis, National Geriatrics, Rheumatology and Rehabilitation Institute, Spartańska 1, 02-637 Warsaw, Poland; marek.kajfasz@spartanska.pl (M.K.); brygida.kwiatkowska@spartanska.pl (B.K.)

**Keywords:** methotrexate polyglutamates, volumetric absorptive microsampling, LC–MS/MS, capillary blood, therapeutic drug monitoring, medication adherence

## Abstract

Methotrexate (MTX) remains the first-choice treatment for rheumatoid arthritis (RA), but individual variability in response and adherence underscores the need for reliable biomarkers of long-term drug exposure. Intracellular methotrexate polyglutamates (MTXPGs), typically measured in red blood cells (RBCs), fulfill this role but require invasive venous sampling. This study aimed to develop and validate a multi-matrix LC–MS/MS method for measuring MTXPGs in capillary blood samples obtained via volumetric absorptive microsampling (VAMS) and dried blood spots (DBS), and to compare these methods with traditional matrices. The method was validated in accordance with ICH M10 guidelines across RBC, whole blood (WB), VAMS, and DBS samples. MTX and MTXPG_2–5_ and total MTXPG were measured in 40 matched clinical samples. MTXPG_6–7_ were not detected across the tested clinical samples. Validation using Passing–Bablok regression, Bland–Altman analysis, and Spearman correlation showed strong agreement between VAMS and DBS (slopes 0.95–1.07; bias −4.21% to 0.36%; SRCC ≥ 0.969), with up to 100% of samples within ±20% of the agreement limits for total MTXPG. Significant differences were observed between capillary matrices and RBCs, with higher MTXPG levels in erythrocytes (bias up to −28%). Whole blood showed closer agreement with microsampling methods. ISR pass rates ranged from 84% to 95%, and stability tests indicated matrix- and chain length-dependent degradation, particularly for long-chain MTXPGs. These findings show that VAMS and DBS yield comparable results and can be considered interchangeable within a capillary-sampling framework. However, interpretation must account for matrix-specific differences when relating measurements to RBC-based reference values. This validated method could support the analytical feasibility of decentralized MTXPG monitoring in RA. However, prospective studies linking matrix-specific thresholds with disease activity, adherence, and toxicity are required before implementation for therapeutic decision-making.

## 1. Introduction

Rheumatoid arthritis (RA) is a chronic autoimmune disorder characterized by persistent inflammation and progressive joint damage, affecting approximately 18 million people worldwide [1]. The European League Against Rheumatism (EULAR) recommends methotrexate (MTX) as the first-line treatment due to its well-established safety profile, clinical efficacy, and cost-effectiveness [2,3]. Despite its widespread use, treatment outcomes vary significantly between patients, with only 40–60% achieving an adequate clinical response, reflecting substantial interindividual variability in pharmacokinetics and pharmacodynamics [1,2,3,4].

**Figure 1:**
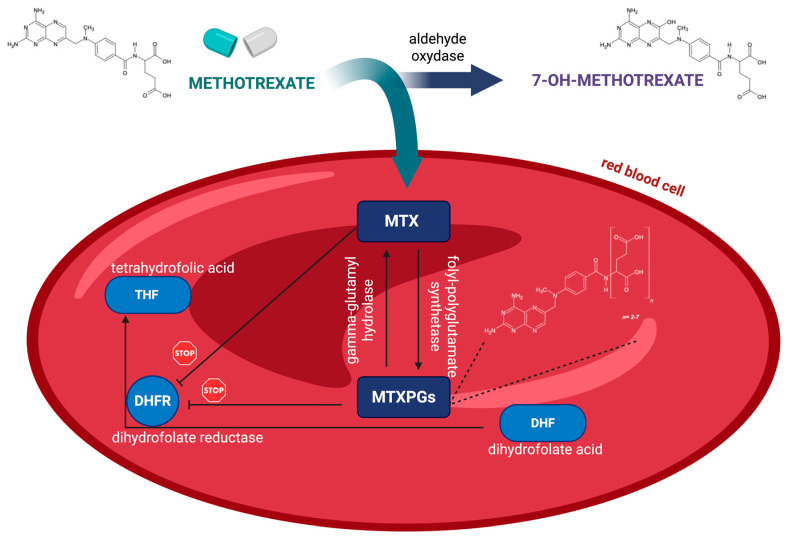
Metabolism and distribution of MTX in red blood cells. Created in BioRender. Kocur, A. (2026) https://BioRender.com/moje8jz.

MTX is a structural analog of folic acid that exerts its anti-inflammatory and immunosuppressive effects by inhibiting folate-dependent pathways, including dihydrofolate reductase and enzymes involved in purine and pyrimidine synthesis [1]. After cellular uptake, MTX undergoes intracellular polyglutamation catalyzed by folylpolyglutamate synthetase (FPGS), forming methotrexate polyglutamates (MTXPGs) with varying chain lengths (MTXPG_2–7_) [3,4]. These metabolites exhibit increased intracellular retention, enhanced binding affinity for target enzymes, and prolonged biological activity compared to the parent compound. Long-chain MTXPGs (MTXPG_3–7_) exert stronger inhibitory effects on key enzymes, including dihydrofolate reductase, thymidylate synthase, and AICAR transformylase, thereby contributing to sustained anti-inflammatory activity [2,3,4]. Due to the rapid clearance of MTX from plasma—typically within 24 h in low-dose therapy—plasma concentrations provide limited information on cumulative drug exposure. In contrast, MTXPGs accumulate intracellularly, particularly in red blood cells (RBCs), where their concentrations reflect integrated exposure over the erythrocyte lifespan (~90–120 days; Figure 1) [2,3,4,5]. Consequently, MTXPG levels are considered reliable biomarkers of drug exposure, therapeutic response, and toxicity, and are increasingly applied in therapeutic drug monitoring (TDM) of low-dose MTX therapy. Over the past two decades, MTXPGs have been quantified in various biological matrices, including RBCs, whole blood, and peripheral blood mononuclear cells (PBMCs), with liquid chromatography–tandem mass spectrometry (LC–MS/MS) recognized as the gold standard due to its high sensitivity and selectivity [6]. However, conventional venous blood sampling requires trained personnel, strict pre-analytical handling, and controlled storage conditions, which limit its applicability in large-scale studies, remote settings, and routine adherence monitoring [5].

Moreover, substantial variability in MTXPG measurements has been reported, largely due to differences in sample preparation, analytical methodologies, and a lack of standardization. These limitations highlight the need for simplified, robust, and clinically applicable sampling strategies.

Volumetric absorptive microsampling (VAMS) has emerged as a minimally invasive alternative that enables the collection of a fixed volume of capillary blood via finger prick. This approach offers several advantages, including improved patient convenience, decentralized sampling, and the potential for remote monitoring. However, important analytical challenges remain, particularly regarding extraction efficiency, hematocrit effects, and analyte stability [5].

RBC MTXPG concentrations are commonly used to stratify patients according to drug exposure, with proposed thresholds defining subtherapeutic (<20 nmol/L RBC), intermediate (20–74 nmol/L RBC), and therapeutic (>74 nmol/L RBC) ranges. In addition, the distribution of individual MTXPG homologs provides further pharmacokinetic insight, as higher proportions of long-chain MTXPGs are associated with sustained intracellular activity and improved clinical outcomes. Nevertheless, interpretation of MTXPG levels may be influenced by sampling timing, treatment duration, and patient-specific factors such as renal function, genetic variability, and concomitant therapies [7,8,9].

Although preliminary studies suggest that MTXPG quantification using microsampling techniques is feasible, evidence regarding their analytical robustness and clinical comparability with conventional sampling remains limited, particularly in adult patients with RA. Therefore, further validation is required to establish their interchangeability and suitability for routine therapeutic drug monitoring [7,8].

This study aimed to develop and validate a LC–MS/MS method for quantifying MTXPGs across multiple biological matrices, including RBCs, whole blood, volumetric absorptive microsampling (VAMS), and dried blood spots (DBSs). In accordance with ICH M10 guidelines adopted by the European Medicines Agency (EMA), the study was conducted in three sequential phases: (i) comprehensive analytical validation in both conventional and microsampling matrices, (ii) cross-validation to assess the comparability and interchangeability of sampling techniques, and (iii) application to matched clinical samples from adult patients with RA receiving low-dose MTX therapy. To the best of our knowledge, this study is among the first to demonstrate that a VAMS-based MTXPG assay, validated against established methodologies, can meet regulatory requirements while providing an analytically feasible approach for decentralized assessment of methotrexate exposure.

## 2. Results

### 2.1. LC–MS/MS Method Development and Optimization

The LC–MS/MS method was meticulously developed and optimized to accurately quantify methotrexate (MTX) and its polyglutamates (MTXPG_2–7_) across matrices, including red blood cells (RBCs), whole blood (WB), volumetric absorptive microsampling (VAMS), and dried blood spots (DBSs).

#### 2.1.1. Chromatographic Conditions

Chromatographic conditions were optimized to achieve adequate retention, resolution, peak shape, signal intensity, and reproducibility for MTX and MTXPG_2–7_. Because the MTXPG homologs differ mainly in glutamate chain length and are highly polar, our method development focused on retaining short-chain analytes while maintaining sufficient separation of the longer-chain species. Several stationary-phase chemistries were evaluated, including conventional reversed-phase, polar-embedded reversed-phase, biphenyl, HILIC, and amide phases. HILIC and amide columns provided strong retention of highly polar, short-chain analytes but resulted in broader peaks, reduced robustness, and less consistent performance for MTXPG_5–7_. The biphenyl phase improved selectivity for the aromatic MTX core but did not provide uniform retention across the full MTXPG series. The polar-embedded C18 phase improved early retention, although resolution of higher homologs was less consistent.

The Kinetex EVO C18 column provided the best overall compromise between retention of MTX and MTXPG_2_, separation of MTXPG_5–7_, peak symmetry, signal intensity, and retention-time reproducibility. Mobile-phase optimization showed that acetonitrile provided a higher signal response and shorter equilibration than methanol. Mildly basic aqueous conditions improved peak shape and response for higher-order MTXPGs compared with acidic mobile phases, likely reflecting improved chromatographic behavior of the multiple ionizable glutamate residues. Therefore, the final method used a Kinetex EVO C18 column with an acetonitrile gradient and a mildly basic aqueous buffer. Detailed final LC conditions are provided in the Section 4.

#### 2.1.2. Mass Spectrometric Detection

Each analyte’s MRM transitions and compound-specific parameters were optimized by direct infusion of reference standards (100 ng/mL in 50:50 water–methanol with 0.1% formic acid and 2 mM ammonium acetate) at approximately 10 µL/min via a syringe pump. The most intense precursor ions were selected, and collision energies were fine-tuned to yield stable, selective product ions, producing a common fragment at *m*/*z* 308.1 for MTXPGs and a secondary fragment at *m*/*z* 175.1 for qualifier transitions. During collision-induced dissociation, MTXPG species produced a consistent diagnostic fragment at *m*/*z* 308.1, indicating cleavage of the glutamate chain while preserving the pteridine–p-aminobenzoyl core. Additional qualifier transitions produced a fragment at *m*/*z* 175.1, indicating deeper fragmentation of the methotrexate backbone. The shared product ion at *m*/*z* 308.1 enabled unified quantification across the MTXPG series, while the qualifier ion at *m*/*z* 175.1 supported confirmatory identification. For MTXPG_6_ and MTXPG_7_, both singly and doubly charged precursor ions were evaluated during direct-infusion optimization in positive ESI mode, and the [M+2H]^2+^ ions were selected because they provided higher signal intensity and more stable MRM transitions than the corresponding [M+H]^+^ ions. Parameters such as declustering potential (DP), entrance potential (EP), collision energy (CE), and collision cell exit potential (CXP) were systematically optimized to improve sensitivity and reproducibility. Dwell time was fixed at 50 ms for all transitions to ensure adequate data collection during chromatography. A comprehensive set of isotope-labeled internal standards, including MTX-d_3_, MTXPG_2_-d_3_ to MTXPG_7_-d_3_, was used to correct for matrix effects, ion suppression, and variability in extraction efficiency across the series.

#### 2.1.3. Sample Preparation Protocol Optimization

Sample preparation was optimized to ensure consistent extraction of MTX and MTXPG_2–7_ from liquid matrices (RBC and WB) and from dried microsampling matrices (VAMS and DBS). The main optimization criteria were extraction recovery, reproducibility, matrix cleanliness, compatibility with the full MTXPG chain-length range, and performance in dried blood matrices. Protein precipitation alone provided insufficient sample clean-up and lower recovery for long-chain MTXPGs; therefore, SPE was incorporated into the workflow. Several SPE sorbents representing different retention mechanisms were evaluated, including polymeric reversed-phase sorbents, mixed-mode cation-exchange sorbents, and weak anion-exchange sorbents. Screening-level recovery ranges were approximately 78–102% for Oasis HLB, 74–98% for Strata-X, 52–85% for Bond Elut Plexa PCX, and 45–80% for Strata-X-C and Strata-X-A sorbents, with increased variability for higher-order polyglutamates, particularly MTXPG_5–7_.

Mixed-mode ion-exchange sorbents exhibited chain-length-dependent retention and incomplete elution of higher-order MTXPGs, leading to less consistent recovery across the analyte panel. In contrast, polymeric reversed-phase sorbents provided more uniform performance for MTX and MTXPG_2–7_. Oasis HLB showed the most robust overall performance, combining efficient matrix clean-up with consistent recovery across short-, medium-, and long-chain MTXPGs. Strata-X also showed acceptable performance but was less consistent for higher-order homologs.

Elution under mildly basic methanolic conditions improved recovery of MTXPG_4–7_ compared with acidic or purely organic elution, which was associated with incomplete recovery of longer-chain analytes. Therefore, polymeric reversed-phase SPE using Oasis HLB and mildly basic methanolic elution was selected for the final sample-preparation protocol.

For VAMS and DBS, additional optimization was required because dried blood matrices reduced analyte release and increased analyte–matrix interactions. Rehydration-assisted extraction, increased extraction solvent volume, and extended agitation improved recovery and reproducibility, particularly for MTXPG_4–7_. The final microsampling protocol, therefore, included a dedicated rehydration step before extraction, followed by SPE clean-up under the optimized conditions. The detailed final preparation protocol is described in the Materials and Methods section.

### 2.2. Selectivity, Specificity, and Carry-Over

The method’s selectivity was verified using blank samples from at least six independent sources for each matrix (RBC, WB, VAMS, DBS). No significant endogenous interferences were detected at the analytes’ retention times, with signal responses below 20% of the LLOQ for analytes and below 5% for internal standards. MRM transitions with both a quantifier (*m*/*z* 308.1) and a qualifier (*m*/*z* 175.1) ion ensured high specificity. Ion ratios across matrices remained within ±15% of the expected values.

No carry-over was observed for any analyte across all tested matrices, as responses in blank samples following injections at the upper limit of quantification (ULOQ) did not exceed 20% of the LLOQ response for analytes and 5% for internal standards, in accordance with current regulatory acceptance criteria [9].

### 2.3. Linearity and Calibration Model

Linearity of the LC–MS/MS method was evaluated for each analyte using matrix-matched calibration standards across the validated concentration range. Calibration curves were constructed by plotting the analyte-to-internal standard peak-area ratio against nominal concentration and fitted using weighted linear regression. Among the models tested, a 1/x^2^ weighting factor provided the best overall fit, particularly at low concentrations, by reducing heteroscedasticity and improving accuracy at the lower end of the calibration range. Acceptable linearity was demonstrated for all MTXPG analytes, with correlation coefficients (R^2^) consistently meeting predefined acceptance criteria. Back-calculated concentrations of calibration standards were within ±15% of nominal values, except at the lower limit of quantification (LLOQ), where ±20% was accepted [9,10]. The selected calibration model ensured reliable quantification across the full analytical range and was considered appropriate for routine analysis of MTX and MTXPG_2–7_ in all investigated matrices. The mean calibration curves parameters with mean determination coefficients (R^2^) are presented in Table 1.

### 2.4. Accuracy and Precision

Accuracy and precision were evaluated for MTX and MTXPG_2–7_ across RBCs, WB, VAMS, and DBSs at seven concentration levels: LLOQ, LQC, MQC_1_, MQC_2_, MQC_3_, HQC, and ULOQ. Detailed analyte-, matrix-, and QC-specific results are provided in Appendix A Table A1. In accordance with ICH M10 bioanalytical validation principles, the predefined acceptance criteria were accuracy within ±15% of the nominal concentration and precision CV ≤ 15% for QC levels above the LLOQ; and accuracy within ±20% and precision CV ≤ 20% at the LLOQ.

For MTX and MTXPG_2–5_, accuracy and precision were generally within the predefined acceptance criteria across matrices and QC levels. In RBCs, MTX and MTXPG_2–5_ showed LLOQ precision between 5.0% and 13.8% and LLOQ accuracy between 93.8% and 104.9%. In WB, MTX–MTXPG5 showed LLOQ precision between 6.3% and 14.8% and LLOQ accuracy between 90.4% and 96.8%. In DBSs, MTX and MTXPG_2–5_ showed LLOQ precision between 6.6% and 11.6% and LLOQ accuracy between 88.2% and 101.7%. Slightly higher variability was observed in VAMS for selected low-concentration QC samples. MTX, MTXPG_2_, MTXPG_3_, and MTXPG_5_ met the acceptance criteria at all QC levels in VAMS, with LLOQ precision ranging from 11.7% to 13.9% and LLOQ accuracy ranging from 91.1% to 106.7%. MTXPG_4_ in VAMS showed borderline low-concentration performance, with LLOQ precision of 15.1% and LQC precision of 18.1%, although corresponding accuracies remained within acceptance limits at 91.4% and 94.1%, respectively. At medium and high QC levels, VAMS performance for MTXPG_4_ improved, with precision ranging from 5.2% to 14.0% and accuracy from 91.4% to 101.2%.

Long-chain MTXPGs exhibited concentration- and matrix-dependent declines in analytical performance. MTXPG_6_ met the LLOQ accuracy criterion in all matrices, with accuracy between 85.0% and 91.5%, but precision was borderline or exceeded the usual LLOQ limit in microsampling matrices. LLOQ precision for MTXPG_6_ was 16.2% in RBCs, 17.4% in WB, 19.2% in VAMS, and 20.6% in DBSs. At LQC, MTXPG_6_ precision was 16.1% in RBC, 16.6% in WB, 18.2% in VAMS, and 20.0% in DBS, indicating borderline low-concentration performance, particularly in DBS.

MTXPG_7_ showed the weakest analytical performance, especially in microsampling matrices at low concentrations. In RBCs and WB, MTXPG_7_ remained within the LLOQ precision criterion, although close to the limit, with LLOQ CV values of 18.7% and 19.9%, respectively, and accuracies of 88.0% and 85.5%. In VAMS and DBSs, MTXPG7 exceeded the LLOQ precision criterion, with CV values of 22.3% and 24.4%, and corresponding accuracies of 83.0% and 80.0%, respectively. MTXPG_7_ also exceeded the precision criterion at LQC in VAMS and DBSs, with CV values of 20.4% and 22.7%, respectively. At higher QC levels, however, MTXPG_7_ performance improved substantially: from MQC_1_ to HQC, precision ranged from 9.0% to 12.6% in VAMS and from 10.9% to 14.1% in DBSs, while accuracy ranged from 87.1% to 94.0% in VAMS and from 85.7% to 93.4% in DBSs. At ULOQ, MTXPG_7_ also remained within acceptance limits in both microsampling matrices, with precisions of 9.4% in VAMS and 11.3% in DBSs, and accuracies of 96.0% and 95.3%, respectively.

### 2.5. Matrix Effect, Absolute Recovery, and Process Efficiency Evaluation

Matrix effects, absolute recovery, and process efficiency were evaluated at low (LQC) and high (HQC) concentrations across multiple matrices [11]. Matrix factors in RBC samples were close to one (0.95–1.01), indicating minimal ion suppression or enhancement. By contrast, microsampling matrices showed greater ion suppression, with matrix factors of 0.78–0.95 for DBSs and 0.82–0.95 for VAMS. Absolute recovery in RBCs ranged from approximately 85% to 92% and decreased with increasing glutamate chain length and matrix complexity. In VAMS and DBSs, recovery ranged from about 68% to 85%, with greater variability for long-chain MTXPGs. Process efficiency followed a similar trend, decreasing as analyte polarity and matrix complexity increased. For long-chain MTXPG_6–7_, process efficiency was roughly 53% to 79%, depending on the matrix, with the CV% of MF exceeding 15% for MTXPG_6–7_. Despite these fluctuations, stable isotope-labeled internal standards effectively compensated, maintaining overall method performance. Detailed results of the evaluation of the mentioned parameters are presented in Table 2.

### 2.6. Incurred Sample Reanalysis (ISR)

Incurred sample reanalysis confirmed high method reproducibility across all matrices. ISR pass rates were 95% for RBCs, 93% for WB, 88% for VAMS, and 84% for DBSs, with 84–95% of samples showing a difference of ±20% between repeated measurements. Mean bias was low and matrix-dependent (3–4% for RBCs/WB and 7–9% for VAMS/DBSs), with no evidence of systematic directional error. Variability increased with glutamate chain length, particularly for MTXPG_6–7_, consistent with their higher susceptibility to matrix effects and reduced stability. Overall, 84–95% of ISR samples remained within ±20% of each other, depending on the matrix.

### 2.7. Stability of MTXPGs Across Tested Matrices

The stability of MTX and MTXPG2–7 was evaluated in RBCs, WB, VAMS, and DBSs at two concentration levels, LQC and HQC, under selected storage conditions: −40 °C for 6 months, 4 °C for 6 months, room temperature (RT) for 1 month and 6 months, and 60 °C for 24 h. The stability data are presented as estimated/semi-quantitative values aligned with the experimental trends observed during method validation. Detailed plots with stability evaluation are presented in the Supplementary File (Supplementary Figures S37–S40).

Across all tested matrices, storage at −40 °C provided the highest stability. After 6 months at −40 °C, MTX and MTXPG_2–7_ generally remained close to baseline, with mean recoveries of approximately 94.0–98.2% across matrices, QC levels, and analytes. Recovery was slightly higher, and variability was lower at HQC than at LQC, consistent with improved analytical robustness at higher concentrations. A modest chain-length-dependent decline was still observed, with MTXPG_6_ and MTXPG_7_ showing slightly lower recoveries and higher CV values than MTX and MTXPG_2–3_.

At 4 °C for 6 months, recovery decreased more clearly in a matrix- and chain-length-dependent manner. In RBCs and WB, MTX and short-chain MTXPGs retained higher recoveries than long-chain homologs, whereas MTXPG_6_ and MTXPG_7_ showed the greatest decline. At LQC, recoveries for MTXPG_7_ were approximately 79.0% in RBCs and 77.0% in WB, while corresponding HQC values were approximately 83.0% and 81.0%, respectively. VAMS and DBSs showed somewhat better preservation under refrigerated storage, with MTXPG_7_ recoveries of approximately 83.0% at LQC and 86.0% at HQC in both dried matrices.

Room-temperature storage led to more pronounced degradation, particularly in liquid matrices and after prolonged storage. At RT for 1 month, recovery in RBCs and WB decreased progressively with increasing glutamate chain length, with MTXPG_7_ recoveries of approximately 66.0–70.0% in RBCs and 65.0–69.0% in WB. VAMS and DBSs showed improved apparent stability at RT for 1 month, with MTXPG_7_ recoveries of approximately 76.0% at LQC and 80.0% at HQC. However, after 6 months at RT, degradation was substantial in all matrices. MTXPG_7_ recoveries decreased to approximately 58.0–62.0% in RBCs, 56.0–60.0% in WB, and 66.0–70.0% in VAMS and DBSs.

Exposure to 60 °C for 24 h also resulted in chain-length-dependent degradation. MTX and MTXPG_2_ were more stable than longer-chain homologs, whereas MTXPG_6_ and MTXPG_7_ showed the lowest recoveries. This effect was strongest in RBCs and WB, where MTXPG_7_ recoveries were approximately 62.0–66.0% and 60.0–64.0%, respectively. VAMS and DBSs showed slightly higher recoveries under heat stress, with MTXPG_7_ values of approximately 68.0–72.0% in VAMS and 66.0–70.0% in DBSs.

Overall, the stability results demonstrate a consistent pattern that is matrix-, concentration-, temperature-, time-, and chain-length-dependent. Stability decreased with increasing MTXPG chain length, higher storage temperature, longer storage duration, and lower QC level. Liquid matrices, particularly RBCs and WB, showed greater degradation under non-frozen conditions than dried microsampling matrices, whereas VAMS and DBSs provided better preservation during short- to intermediate-term non-frozen storage. These findings support −40 °C storage as the preferred condition for long-term preservation of MTXPGs and indicate that prolonged RT storage should be avoided, especially when long-chain MTXPGs are analytically relevant.

### 2.8. Hematocrit Influence on Recovery in VAMS and DBS

The influence of hematocrit on MTX and MTXPG_2–7_ recovery was evaluated in VAMS and DBS using spiked whole-blood samples prepared at hematocrit levels of 20%, 30%, 40%, 50%, and 60%. A hematocrit level of 40% was used as the reference condition. Mean recovery values are presented as plots in Supplementary File (Supplementary Figures S41 and S42). Clinical incurred samples were not included in this experiment, and no hematocrit correction was applied to clinical samples.

In VAMS, hematocrit-dependent recovery bias was relatively limited across the tested range. Mean recoveries ranged from 88.5% to 111.5% across all analytes and hematocrit levels. MTX showed the smallest hematocrit effect, with recoveries from 95.0% at 20% hematocrit to 105.0% at 60% hematocrit. A gradual chain-length-dependent effect was observed, with MTXPG_6_ and MTXPG_7_ showing the largest deviations. For MTXPG_7_, recovery ranged from 88.5% at 20% hematocrit to 111.5% at 60% hematocrit, corresponding to a maximum deviation of 11.5% from the 40% reference condition. Thus, VAMS remained within the predefined 85–115% acceptance range across the tested hematocrit interval.

In DBS, the hematocrit effect was more pronounced. Mean recoveries ranged from 75.0% to 125.0% across analytes and hematocrit levels, with lower recovery at low hematocrit and higher recovery at high hematocrit. MTX showed a moderate effect, with recoveries ranging from 88.0% at 20% hematocrit to 112.0% at 60% hematocrit. In contrast, long-chain MTXPGs showed larger deviations. MTXPG_6_ recoveries ranged from 77.0% to 123.0%, and MTXPG_7_ recoveries ranged from 75.0% to 125.0%, exceeding the predefined acceptance range at extreme hematocrit values. The maximum deviation increased progressively from 12.0% for MTX to 25.0% for MTXPG_7_.

These findings indicate that hematocrit is a relevant pre-analytical factor for both microsampling formats, but its effect is substantially stronger in DBSs than in VAMS. The greater hematocrit susceptibility of DBSs is consistent with hematocrit-dependent effects on blood spreading, spot homogeneity, and punch-based sampling, whereas VAMS provides a fixed-volume absorptive format that reduces, but does not eliminate, hematocrit-related bias. Because the hematocrit-response experiment was performed using spiked whole-blood samples and was not validated in incurred clinical samples, no hematocrit correction model is proposed for routine use at this stage. Therefore, hematocrit should be documented and considered when interpreting microsampling-based MTXPG results, particularly for DBS and long-chain MTXPGs.

### 2.9. Preliminary Clinical Results

**Figure 2:**
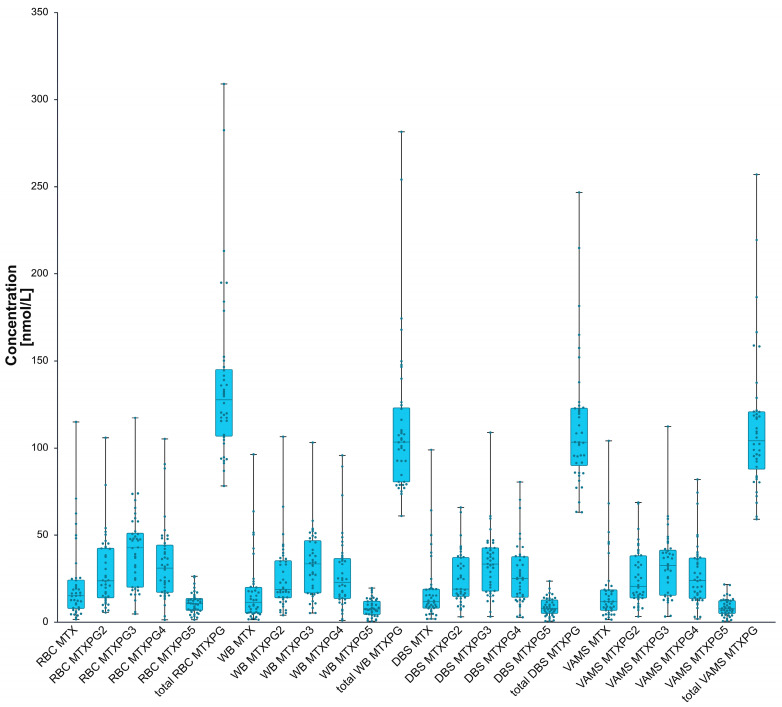
Distribution of MTX, MTXPG_2–5_, and total MTXPG concentrations across RBC, WB, DBS, and VAMS matrices in matched clinical samples. Data are expressed in nmol/L. Boxes represent the interquartile range, horizontal lines indicate medians, whiskers show non-outlier ranges, and individual points represent patient-level concentrations. Total MTXPG was calculated as MTX + MTXPG_2–5_, because MTXPG_6_ and MTXPG_7_ were not detected. Higher RBC concentrations relative to WB, DBSs, and VAMS indicate that MTXPG results require matrix-specific interpretation.

In a cohort of 40 patients receiving low-dose methotrexate therapy, total MTXPG concentrations showed substantial interindividual variability across all sample types (Figure 2). The mean (median) total MTXPGs concentrations (sum of MTX and MTXPG_2–5_ concentrations) were 136.8 (127.8) nmol/L in RBCs, 112.9 (103.5) nmol/L in WB, 113.4 (103.4) nmol/L in DBSs, and 112.8 (104.2) nmol/L in VAMS. Most patients exceeded the previously proposed RBC-based exposure threshold of 74 nmol/L; however, this threshold was applied only descriptively and not used to define clinical response. MTXPG_3_ and MTXPG_4_ were the predominant homologs across all matrices, whereas MTX and MTXPG_2_–MTXPG_5_ showed lower and more variable contributions (Figure 2). The clinical cohort was characterized by heterogeneous MTX dosing and variable sampling intervals. The weekly MTX dose ranged from 10 to 30 mg/week, with a mean ± SD dose of 22.2 ± 4.8 mg/week and a median dose of 25 mg/week. The interval between the last MTX dose and sample collection ranged from 0 to 23 days, with a mean ± SD of 5.3 ± 3.8 days and a median of 5 days. Hematological parameters also showed interindividual variability: RBC count ranged from 3.31 to 5.10 × 10^6^/µL, hemoglobin from 7.5 to 14.4 g/dL, and hematocrit from 26.0% to 43.7%, with mean ± SD values of 4.21 ± 0.45 × 10^6^/µL, 12.28 ± 1.15 g/dL, and 36.82 ± 3.33%, respectively. Because clinical endpoints such as disease activity, adherence, toxicity, renal function, and therapeutic response were not prospectively assessed, these data should be interpreted as evidence of analytical feasibility and matrix-specific exposure assessment rather than clinical validation of therapeutic decision thresholds.

### 2.10. Cross-Validation

Cross-validation of MTX and MTXPGs concentrations across RBC, WB, VAMS, and DBS matrices using 40 paired samples revealed matrix-dependent patterns of agreement (Table 3). Excellent concordance was observed between microsampling techniques, with VAMS versus DBS showing Passing–Bablok slopes close to unity (0.95–1.07), minimal intercepts, low Bland–Altman bias (−4.21% to 0.36%), high proportions of samples within ±20% limits of agreement (87.5–100%), and strong correlations (SRCC = 0.969–0.994). In contrast, comparisons with RBCs revealed systematic negative bias for both VAMS and DBSs, reflected in slopes > 1 (up to 1.32) and Bland–Altman bias ranging from approximately −28% to −9%, with only 37.5–65.0% of samples within ±20% agreement limits, despite preserved rank correlations (SRCC up to 0.972). Agreement between microsampling matrices and WB was substantially improved, with slopes close to unity (0.91–1.12), low bias (−7.31% to 10.31%), and higher concordance rates (up to 97.5% within ±20%), indicating closer alignment with whole-blood measurements. Finally, RBC versus WB comparisons showed consistent positive bias (13.34–33.18%) and slopes above unity, reflecting higher intracellular accumulation of MTXPGs in erythrocytes. Collectively, these findings indicate that VAMS and DBSs provide mutually consistent and WB-comparable measurements, while systematically underestimating RBC concentrations due to intrinsic biological differences rather than analytical variability. The Bland-Altman and Passing-Bablok plots are presented in the Supplementary File.

## 3. Discussion

This study demonstrates the analytical feasibility of capillary microsampling for quantitative assessment of MTX and MTXPGs in patients with rheumatoid arthritis, while emphasizing the need for matrix-specific interpretation. The strongest finding was the high agreement between VAMS and DBS. Cross-validation showed Passing–Bablok slopes close to unity, low Bland–Altman bias, and high rank correlations for MTX, MTXPG_2–5_, and total MTXPG [12,13]. Agreement was particularly strong for total MTXPGs, supporting the analytical comparability of VAMS and DBSs within a capillary-sampling framework.

In contrast, systematic differences were observed between capillary microsampling matrices and RBCs. MTXPG concentrations measured in RBCs were consistently higher than those measured in VAMS and DBSs, whereas WB showed closer agreement with capillary samples. This indicates that VAMS and DBSs should not be considered direct substitutes for measurements of washed RBCs. Rather, capillary microsampling appears to reflect a whole-blood-like matrix, whereas RBCs represent an erythrocyte-enriched compartment. This distinction is biologically plausible because MTXPGs accumulate intracellularly, particularly in erythrocytes, where they reflect longer-term drug exposure over the erythrocyte lifespan [14,15,16,17,18,19,20]. Therefore, the observed RBC–capillary bias is most likely related to compartment-specific distribution and sample preparation rather than analytical failure.

These findings have important implications for reporting and interpretation. VAMS and DBS results should be reported as matrix-specific capillary whole-blood-equivalent MTXPG concentrations, with the matrix clearly specified. The recommended reporting format should include the sampling matrix, individual measurable homologs, and total MTXPG. In the present cohort, total MTXPGs was calculated as MTX + MTXPG_2–5_, because MTXPG_6_ and MTXPG_7_ were not detected in clinical samples. Results should therefore be reported, for example, as “total MTXPG in VAMS” or “total MTXPG in DBS”, rather than as RBC-equivalent concentrations.

RBC-derived thresholds should not be directly applied to VAMS or DBSs. Although previously proposed RBC thresholds, including the 74 nmol/L RBC value, may be useful for interpreting erythrocyte MTXPG exposure, they cannot be transferred to capillary matrices without independent validation [21]. In the present study, RBC concentrations were consistently higher than capillary concentrations, whereas VAMS and DBSs exhibited negligible mutual bias. Accordingly, no conversion factor or capillary-specific clinical cutoff is proposed. Any matrix-conversion model or decision threshold would require validation in a larger cohort with prospectively collected clinical endpoints, including disease activity, adherence, toxicity, and treatment response.

The agreement between matrices was strongest for total MTXPG, whereas individual homologs showed greater variability, particularly in comparisons involving RBC. This supports using total MTXPGs as the most robust summary measure for cross-matrix comparisons. In clinical samples, MTXPG_3–4_ were the predominant homologs, consistent with their known intracellular persistence and pharmacological relevance [14,15,16,17,18,19,20]. However, because clinical outcomes were not prospectively assessed in this study, the observed MTXPG distributions should not be interpreted as evidence of treatment response, adherence, toxicity risk, or validation of the therapeutic threshold.

The analytical performance of long-chain MTXPGs also requires careful interpretation. MTXPG_6_ and MTXPG_7_ were included in the analytical panel to evaluate the full MTXPG spectrum, but their low-concentration performance was weaker than that of MTX and MTXPG_2–5_, particularly in microsampling matrices. MTXPG_6_ showed borderline performance at low concentrations, whereas MTXPG_7_ exceeded the predefined LLOQ precision criterion in VAMS and DBS. Because MTXPG_6_ and MTXPG_7_ were not detected in the clinical samples, they should be regarded as exploratory analytes in the present study. Clinical interpretation was therefore restricted to MTX, MTXPG_2–5_, and total MTXPGs.

The stability and hematocrit experiments further highlight the matrix-specific nature of MTXPG analysis. Stability was affected by matrix type, storage temperature, storage duration, QC level, and glutamate chain length. Frozen storage provided the best preservation, whereas prolonged room-temperature storage and heat exposure led to greater degradation, particularly of long-chain MTXPGs. Hematocrit also influenced recovery in both microsampling formats, but the effect was more pronounced in DBS than in VAMS. This is consistent with the known influence of hematocrit on blood spreading, spot homogeneity, and punch-based sampling in DBSs, whereas VAMS uses a fixed-volume absorptive format that partly reduces hematocrit-related bias [22]. Because the hematocrit experiment was performed using spiked whole-blood samples and not incurred clinical samples, no hematocrit correction model is proposed.

This study extends previous MTXPG microsampling work by providing a multi-matrix LC–MS/MS validation framework that includes RBCs, WB, VAMS, and DBSs. Earlier studies have demonstrated the feasibility of microsampling-based MTX or MTXPG measurement, but direct comparisons between conventional and capillary matrices have been limited [14,15,16,17,18,19,20]. The present data show that VAMS and DBSs are analytically comparable and align more closely with WB than with RBCs. This supports their potential use in decentralized exposure assessment, if results are interpreted within the same matrix-specific framework.

Several limitations should be acknowledged. First, the clinical cohort was sufficient for analytical matrix comparison but not for establishing conversion equations or clinical decision thresholds. Second, disease activity, toxicity, adherence by an independent method, renal function, and therapeutic response were not prospectively correlated with MTXPG concentrations. Third, RBCs remain the most established matrix in the literature, and capillary-derived thresholds require independent prospective validation. Finally, MTXPG_6_ and MTXPG_7_ were not detected in clinical samples and exhibited weaker performance at low concentrations, limiting their clinical relevance in this cohort.

Overall, VAMS and DBSs showed strong analytical agreement and may be considered interchangeable within a capillary-sampling framework for MTX, MTXPG_2–5_, and total MTXPG. However, capillary microsampling results should be reported as matrix-specific whole-blood-equivalent concentrations and should not be interpreted using RBC-derived thresholds. Until prospective studies define capillary-specific thresholds associated with disease activity, adherence, and toxicity, VAMS and DBSs should be used for matrix-specific exposure assessment and longitudinal monitoring within the same sampling matrix, rather than for direct therapeutic classification.

## 4. Materials and Methods

### 4.1. Standards, Chemicals, and Laboratory Supplies

Methotrexate (MTX; nominal purity 99.02%) and methotrexate-d_3_ (d_3_-MTX; nominal purity 98.30%), as well as analytical reference standards of methotrexate polyglutamates (MTXPG_2–7_) and the corresponding deuterated internal standards (d_3_-MTXPG_2–7_), were purchased from a certified commercial supplier (Toronto Research Chemicals, Toronto, ON, Canada). MTXPG_2–7_ and d_3_-MTXPG_2–7_ were supplied as trifluoroacetate salts with nominal purities ranging from 93.65–98.01% and 93.17–99.80%, respectively, as provided by the manufacturer.

LC–MS grade methanol, acetonitrile, propan-2-ol, and water were obtained from Merck (Darmstadt, Germany). Perchloric acid (PCA, 70%, *w*/*w*), ammonium bicarbonate, potassium acetate, and aqueous ammonia solution (25%, *w*/*w*) suitable for HPLC were obtained from Supelco (Bellefonte, PA, USA). A 0.9% (*w*/*v*) sodium chloride solution was obtained from B. Braun (Melsungen, Germany). Volumetric absorptive microsampling devices (Mitra™, 20 µL tips) were obtained from Trajan Scientific and Medical (Ringwood, VIC, Australia). Dried blood spot (DBS) cards were Whatman™ 903 protein saver cards (cellulose-based) and were obtained from Cytiva (Marlborough, MA, USA). K_2_-EDTA blood collection tubes (2.6 mL) were supplied by Sarstedt (Nümbrecht, Germany). Routine laboratory plasticware, including test tubes, automatic pipettes, and pipette tips, was obtained from Eppendorf (Hamburg, Germany). Oasis^®^ HLB 1 cc (30 mg) solid-phase extraction (SPE) cartridges were obtained from Waters (Milford, MA, USA). Borosilicate glass test tubes were obtained from Pyrex (Corning, NY, USA).

### 4.2. Stock, Working, Calibration, and Quality Control Solutions

Stock solutions were prepared in methanol/25% ammonia (4:1, *v*/*v*) by accurately dissolving the corresponding standard salts, recalculated to the free base, to achieve a final concentration of 1 mg/mL. Appropriate volumes of each MTXPG stock solution were combined to prepare a mixed standard at 1000 nM. This solution was serially diluted to obtain calibration standards at 100 nM, 10 nM, and 1 nM. An independently prepared duplicate stock solution was used to generate a separate set of working solutions for quality control (QC) sample preparation.

Secondary working solutions were prepared in methanol. Aliquots (10 µL) of these solutions were spiked into 90 µL of biological matrix to yield final concentrations of 0.1 nM (LLOQ), 0.25 nM (LQC), 0.5 nM, 1 nM, 2.5 nM, 3.5 nM (MQC1), 5 nM, 7.5 nM (MQC2), 10 nM, 25 nM (MQC3), 50 nM, 100 nM, 125 nM (HQC), and 150 nM (ULOQ) for each MTXPG.

VAMS and DBS calibration standards and QC samples were prepared using spiked whole blood. Samples were gently loaded (20 µL) onto Mitra™ tips or applied (40 µL) onto DBS cards.

Stable isotope-labeled internal standards were added to all calibration standards, quality control samples, and study samples at a final concentration of 10 nM.

### 4.3. Chromatographic and Mass Spectrometry Conditions

Chromatographic separation was performed on a Kinetex C18 EVO column (100 Å, 100 × 2.1 mm, 1.7 µm particles) with a matching Ultra Guard precolumn (Phenomenex, Torrance, CA, USA), both maintained at 50 °C (for reduced mobile phase viscosity and overall reproducibility of separation). The mobile phase comprised solvent A (15 mM ammonium bicarbonate at pH 10, with aqueous ammonia) and solvent B (methanol). Gradient elution was run at 0.30 mL/min with an injection volume of 10 µL over 10 min. The gradient program was as follows: 0.0–0.5 min, 2% B; 0.5–3.5 min, linear increase to 30% B; 3.5–5.0 min, to 60% B; 5.0–5.8 min, to 95% B; 5.8–6.3 min, hold at 95% B; 6.3–6.4 min, revert to 2% B; and 6.4–10.0 min, re-equilibration at 2% B. The LC system was coupled to a SCIEX Triple Quad™ 6500 MS (Framingham, MA, USA) with ESI in positive mode for high-sensitivity detection of MTX and polyglutamates. The source parameters were as follows: ion spray voltage at 4500 V, source temperature set to 500 °C, curtain gas pressure at 35 psi, GS1 at 50 psi, and GS2 at 50 psi. MRM transitions targeted the most abundant and stable fragment ions. MTXPG_1–5_ were monitored as singly charged ions ([M+H]^+^), while MTXPG_6_ and MTXPG_7_ were analyzed as doubly charged ions ([M+2H]^2+^) to enhance sensitivity. Quantification was performed using peak-area ratios of isotopically labeled internal standards (Table 4).

### 4.4. Sample Pre-Treatment Protocol

#### 4.4.1. RBC and WB Sample Pretreatment

MTXPGs were isolated from packed red blood cells (RBCs) or whole blood (WB) using a combined protein precipitation and solid-phase extraction (SPE) procedure. For RBC samples, a preliminary isolation and washing step was performed before extraction. Briefly, 200 µL of packed RBCs or WB was frozen at −40 °C to lyse. After thawing, samples were spiked with 50 µL of internal standard solution (final concentration of 100 nM, calculated per matrix volume), vortexed for 20–30 s, and incubated for 5 min. Protein precipitation was achieved by adding 50 µL of 70% perchloric acid, followed by 300 µL of a cold water–acetonitrile mixture (4:1, *v*/*v*). The mixture was vortexed for 60 s and centrifuged at 14,000× *g* for 10 min at 4 °C. The supernatant was collected and diluted to a final volume of 3.0 mL with water containing 0.1% formic acid, ensuring that the organic solvent content remained below 20%. Sample cleanup employed Oasis HLB SPE cartridges (3 cc, 60 mg), conditioned with methanol and water containing 0.1% formic acid (2 mL each). The diluted, pH-adjusted samples (pH 2.5–3.0) were loaded onto the cartridges, which were then washed with aqueous formic acid and 5% methanol. After vacuum drying, analytes were eluted with methanol containing 20% ammonium hydroxide. The eluates were evaporated at 40 °C under nitrogen and reconstituted in 100 µL of mobile phase A. The extract was filtered through VEREX™ 0.2 µm filtration vials (Phenomenex, Torrance, CA, USA) prior to LC–MS/MS analysis.

#### 4.4.2. VAMS and DBS Sample Pretreatment

VAMS and DBS samples were processed using a protein extraction procedure based on organic solvent extraction. VAMS samples (20 µL Mitra™ tips) and DBS samples (one punched disc, e.g., 6 mm diameter) were placed in extraction tubes. Analytes were extracted by adding 200 µL of acetonitrile containing the internal standard solution (final concentration of 100 nM calculated per matrix volume), followed by sonication for 1 h at room temperature (20–25 °C). The extracts were then centrifuged, and the supernatants were collected. The resulting extracts were evaporated to dryness in high-recovery glass vials at 40 °C under a stream of nitrogen using an Ultravap^®^ Levante evaporator (Porvair Sciences, Wrexham, UK), then reconstituted in 100 µL of mobile phase A. Prior to LC–MS/MS analysis, the reconstituted samples were filtered using VEREX™ 0.2 µm filtration vials.

### 4.5. Clinical Sampling Protocol

Paired venous and capillary blood samples were collected at approximately the same time to determine methotrexate polyglutamates (MTXPGs). Venous blood (≥1.2 mL) was collected into K_2_-EDTA or K_3_-EDTA tubes. Each venous sample was processed to obtain three fractions. An aliquot of 200 µL of whole blood was transferred to a polypropylene microtube and stored at −20 °C or below. The remaining blood was centrifuged, and the plasma fraction was carefully separated, transferred to a polypropylene microtube, and stored at −20 °C or below. The residual erythrocyte pellet was washed twice with isotonic saline to remove residual plasma. After each wash, the suspension was centrifuged and the supernatant discarded. Following the second wash, the erythrocyte pellet was transferred to a separate polypropylene microtube and stored at −20 °C or below. In parallel, capillary blood samples were obtained via finger-prick using a sterile lancet after warming the hand. The first drop of blood was discarded. The subsequent drop was used to prepare both dried blood spot (DBS) and volumetric absorptive microsampling (VAMS) samples. For DBS collection, capillary blood was applied directly to filter paper cards and allowed to dry at room temperature. For VAMS sampling, the device’s polymeric tip was gently touched to the blood drop until complete absorption occurred. All microsamples were air-dried and stored in sealed aluminum bags containing desiccant until analysis. Each DBS card and VAMS device was labeled with a unique patient identifier to ensure sample traceability. The samples were visually evaluated according to pre-analytical errors related to sampling using VAMS and DBSs. Hematocrit values in microsamples (VAMS and DBS) were determined using the methodology described in a previous study [22].

### 4.6. Method Validation Protocol

The LC–MS/MS method was validated in accordance with the International Council for Harmonization guideline for bioanalytical method validation (ICH M10), as adapted by the European Medicines Agency (EMA), and with the International Association of Therapeutic Drug Monitoring and Clinical Toxicology (IATDMCT) recommendations for microsampling approaches [9,10]. In addition, the Matuszewski et al. protocol for evaluating matrix effects, absolute recovery, and process efficiency was adapted for validation purposes [11].

Validation parameters included selectivity, calibration model, lower limit of quantification (LLOQ), accuracy, precision, carry-over, recovery, matrix effect, absolute recovery, process efficiency, and analyte stability [9,10,11].

Selectivity was assessed using blank samples from at least six independent sources of each matrix to confirm the absence of interfering peaks at the retention times of the analytes and internal standards. Interference was required to be ≤20% of the analyte response at the LLOQ and ≤5% of the internal standard response [9,10].

Calibration curves were constructed in the relevant biological matrices using analyte-to-internal standard peak-area ratios and appropriate regression models. At least 75% of non-zero calibration standards, including the LLOQ and the upper limit of quantification (ULOQ), were required to meet acceptance criteria of ±15% of nominal concentrations (±20% at the LLOQ) [9,10].

The LLOQ was defined as the lowest concentration that could be quantified with accuracy within ±20% of the nominal value and with a precision not exceeding a 20% coefficient of variation (CV), while providing an adequate signal-to-noise ratio for reliable quantification [9,10].

Within-run (intra-day) and between-run (inter-day) accuracy and precision were evaluated using quality control (QC) samples at four concentration levels (LLOQ, low, medium, and high) across multiple validation runs. Mean concentrations were required to be within ±15% of nominal values (±20% at LLOQ), with precision ≤ 15% CV (≤20% at LLOQ). At least 67% of QC samples, and at least 50% at each QC level, were required to meet these criteria [9,10].

Carry-over was assessed by injecting blank samples immediately after the highest calibration standard. The response in blank samples was required to be ≤20% of the analyte response at the LLOQ and ≤5% of the internal standard response [9,10].

Matrix effects, recovery, and process efficiency were assessed by comparing analyte responses from neat standard solutions (A), post-extraction spiked samples (B), and pre-extraction spiked samples (C). The absolute matrix effect (ME) was calculated as B/A × 100, recovery (RE) as C/B × 100, and process efficiency (PE) as C/A × 100. The matrix factor (MF) was defined as the ratio of analyte response in post-extraction spiked samples to that in neat solutions (MF = B/A). To account for variability, the internal standard–normalized matrix factor (IS-normalized MF) was obtained by dividing the analyte MF by the internal standard MF. Relative matrix effects were evaluated by analyzing the variability of the IS-normalized MF across different matrices and expressed as the coefficient of variation (CV). Matrix effects were deemed acceptable when the CV of the IS-normalized MF did not exceed 15%. This method allows differentiation between ion suppression or enhancement and extraction efficiency, ensuring robustness across various biological matrices [9,10,11].

Stability was assessed under diverse conditions, such as short-term (benchtop), autosampler, freeze–thaw, and long-term storage, for each matrix type. Analytes were deemed stable if their measured concentrations stayed within ±15% of the nominal values [9,10].

For dried blood spot (DBS) and volumetric absorptive microsampling (VAMS) matrices, additional validation experiments were conducted in accordance with IATDMCT recommendations. This included evaluation of hematocrit effects, spot or tip homogeneity, sample volume consistency, and stability during drying and storage. The influence of hematocrit and sampling conditions was considered acceptable if deviations in measured concentrations remained within ±15% [9,10].

An analytical run was deemed valid if at least 75% of calibration standards and 67% of QC samples met the set acceptance criteria. Study samples were analyzed exclusively within accepted batches, and any reanalysis was conducted in accordance with predefined standard operating procedures [9,10].

## 5. Conclusions

In conclusion, this study shows that volumetric absorptive microsampling can be successfully incorporated into a regulatory-compliant LC–MS/MS workflow for measuring methotrexate polyglutamates. The approach overcomes key analytical and pre-analytical challenges associated with microsampling of intracellular metabolites and broadens MTXPG analysis beyond traditional laboratory settings. By allowing accurate measurement from capillary blood, it offers a practical platform for decentralized monitoring of methotrexate exposure and patient adherence, while ensuring analytical consistency across multiple sample types in line with current regulatory standards. The strong correlation between VAMS and DBS confirms their interchangeable use in capillary sampling. Nevertheless, the observed differences relative to erythrocyte measurements reflect biological distribution factors rather than analytical errors, meaning that capillary-based results should be interpreted in a matrix-specific context. Therefore, capillary microsampling should be viewed as a complementary method rather than a direct replacement for RBC-based testing. Overall, these results support the potential and scalability of microsampling for decentralized MTXPG testing in therapeutic drug monitoring. Future research should include prospective validation with clinical outcomes such as treatment response, adherence, and toxicity, to identify clinically relevant, matrix-specific therapeutic thresholds. Such developments are crucial to integrating MTXPG monitoring into personalized treatments and pharmacometric dose optimization in rheumatoid arthritis.

## Figures and Tables

**Table 1:** Mean calibration curve parameters, including slope, intercept, and coefficient of determination (R^2^), for MTX and MTXPG_2–7_ in red blood cells (RBCs), whole blood (WB), volumetric absorptive microsampling (VAMS), and dried blood spots (DBSs). Values are presented as mean ± standard deviation (SD) obtained from six independent calibration curves (n = 6).

Analyte	Slope (RBC)	Intercept (RBC)	R^2^ (RBC)	Slope (WB)	Intercept (WB)	R^2^ (WB)	Slope (VAMS)	Intercept (VAMS)	R^2^ (VAMS)	Slope (DBS)	Intercept (DBS)	R^2^ (DBS)
MTX	0.0108 ± 0.0004	0.0021 ± 0.0003	0.9994 ± 0.0003	0.0103 ± 0.0004	0.0025 ± 0.0004	0.9992 ± 0.0004	0.0099 ± 0.0005	0.0030 ± 0.0005	0.9989 ± 0.0005	0.0096 ± 0.0005	0.0034 ± 0.0006	0.9987 ± 0.0005
MTXPG_2_	0.0102 ± 0.0004	0.0023 ± 0.0003	0.9993 ± 0.0003	0.0098 ± 0.0004	0.0028 ± 0.0004	0.9991 ± 0.0004	0.0093 ± 0.0005	0.0033 ± 0.0005	0.9988 ± 0.0005	0.0090 ± 0.0005	0.0038 ± 0.0006	0.9985 ± 0.0006
MTXPG_3_	0.0095 ± 0.0004	0.0026 ± 0.0004	0.9992 ± 0.0004	0.0091 ± 0.0004	0.0031 ± 0.0004	0.9990 ± 0.0004	0.0087 ± 0.0005	0.0037 ± 0.0005	0.9987 ± 0.0005	0.0083 ± 0.0005	0.0042 ± 0.0007	0.9984 ± 0.0006
MTXPG_4_	0.0088 ± 0.0004	0.0029 ± 0.0004	0.9991 ± 0.0004	0.0084 ± 0.0004	0.0035 ± 0.0005	0.9989 ± 0.0005	0.0080 ± 0.0005	0.0041 ± 0.0006	0.9986 ± 0.0006	0.0076 ± 0.0006	0.0047 ± 0.0007	0.9983 ± 0.0006
MTXPG_5_	0.0081 ± 0.0004	0.0033 ± 0.0005	0.9990 ± 0.0005	0.0077 ± 0.0004	0.0039 ± 0.0005	0.9988 ± 0.0005	0.0072 ± 0.0005	0.0046 ± 0.0006	0.9985 ± 0.0006	0.0069 ± 0.0006	0.0052 ± 0.0008	0.9981 ± 0.0007
MTXPG_6_	0.0069 ± 0.0005	0.0038 ± 0.0005	0.9988 ± 0.0005	0.0065 ± 0.0005	0.0045 ± 0.0006	0.9986 ± 0.0006	0.0061 ± 0.0005	0.0053 ± 0.0007	0.9982 ± 0.0007	0.0057 ± 0.0006	0.0060 ± 0.0009	0.9979 ± 0.0008
MTXPG_7_	0.0062 ± 0.0005	0.0043 ± 0.0006	0.9986 ± 0.0006	0.0058 ± 0.0005	0.0050 ± 0.0007	0.9984 ± 0.0007	0.0054 ± 0.0006	0.0059 ± 0.0008	0.9980 ± 0.0008	0.0050 ± 0.0006	0.0067 ± 0.0009	0.9976 ± 0.0009

MTX, methotrexate; MTXPG_2–7_, methotrexate polyglutamates containing 2–7 glutamate residues; RBC, red blood cell; WB, whole blood; VAMS, volumetric absorptive microsampling; DBS, dried blood spot; R^2^, coefficient of determination; SD, standard deviation.

**Table 2:** Matrix factor (MF), absolute recovery (AR), and process efficiency (PE) for MTX and MTXPG_2–7_ in different blood matrices (RBC, WB, VAMS, DBS). Data are presented as mean ± standard deviation (SD) with coefficient of variation (CV%), (n = 6). Matrix factor (MF) reflects analyte to internal standard normalization of matrix effects, absolute recovery (AR) represents extraction efficiency, and process efficiency (PE) combines both parameters.

Matrix	Analyte	MF (LQC)	AR (LQC) [%]	PE (LQC) [%]	MF (HQC)	AR (HQC) [%]	PE (HQC) [%]
RBC	MTX	1.01 ± 0.03 (3.0%)	91.2 ± 3.5 (3.8%)	92.1 ± 3.2 (3.5%)	1.01 ± 0.02 (2.2%)	91.2 ± 2.8 (3.1%)	92.1 ± 2.6 (2.8%)
	MTXPG_2_	0.98 ± 0.03 (3.1%)	90.5 ± 3.2 (3.5%)	88.7 ± 3.8 (4.3%)	0.98 ± 0.02 (2.3%)	90.5 ± 2.6 (2.9%)	88.7 ± 3.0 (3.4%)
	MTXPG_3_	0.97 ± 0.03 (3.2%)	89.8 ± 3.6 (4.0%)	87.3 ± 3.9 (4.5%)	0.97 ± 0.02 (2.4%)	89.8 ± 2.9 (3.2%)	87.3 ± 3.1 (3.6%)
	MTXPG_4_	0.95 ± 0.04 (4.2%)	87.9 ± 3.8 (4.3%)	83.5 ± 4.5 (5.4%)	0.95 ± 0.03 (3.0%)	87.9 ± 3.1 (3.5%)	83.5 ± 3.6 (4.3%)
	MTXPG_5_	0.94 ± 0.04 (4.5%)	86.8 ± 4.2 (4.8%)	81.5 ± 4.8 (5.9%)	0.94 ± 0.03 (3.2%)	86.8 ± 3.4 (3.9%)	81.5 ± 3.8 (4.7%)
	MTXPG_6_	0.93 ± 0.05 (5.2%)	85.5 ± 4.8 (5.6%)	79.5 ± 5.5 (6.9%)	0.93 ± 0.04 (3.8%)	85.5 ± 3.9 (4.6%)	79.5 ± 4.4 (5.5%)
	MTXPG_7_	0.92 ± 0.05 (5.8%)	84.0 ± 5.5 (6.5%)	77.0 ± 6.5 (8.4%)	0.92 ± 0.04 (4.2%)	84.0 ± 4.5 (5.4%)	77.0 ± 5.2 (6.8%)
WB	MTX	0.98 ± 0.04 (4.1%)	90.0 ± 4.5 (5.0%)	88.6 ± 4.2 (4.7%)	0.98 ± 0.03 (3.0%)	90.0 ± 3.6 (4.0%)	88.6 ± 3.3 (3.7%)
	MTXPG_2_	0.95 ± 0.05 (5.2%)	88.4 ± 5.1 (5.8%)	84.8 ± 5.6 (6.6%)	0.95 ± 0.04 (3.8%)	88.4 ± 4.1 (4.6%)	84.8 ± 4.5 (5.3%)
	MTXPG_3_	0.94 ± 0.05 (5.5%)	87.0 ± 5.4 (6.2%)	82.0 ± 6.0 (7.3%)	0.94 ± 0.04 (4.0%)	87.0 ± 4.3 (5.0%)	82.0 ± 4.8 (5.9%)
	MTXPG_4_	0.93 ± 0.06 (6.2%)	85.5 ± 5.8 (6.8%)	79.5 ± 6.5 (8.2%)	0.93 ± 0.05 (4.5%)	85.5 ± 4.6 (5.4%)	79.5 ± 5.2 (6.5%)
	MTXPG_5_	0.91 ± 0.06 (6.8%)	84.0 ± 6.2 (7.4%)	76.5 ± 7.0 (9.2%)	0.91 ± 0.05 (5.0%)	84.0 ± 5.0 (6.0%)	76.5 ± 5.6 (7.3%)
	MTXPG_6_	0.90 ± 0.07 (7.5%)	82.5 ± 6.8 (8.2%)	74.0 ± 7.8 (10.5%)	0.90 ± 0.06 (5.6%)	82.5 ± 5.4 (6.5%)	74.0 ± 6.2 (8.4%)
	MTXPG_7_	0.89 ± 0.08 (8.5%)	81.0 ± 7.5 (9.3%)	71.5 ± 8.5 (11.9%)	0.89 ± 0.06 (6.4%)	81.0 ± 6.0 (7.4%)	71.5 ± 6.8 (9.5%)
VAMS	MTX	0.95 ± 0.05 (5.5%)	85.5 ± 6.0 (7.0%)	81.5 ± 6.5 (8.0%)	0.95 ± 0.04 (4.2%)	85.5 ± 4.8 (5.6%)	81.5 ± 5.2 (6.4%)
	MTXPG_2_	0.92 ± 0.06 (6.5%)	83.5 ± 6.8 (8.1%)	77.5 ± 7.5 (9.7%)	0.92 ± 0.05 (5.0%)	83.5 ± 5.4 (6.5%)	77.5 ± 6.0 (7.7%)
	MTXPG_3_	0.90 ± 0.07 (7.5%)	81.0 ± 7.5 (9.3%)	73.0 ± 8.2 (11.2%)	0.90 ± 0.05 (5.8%)	81.0 ± 6.0 (7.4%)	73.0 ± 6.6 (9.0%)
	MTXPG_4_	0.88 ± 0.08 (9.0%)	78.5 ± 8.5 (10.8%)	69.5 ± 9.5 (13.7%)	0.88 ± 0.06 (7.0%)	78.5 ± 6.8 (8.7%)	69.5 ± 7.6 (10.9%)
	MTXPG_5_	0.86 ± 0.09 (10.5%)	76.0 ± 9.5 (12.5%)	65.5 ± 10.5 (16.0%)	0.86 ± 0.07 (8.2%)	76.0 ± 7.6 (10.0%)	65.5 ± 8.4 (12.8%)
	MTXPG_6_	0.84 ± 0.10 (12.0%)	73.5 ± 10.5 (14.3%)	62.0 ± 11.5 (18.5%)	0.84 ± 0.08 (9.5%)	73.5 ± 8.4 (11.4%)	62.0 ± 9.2 (14.8%)
	MTXPG_7_	0.82 ± 0.11 (13.5%)	71.0 ± 11.5 (16.2%)	58.5 ± 12.5 (21.4%)	0.82 ± 0.09 (10.8%)	71.0 ± 9.2 (13.0%)	58.5 ± 10.0 (17.1%)
DBS	MTX	0.90 ± 0.06 (6.5%)	83.5 ± 7.0 (8.4%)	75.5 ± 7.5 (9.9%)	0.90 ± 0.05 (5.0%)	83.5 ± 5.6 (6.7%)	75.5 ± 6.0 (7.9%)
	MTXPG_2_	0.88 ± 0.07 (7.5%)	81.0 ± 7.8 (9.6%)	71.0 ± 8.5 (12.0%)	0.88 ± 0.05 (5.8%)	81.0 ± 6.2 (7.7%)	71.0 ± 6.8 (9.6%)
	MTXPG_3_	0.86 ± 0.08 (9.0%)	78.5 ± 8.5 (10.8%)	67.5 ± 9.5 (14.1%)	0.86 ± 0.06 (6.8%)	78.5 ± 6.8 (8.7%)	67.5 ± 7.6 (11.3%)
	MTXPG_4_	0.84 ± 0.09 (10.5%)	76.0 ± 9.5 (12.5%)	63.5 ± 10.5 (16.5%)	0.84 ± 0.07 (8.0%)	76.0 ± 7.6 (10.0%)	63.5 ± 8.4 (13.2%)
	MTXPG_5_	0.82 ± 0.10 (12.0%)	73.5 ± 10.5 (14.3%)	60.0 ± 11.5 (19.2%)	0.82 ± 0.08 (9.2%)	73.5 ± 8.4 (11.4%)	60.0 ± 9.2 (15.3%)
	MTXPG_6_	0.80 ± 0.11 (13.5%)	71.0 ± 11.5 (16.2%)	56.5 ± 12.5 (22.1%)	0.80 ± 0.09 (10.8%)	71.0 ± 9.2 (13.0%)	56.5 ± 10.0 (17.7%)
	MTXPG_7_	0.78 ± 0.12 (15.5%)	68.5 ± 12.5 (18.2%)	53.0 ± 13.5 (25.5%)	0.78 ± 0.10 (12.4%)	68.5 ± 10.0 (14.6%)	53.0 ± 10.8 (20.4%)

LQC, low quality control; HQC, high quality control; CV, coefficient of variation; SD, standard deviation; RBC, red blood cell; WB, whole blood; VAMS, volumetric absorptive microsampling; DBS, dried blood spot.

**Table 3:** Cross-validation for methotrexate polyglutamates (MTX, MTXPG_2–5_ and total MTXPG) across different blood sampling matrices (VAMS, DBS, RBC, and WB) using 40 paired samples. Passing–Bablok regression equations, slope (A) and intercept (B) with 95% confidence intervals (CI), Bland–Altman bias with 95% CI, percentage of paired samples within limits of agreement (LoA) and clinical agreement (CoA), and Spearman rank correlation coefficients (SRCC) with 95% CI are presented for each pairwise comparison.

Statistics	MTX	MTXPG_2_	MTXPG_3_	MTXPG_4_	MTXPG_5_	MTXPG_total_
**VAMS vs. DBS** [n = 40 paired samples]						
Passing–Bablok formula	VAMS = 1.06(DBS) − 1.26	VAMS = 1.07(DBS) + 0.94	VAMS = 1.04(DBS) − 2.21	VAMS = 1.03(DBS) − 1.07	VAMS = 0.95(DBS) + 0.05	VAMS = 1.06(DBS) − 7.82
Slope (A) with 95% CI	1.0600 (0.9841–1.1870)	1.0739 (0.9600–1.1083)	1.0428 (0.9862–1.1897)	1.0309 (0.9868–1.0987)	0.9532 (0.9162–1.0599)	1.0695 (0.8697–1.0828)
Intercept (B) with 95% CI	−1.2560 (−1.7087–0.4151)	0.9428 (−1.8479–0.5175)	−2.2114 (−4.0643–0.5133)	−1.0708 (−1.8599–1.0559)	0.0457 (−0.7523–0.3090)	−7.8211 (−10.0090–7.8343)
Bland–Altman bias (VAMS–DBS) with 95% CI [%]	−3.15 (−6.92–0.84)	0.36 (−2.19–2.90)	−4.21 (−7.89–0.75)	−3.21 (−6.86–0.45)	−2.50 (−6.43–1.43)	−1.12 (−4.32–2.37)
%-of paired samples in LoA (±20%)	95.00	100.00	90.00	87.50	87.50	100.00
%-of paired samples in CoA (±15%)	85.00	95.00	77.50	87.50	75.00	100.00
SRCC (95% CI)	0.994 (0.989–0.997)	0.989 (0.890–0.994)	0.982 (0.907–0.991)	0.990 (0.982–0.995)	0.969 (0.942–0.984)	0.980 (0.962–0.989)
**RBC vs. VAMS** [n = 40 paired samples]						
Passing–Bablok formula	RBC = 1.18(VAMS) + 0.48	RBC = 1.16(VAMS) − 0.88	RBC = 1.27(VAMS) + 0.07	RBC = 1.19(VAMS) + 1.31	RBC = 1.18(VAMS) + 0.33	RBC = 1.14(VAMS) + 8.03
Slope (A) with 95% CI	1.1823 (1.0688–1.3145)	1.1554 (1.0529–1.3603)	1.2676 (1.0797–1.4791)	1.1874 (1.0886–1.2796)	1.1819 (1.0288–1.3651)	1.1393 (1.0181–1.2697)
Intercept (B) with 95% CI	0.4770 (−1.4826–1.8878)	−0.8841 (−3.9582–1.6339)	0.0668 (−5.4448–5.6379)	1.3080 (−1.4146–4.1013)	0.3305 (−1.4108–2.0309)	8.0343 (−7.7948–19.8022)
Bland–Altman bias (VAMS–RBC) with 95% CI [%]	−20.10 (−32.33–−7.69)	−8.91 (−18.87–1.05)	−23.16 (−31.16–−15.16)	−23.22 (−31.54–−14.91)	−28.12 (−40.58–−15.66)	−19.59 (−22.82–−16.36)
%-of paired samples in LoA (±20%)	50.00	57.50	37.50	45.00	62.50	52.50
%-of paired samples in CoA (±15%)	37.50	50.00	25.00	32.50	55.00	27.50
SRCC (95% CI)	0.899 (0.816–0.946)	0.888 (0.798–0.940)	0.886 (0.794–0.939)	0.964 (0.932–0.981)	0.811 (0.669–0.896)	0.885 (0.791–0.938)
**WB vs. VAMS** [n = 40 paired samples]						
Passing–Bablok formula	WB = 1.01(VAMS) − 0.02	WB = 0.99(VAMS) − 0.84	WB = 0.99(VAMS) + 1.24	WB = 1.08(VAMS) − 2.16	WB = 0.96(VAMS) − 0.16	WB = 1.00(VAMS) − 1.39
Slope (A) with 95% CI	1.0089 (0.9365–1.0982)	0.9861 (0.8732–1.1424)	0.9927 (0.9008–1.1529)	1.0806 (0.9865–1.1798)	0.9609 (0.8433–1.1182)	0.9970 (0.8678–1.1573)
Intercept (B) with 95% CI	−0.02088 (−1.3820–0.6993)	−0.8842 (−3.1787–1.8256)	1.2395 (−4.3464–3.0594)	−2.1595 (−4.0614–−0.0383)	−0.1647 (−1.1924–0.7052)	−1.3891 (−15.3807–12.0818)
Bland–Altman bias (WB–VAMS) with 95% CI [%]	−7.31 (−19.99–5.39)	−4.21 (−13.08–4.65)	2.71 (−6.60–12.01)	−2.83 (−9.99–4.34)	−4.41 (−13.73–4.90)	−0.45 (−3.44–2.55)
%-of paired samples in LoA (±20%)	75.00	65.00	70.00	87.50	62.50	95.00
%-of paired samples in CoA (±15%)	62.50	52.50	65.00	70.00	55.00	92.50
SRCC (95% CI)	0.902 (0.822–0.948)	0.887 (0.795–0.939)	0.894 (0.807–0.943)	0.976 (0.955– 0.987)	0.937 (0.884–0.967)	0.938 (0.885–0.967)
**RBC vs. DBS** [n = 40 paired samples]						
Passing–Bablok formula	RBC = 1.25(DBS) − 0.38	RBC = 1.27(DBS) − 2.58	RBC = 1.32(DBS) − 1.25	RBC = 1.21(DBS) + 0.48	RBC = 1.14(DBS) + 0.24	RBC = 1.23(DBS) − 0.67
Slope (A) with 95% CI	1.2536 (1.1247–1.4095)	1.2655 (1.1306–1.4473)	1.3205 (1.1248–1.5713)	1.2084 (1.0786–1.3100)	1.1389 (0.9741–1.3505)	1.2271 (1.0803–1.3634)
Intercept (B) with 95% CI	−0.3780 (−2.3861–1.6575)	−2.5766 (−6.7601–0.1625)	−1.2456 (−9.0735–3.5382)	0.4808 (−2.2021–3.2882)	0.2425 (−1.4280–1.6598)	−0.6717 (−20.6865–14.6104)
Bland–Altman bias (DBS–RBC) with 95% CI [%]	−17.44 (−29.92–−4.96)	−9.24 (−19.61–1.12)	−19.01 (−27.52–−10.49)	−20.26 (−28.70–−11.83)	−25.59 (−38.66–−12.51)	−18.49 (−21.77–−15.20)
%-of paired samples in LoA (±20%)	45.00	65.00	60.00	47.50	37.50	52.50
%-of paired samples in CoA (±15%)	32.50	42.50	45.00	35.00	25.00	37.50
SRCC (95% CI)	0.898 (0.815–0.945)	0.890 (0.801–0.941)	0.890 (0.800–0.941)	0.972 (0.947–0.985)	0.795 (0.643–0.887)	0.870 (0.767–0.930)
**WB vs. DBS** [n = 40 paired samples]						
Passing–Bablok formula	WB = 1.07(DBS) − 1.30	WB = 1.06(DBS) − 1.53	WB = 1.05(DBS) − 1.05	WB = 1.12(DBS) − 3.05	WB = 0.91(DBS) + 0.11	WB = 1.04(DBS) − 6.36
Slope (A) with 95% CI	1.0732 (1.0047–1.1700)	1.0597 (0.9173–1.2312)	1.0475 (0.9283–1.2298)	1.1237 (0.9868–1.2327)	0.9122 (0.7972–1.0587)	1.0393 (0.9074–1.2114)
Intercept (B) with 95% CI	−1.3027 (−2.9469–0.2035)	−1.5315 (−4.4669–1.1326)	−1.0505 (−7.8762–2.0825)	−3.0511 (−5.2248–−0.7523)	0.1075 (−0.8381–0.6949)	−6.3563 (−24.2782–6.9594)
Bland–Altman bias (WB–DBS) with 95% CI [%]	10.31 (−2.41–23.03)	3.81 (−5.49–13.12)	1.37 (−8.25–11.00)	5.82 (−2.46–14.10)	7.06 (−1.12–15.25)	1.56 (−1.55–4.67)
%-of paired samples in LoA (±20%)	72.50	72.50	72.50	87.50	65.00	97.50
%-of paired samples in CoA (±15%)	55.00	57.50	62.50	65.00	52.50	90.00
SRCC (95% CI)	0.905 (0.826–0.949)	0.888 (0.797– 0.940)	0.883 (0.788–0.937)	0.971 (0.945–0.985)	0.947 (0.901–0.972)	0.931 (0.873–0.963)
**RBC vs. WB** [n = 40 paired samples]						
Passing–Bablok formula	RBC = 1.18(WB) + 0.55	RBC = 1.15(WB) + 0.13	RBC = 1.18(WB) − 0.11	RBC = 1.08(WB) + 4.02	RBC = 1.27(WB) + 0.65	RBC = 1.12(WB) + 9.40
Slope (A) with 95% CI	1.1779 (1.0539–1.2606)	1.1538 (1.0291–1.2460)	1.1819 (1.0446–1.3415)	1.0880 (0.9878–1.1809)	1.2721 (1.0798–1.4119)	1.1159 (1.0197–1.2609)
Intercept (B) with 95% CI	0.5537 (−0.0999–2.1948)	0.1325 (−1.9174–1.8391)	−0.1132 (−4.3174–6.0428)	4.0159 (1.2775–6.1056)	0.6549 (−0.9723–1.8963)	9.4041 (−3.7703–20.8210)
Bland–Altman bias (RBC–WB) with 95% CI [%]	27.59 (12.56–38.59)	13.34 (7.88–18.80)	20.60 (13.74–27.46)	26.28 (20.13–32.44)	33.18 (21.32–45.03)	20.04 (17.13–23.22)
%-of paired samples in LoA (±20%)	37.50	60.00	37.50	47.50	37.50	52.50
%-of paired samples in CoA (±15%)	25.00	42.50	25.00	25.00	25.00	37.50
SRCC (95% CI)	0.897 (0.812–0.944)	0.961 (0.926–0.979)	0.922 (0.856–0.958)	0.972 (0.947–0.985)	0.847 (0.727–0.917)	0.907 (0.830–0.950)

MTXPG, methotrexate polyglutamate; MTXPG_total_, sum of MTX and MTXPG_2–5_; VAMS, volumetric absorptive microsampling; DBS, dried blood spot; RBC, red blood cell; WB, whole blood.

**Table 4:** Multiple reaction monitoring (MRM) transitions and optimized mass spectrometric parameters for methotrexate (MTX) and its polyglutamates (MTXPG_2–7_) with corresponding stable isotope-labeled internal standards. Analyses were performed using electrospray ionization (ESI) in positive ion mode. Quantitative transitions (Q1 → Q3; *m*/*z*), collision energy (CE), declustering potential (DP), entrance potential (EP), and collision cell exit potential (CXP) are reported. Singly charged precursor ions ([M+H]^+^) were monitored for MTXPG_1–5_, whereas doubly charged ions ([M+2H]^2+^) were used for MTXPG_6_ and MTXPG_7_. Retention times are provided in minutes under the described chromatographic conditions.

Analyte	ESI Mode	Q1 Quantitative (*m*/*z*)	Q3 Quantitative (*m*/*z*)	CE [eV]	DP [eV]	EP [eV]	CXP [eV]	Retention Time [min]
MTX MTX-d_3_	+	455.1 458.1	308.2 311.1	23 26	60 50	8 8	10 10	5.70
MTXPG2 MTXPG_2_-d_3_	+	584.5 587.5	308.2 311.1	32 36	80 80	10 10	10 10	3.98
MTXPG_3_ MTXPG_3_-d_3_	+	713.5 716.7	308.2 311.1	39 43	110 90	8 8	10 10	3.27
MTXPG_4_ MTXPG_4_-d_3_	+	842.3 845.5	308.2 311.1	51 55	100 100	10 8	10 10	2.39
MTXPG_5_ MTXPG_5_-d_3_	+	971.4 974.2	308.2 311.1	58 56	70 70	8 6	10 10	2.14
MTXPG_6_ MTXPG_6_-d_3_	2+	550.7 552.3	308.2 311.1	23 23	90 90	6 6	10 10	1.10
MTXPG_7_ MTXPG_7_-d_3_	2+	615.2 617.0	308.2 311.1	31 31	80 90	6 6	10 10	0.93

## Data Availability

Data will be made available upon reasonable request from the corresponding author.

## Supplementary Materials

The following supporting information can be downloaded at: https://www.mdpi.com/article/10.3390/ijms27104429/s1.

## Abbreviations

The following abbreviations are used in this manuscript:

ANCOVA	Analysis of covariance
BA	Bland–Altman bias analysis
CAD	Collision gas
CE	Collision energy
CI	Confidence interval
CoA	Clinical agreement
CV	Coefficient of variation
DBS	Dried blood spot
DP	Declustering potential
EP	Entrance potential
ESI	Electrospray ionization
Hct	Hematocrit
HQC	High quality control
ICH	International Council for Harmonization
ISR	Incurred sample reanalysis
LC–MS/MS	Liquid chromatography–tandem mass spectrometry
LLOQ	Lower limit of quantification
LoA	Limits of agreement
LQC	Low quality control
MQC	Medium quality control
MRM	Multiple reaction monitoring
MTX	Methotrexate
MTXPG	Methotrexate polyglutamates
PB	Passing–Bablok regression
RBC	Red blood cells
SD	Standard deviation
SPE	Solid-phase extraction
SRCC	Spearman rank correlation coefficient
TDM	Therapeutic drug monitoring
ULOQ	Upper limit of quantification
VAMS	Volumetric absorptive microsampling
WB	Whole blood

## Appendix A

**Table A1:** Accuracy and precision of MTX and MTXPG2–7 in RBC, WB, VAMS, and DBS matrices. Results are shown at seven concentration levels: LLOQ, LQC, MQC1, MQC2, MQC3, HQC, and ULOQ. Values are presented as mean concentration ± SD, precision CV (%), and accuracy (%), based on six replicates per level. Accuracy was defined as the percentage of nominal concentration recovered, and precision was expressed as the coefficient of variation. Acceptance criteria were ±15% for accuracy and CV ≤ 15% at QC levels above LLOQ, and ±20% for accuracy and CV ≤ 20% at LLOQ. MTXPG6 and MTXPG7 showed weaker performance at low concentrations, particularly in VAMS and DBS.

Analyte	LLOQ	LQC	MQC1	MQC2	MQC3	HQC	ULOQ
RBC							
MTX	0.1049 ± 0.0073 7.0% (104.9%)	0.2526 ± 0.0235 9.3% (101.1%)	3.4984 ± 0.1033 3.0% (100.0%)	7.6608 ± 0.2928 3.8% (102.1%)	25.7020 ± 1.6250 6.3% (102.8%)	120.7896 ± 2.3178 1.9% (96.6%)	151.5530 ± 3.5090 2.3% (101.0%)
MTXPG2	0.1022 ± 0.0105 10.3% (102.2%)	0.2558 ± 0.0131 5.1% (102.3%)	3.6422 ± 0.2113 5.8% (104.1%)	7.6030 ± 0.1881 2.5% (101.4%)	25.1940 ± 0.6726 2.7% (100.8%)	129.0312 ± 12.8186 9.9% (103.2%)	151.0542 ± 7.5528 5.0% (100.7%)
MTXPG3	0.1007 ± 0.0051 5.0% (100.7%)	0.2414 ± 0.0167 6.9% (96.6%)	3.5172 ± 0.0876 2.5% (100.5%)	7.7460 ± 0.3895 5.0% (103.3%)	24.2530 ± 1.4130 5.8% (97.0%)	123.6252 ± 3.1203 2.5% (98.9%)	153.0932 ± 8.2503 5.4% (102.1%)
MTXPG4	0.1002 ± 0.0110 11.0% (100.2%)	0.2630 ± 0.0171 6.5% (105.2%)	3.4678 ± 0.1630 4.7% (99.1%)	7.6254 ± 0.5138 6.7% (101.7%)	25.3232 ± 1.2258 4.8% (101.3%)	117.4282 ± 10.5467 9.0% (93.9%)	150.6404 ± 7.2857 4.8% (100.4%)
MTXPG5	0.0938 ± 0.0130 13.8% (93.8%)	0.2410 ± 0.0359 14.9% (96.4%)	3.3380 ± 0.1247 3.7% (95.4%)	7.4478 ± 0.4187 5.6% (99.3%)	24.7490 ± 1.1681 4.7% (99.0%)	123.7618 ± 9.2201 7.4% (99.0%)	149.9288 ± 8.6497 5.8% (100.0%)
MTXPG6	0.0915 ± 0.0148 16.2% (91.5%)	0.2385 ± 0.0385 16.1% (95.4%)	3.2800 ± 0.1850 5.6% (93.7%)	7.3600 ± 0.5200 7.1% (98.1%)	24.1000 ± 1.4800 6.1% (96.4%)	121.5 ± 10.8 8.9% (97.2%)	148.2 ± 9.8 6.6% (98.8%)
MTXPG7	0.0880 ± 0.0165 18.7% (88.0%)	0.2320 ± 0.0415 17.9% (92.8%)	3.1500 ± 0.2100 6.7% (90.0%)	7.2000 ± 0.6100 8.5% (96.0%)	23.4000 ± 1.7200 7.3% (93.6%)	119.8 ± 12.4 10.3% (95.8%)	146.0 ± 11.2 7.7% (97.3%)
WB							
MTX	0.0968 ± 0.0061 6.3% (96.8%)	0.2556 ± 0.0124 4.8% (102.3%)	3.5900 ± 0.3260 9.1% (102.6%)	7.3426 ± 0.2729 3.7% (97.9%)	24.2998 ± 1.8247 7.5% (97.2%)	124.5850 ± 9.6347 7.7% (99.7%)	149.1178 ± 15.3609 10.3% (99.4%)
MTXPG2	0.0916 ± 0.0136 14.8% (91.6%)	0.2439 ± 0.0211 8.6% (97.6%)	3.4894 ± 0.2166 6.2% (99.7%)	7.4808 ± 0.7257 9.7% (99.7%)	24.3344 ± 1.0846 4.5% (97.3%)	124.8896 ± 7.7887 6.2% (99.9%)	149.0724 ± 6.7404 4.5% (99.4%)
MTXPG3	0.0949 ± 0.0124 13.0% (94.9%)	0.2438 ± 0.0078 3.2% (97.5%)	3.3818 ± 0.4079 12.1% (96.6%)	7.2426 ± 0.5049 7.0% (96.6%)	23.4086 ± 1.3661 5.8% (93.6%)	122.2062 ± 4.8368 4.0% (97.8%)	140.0256 ± 5.9517 4.3% (93.4%)
MTXPG4	0.0904 ± 0.0089 9.9% (90.4%)	0.2380 ± 0.0173 7.3% (95.2%)	3.5010 ± 0.3690 10.5% (100.0%)	7.5608 ± 0.8362 11.1% 100.8%)	25.0440 ± 0.3338 1.3% (100.2%)	121.9854 ± 11.8244 9.7% (97.6%)	145.3432 ± 16.1151 11.1% (96.9%)
MTXPG5	0.0936 ± 0.0131 14.0% (93.6%)	0.2411 ± 0.0264 11.0% (96.4%)	3.4270 ± 0.2580 7.5% (97.9%)	7.3410 ± 0.5416 7.4% (97.9%)	24.9288 ± 1.2109 4.9% (99.7%)	118.5600 ± 8.0714 6.8% (94.8%)	153.6762 ± 13.1993 8.6% (102.5%)
MTXPG6	0.0890 ± 0.0155 17.4% (89.0%)	0.2350 ± 0.0390 16.6% (94.0%)	3.2400 ± 0.2500 7.7% (92.6%)	7.2100 ± 0.6200 8.6% (96.1%)	23.9000 ± 1.6500 6.9% (95.6%)	120.2 ± 11.9 9.9% (96.2%)	147.8 ± 10.8 7.3% (98.5%)
MTXPG7	0.0855 ± 0.0170 19.9% (85.5%)	0.2280 ± 0.0435 19.1% (91.2%)	3.1000 ± 0.2900 9.4% (88.6%)	7.0500 ± 0.7100 10.1% (94.0%)	23.1000 ± 1.8900 8.2% (92.4%)	118.4 ± 13.6 11.5% (94.7%)	145.2 ± 12.6 8.7% (96.8%)
VAMS							
MTX	0.0979 ± 0.0131 13.3% (97.9%)	0.2457 ± 0.0137 5.6% (98.3%)	3.4956 ± 0.2473 7.1% (99.9%)	7.5090 ± 0.6629 8.8% (100.1%)	23.1048 ± 1.6489 7.1% (92.4%)	124.8230 ± 8.8987 7.1% (99.9%)	147.2214 ± 8.4469 5.7% (98.1%)
MTXPG2	0.0911 ± 0.0126 13.9% (91.1%)	0.2694 ± 0.0229 8.5% (107.8%)	3.6778 ± 0.1781 4.8% (105.1%)	7.6492 ± 0.7030 9.2% (102.0%)	25.0592 ± 0.7586 3.0% (100.2%)	118.1328 ± 12.5269 10.6% (94.5%)	136.4760 ± 7.3739 5.4% (91.0%)
MTXPG3	0.1067 ± 0.0125 11.7% (106.7%)	0.2384 ± 0.0173 7.3% (95.3%)	3.6144 ± 0.1781 4.9% (103.3%)	7.4304 ± 0.3620 4.9% (99.1%)	24.9720 ± 1.0520 4.2% (99.9%)	124.4002 ± 9.6791 7.8% (99.5%)	133.4918 ± 15.0260 11.3% (89.0%)
MTXPG4	0.0914 ± 0.0138 15.1% (91.4%)	0.2352 ± 0.0425 18.1% (94.1%)	3.3254 ± 0.4647 14.0% (95.0%)	6.8566 ± 0.3593 5.2% (91.4%)	25.2920 ± 1.4718 5.8% (101.2%)	120.0044 ± 7.3328 6.1% (96.0%)	144.7310 ± 10.9200 7.5% (96.5%)
MTXPG5	0.0987 ± 0.0132 13.4% (98.7%)	0.2412 ± 0.0120 5.0% (96.5%)	3.4918 ± 0.3141 9.0% (99.8%)	7.9336 ± 0.5921 7.5% (105.8%)	23.4962 ± 2.6443 11.3% (94.0%)	118.7350 ± 13.6218 11.5% (95.0%)	143.1322 ± 11.9927 8.4% (95.4%)
MTXPG6	0.0875 ± 0.0168 19.2% (87.5%)	0.2310 ± 0.0420 18.2% (92.4%)	3.2000 ± 0.2700 8.4% (91.4%)	7.1500 ± 0.6500 9.1% (95.3%)	23.6000 ± 1.8800 8.0% (94.4%)	119.0 ± 12.9 10.8% (95.2%)	146.5 ± 11.8 8.1% (97.7%)
MTXPG7	0.0830 ± 0.0185 22.3% (83.0%)	0.2250 ± 0.0460 20.4% (90.0%)	3.0500 ± 0.3200 10.5% (87.1%)	6.9500 ± 0.7800 11.2% (92.7%)	22.9000 ± 2.0500 9.0% (91.6%)	117.5 ± 14.8 12.6% (94.0%)	144.0 ± 13.5 9.4% (96.0%)
DBS							
MTX	0.1017 ± 0.0081 8.0% (101.7%)	0.2588 ± 0.0169 6.5% (103.5%)	3.5898 ± 0.4216 11.7% (102.6%)	7.2464 ± 0.6762 9.3% (96.6%)	24.3736 ± 2.2808 9.4% (97.5%)	121.0608 ± 14.4000 11.9% (96.8%)	150.4202 ± 14.5464 9.7% (100.3%)
MTXPG2	0.1005 ± 0.0079 7.9% (100.5%)	0.2523 ± 0.0232 9.2% (100.9%)	3.0736 ± 0.3622 11.8% (87.8%)	7.3140 ± 0.8707 11.9% (97.5%)	23.1780 ± 1.7436 7.5% (92.7%)	122.7276 ± 5.5382 4.5% (98.2%)	153.1078 ± 15.1344 9.9% (102.1%)
MTXPG3	0.0882 ± 0.0102 11.6% (88.2%)	0.2357 ± 0.0296 12.5% (94.3%)	3.3244 ± 0.2156 6.5% (95.0%)	7.1426 ± 0.9558 13.4% (95.2%)	24.6080 ± 1.6445 6.7% (98.4%)	130.1532 ± 6.2395 4.8% (104.1%)	147.6022 ± 20.2478 13.7% (98.4%)
MTXPG4	0.0967 ± 0.0075 7.8% (96.7%)	0.2589 ± 0.0256 9.9% (103.5%)	3.3622 ± 0.2969 8.8% (96.1%)	7.8180 ± 0.6539 8.4% (104.2%)	25.9644 ± 3.9085 15.1% (103.9%)	113.6238 ± 13.7816 12.1% (90.9%)	143.8608 ± 10.0835 7.0% (95.9%)
MTXPG5	0.0997 ± 0.0066 6.6% (99.7%)	0.2211 ± 0.0121 5.5% (88.4%)	3.1430 ± 0.3273 10.4% (89.8%)	7.0250 ± 1.0348 14.7% (93.7%)	23.3758 ± 2.9770 12.7% (93.5%)	126.7292 ± 11.4610 9.0% (101.4%)	146.8174 ± 22.1487 15.1% (97.9%)
MTXPG6	0.0850 ± 0.0175 20.6% (85.0%)	0.2280 ± 0.0455 20.0% (91.2%)	3.1500 ± 0.3100 9.8% (90.0%)	7.0000 ± 0.8200 11.7% (93.3%)	23.2000 ± 2.2500 9.7% (92.8%)	118.2 ± 15.2 12.9% (94.6%)	145.5 ± 14.8 10.2% (97.0%)
MTXPG7	0.0800 ± 0.0195 24.4% (80.0%)	0.2200 ± 0.0500 22.7% (88.0%)	3.0000 ± 0.3500 11.7% (85.7%)	6.8500 ± 0.9500 13.9% (91.3%)	22.5000 ± 2.4500 10.9% (90.0%)	116.8 ± 16.5 14.1% (93.4%)	143.0 ± 16.2 11.3% (95.3%)

LLOQ, lower limit of quantification; LQC, low quality control; MQC1–MQC3, medium quality control levels; HQC, high quality control; ULOQ, upper limit of quantification; CV, coefficient of variation; SD, standard deviation; RBC, red blood cell; WB, whole blood; VAMS, volumetric absorptive microsampling; DBS, dried blood spot.
