# Supplementary material for: Multi-Matrix LC–MS/MS Validation of Methotrexate Polyglutamates: Comparison of VAMS, DBS, and Conventional Blood Sampling in Rheumatoid Arthritis
Source: Int J Mol Sci. 2026 May 15;27(10):4429. doi: 10.3390/ijms27104429 (PMC13207903; doi:10.3390/ijms27104429)
Supplement: Supplementary file 1 [file ijms-27-04429-s001.zip › ijms-4303551-supplementary.pdf]

Supplementary File 1
Analytical and Clinical Validation of a Multi-matrix LC–MS/MS Method for Methotrexate Polyglutamate Quantification in Capillary Blood Using Volumetric Absorptive Microsampling and Dried Blood Spots in Rheumatoid Arthritis Patients. PB – Passing–Bablok regression; BA – Bland–Altman analysis; DBS – dried blood spots; WB– whole blood; RBC – red blood cells; MTXPG – methotrexate polyglutamates.

Line designation: In Passing–Bablok regression plots, the solid black line represents the regression line, the grey shaded area indicates the 95% confidence interval, and the light grey diagonal line represents the line of identity. In Bland–Altman plots, the green solid line indicates the mean bias, the black dashed lines indicate the limits of agreement (mean ± 1.96 SD), the red solid lines indicate the predefined acceptance limits, and the grey dotted horizontal line represents zero difference.

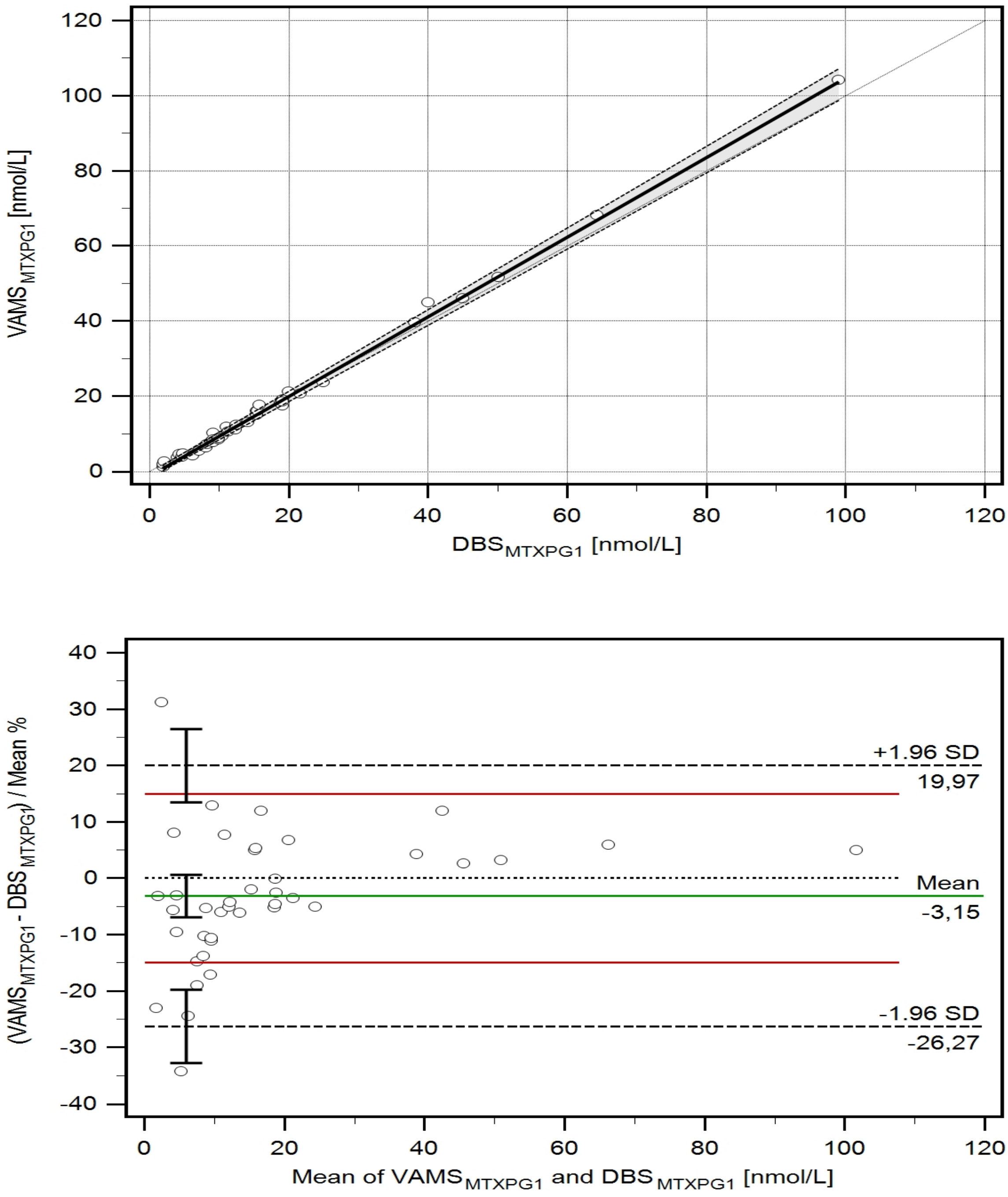

Fig. S1A–B. MTXPG1: PB regression (A) and BA analysis (B), DBS vs RBC.

*In the supplementary method-comparison figures, MTXPG1 denotes the parent methotrexate compound (MTX) for consistency with the original plotting workflow; in the manuscript text and tables, this analyte is referred to as MTX.

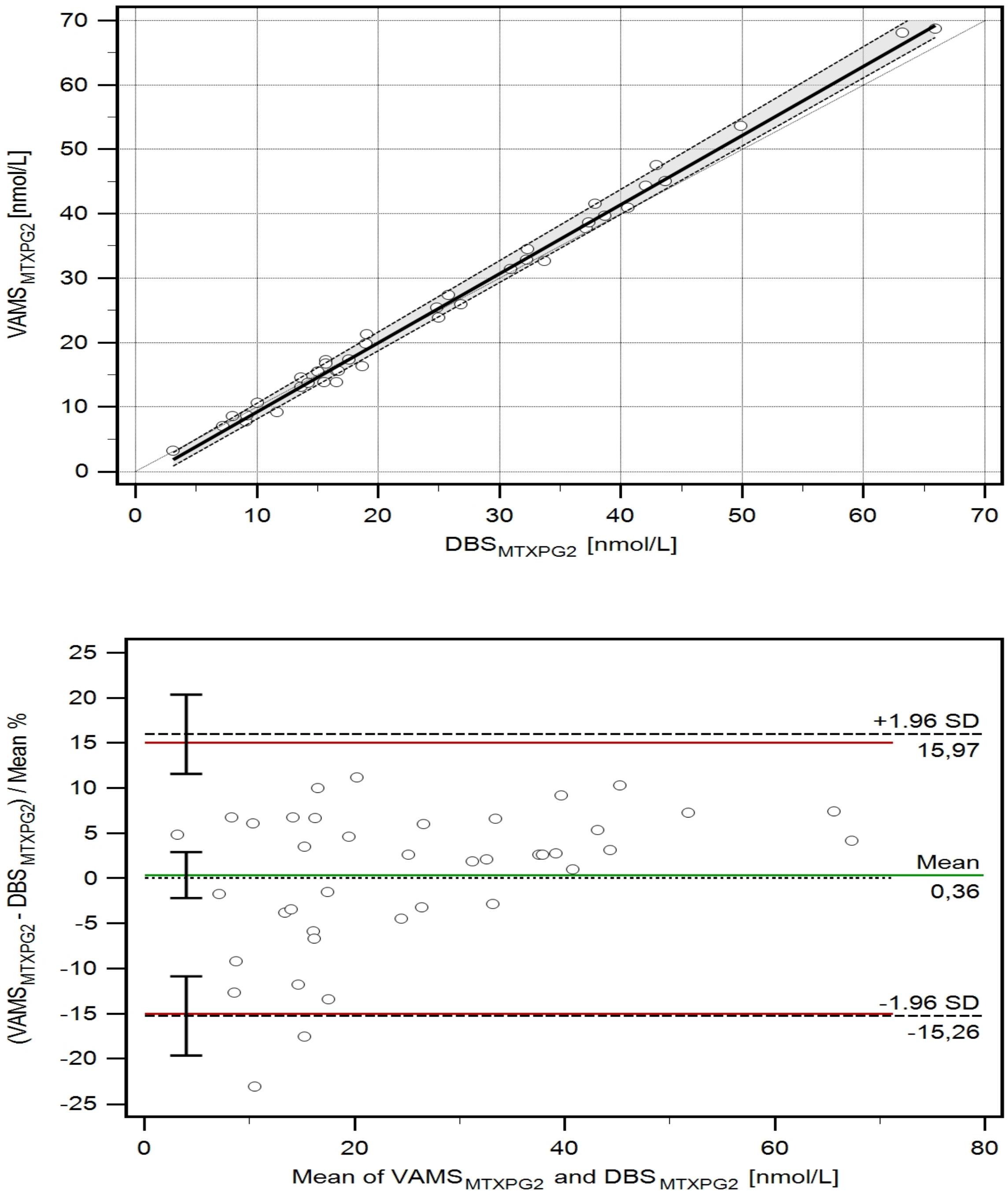

Fig. S2A–B. MTXPG2: PB regression (A) and BA analysis (B), DBS vs RBC.

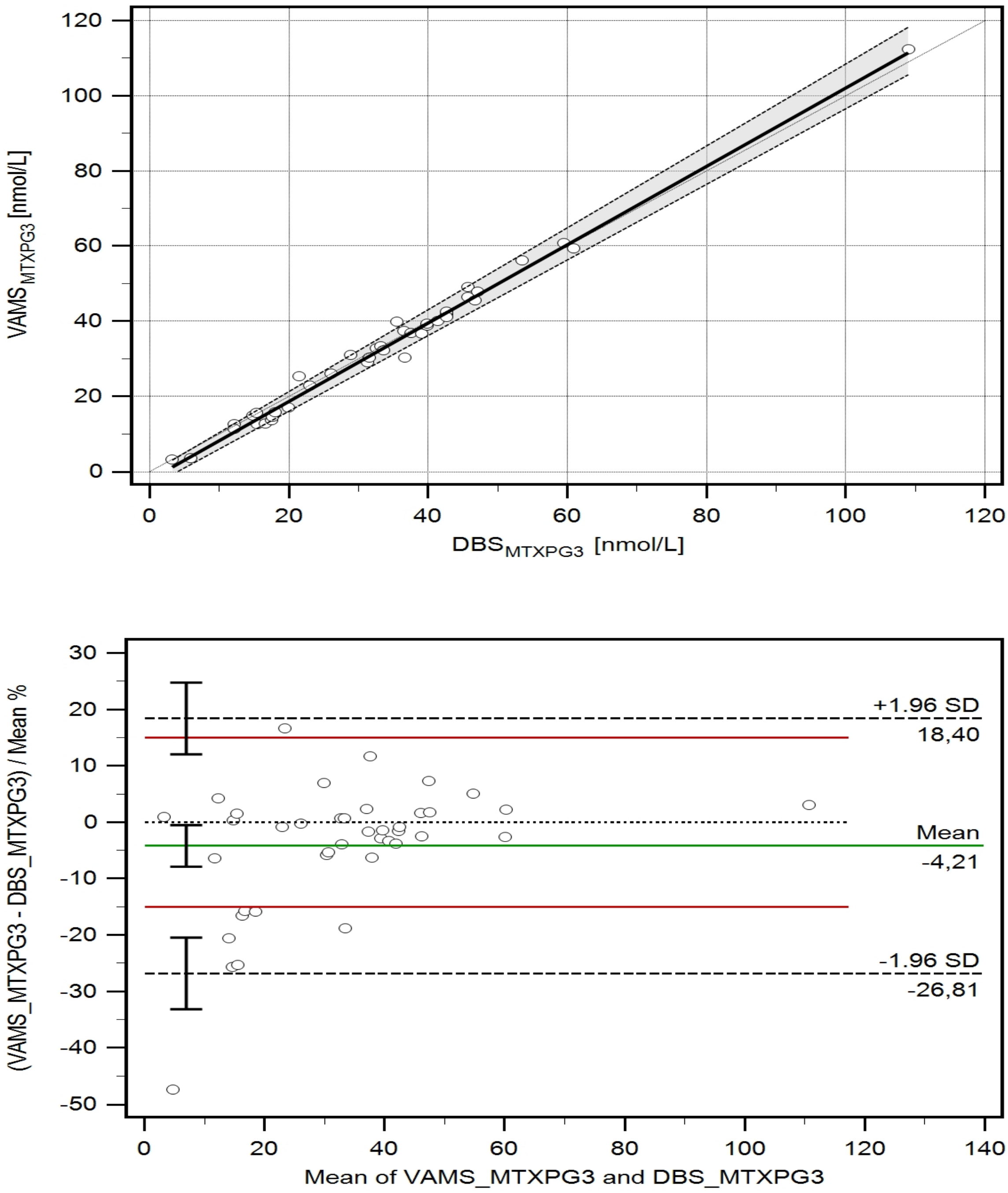

Fig. S3A–B. MTXPG3: PB regression (A) and BA analysis (B), DBS vs RBC.

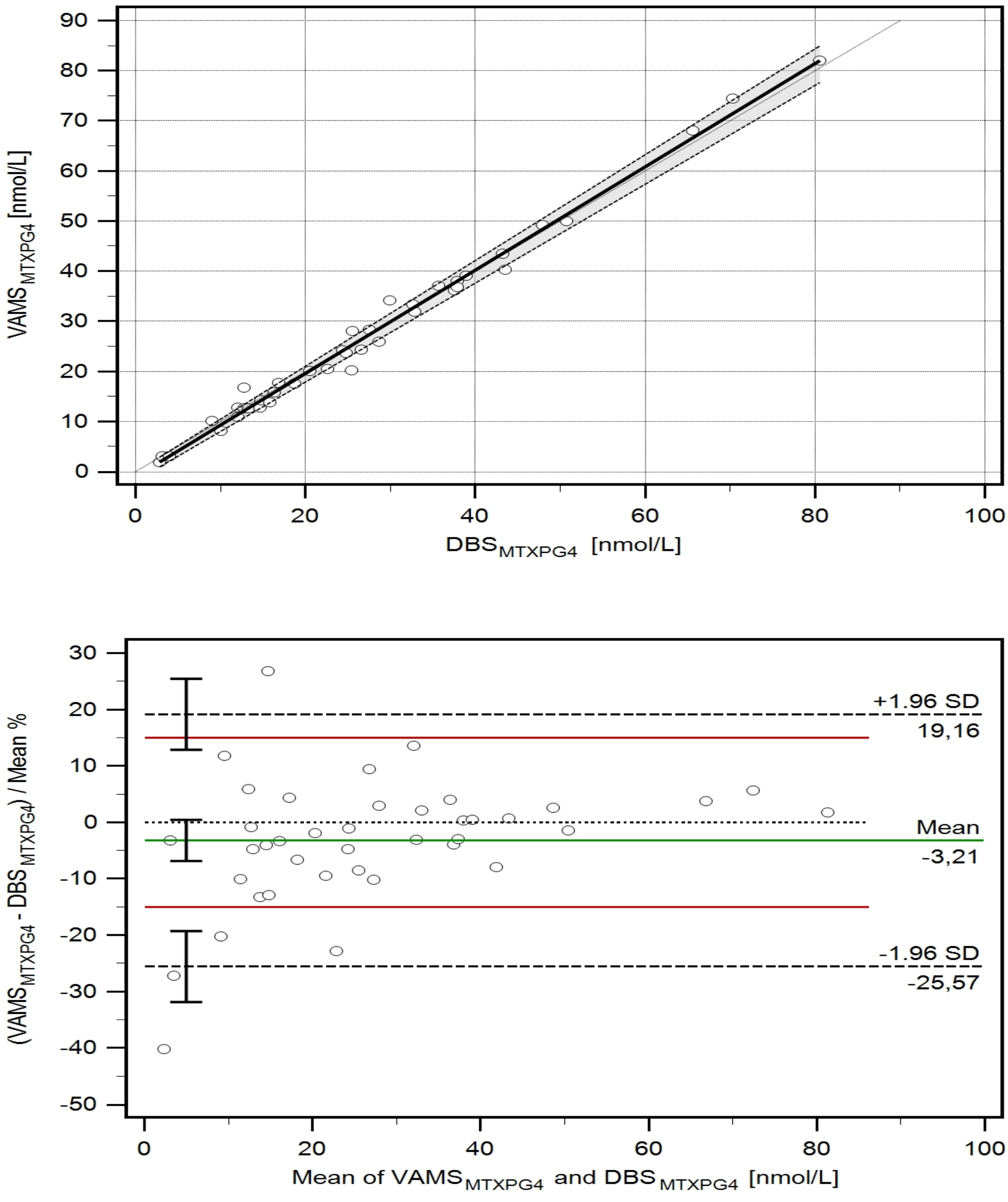

Fig. S4A–B. MTXPG4: PB regression (A) and BA analysis (B), DBS vs RBC.

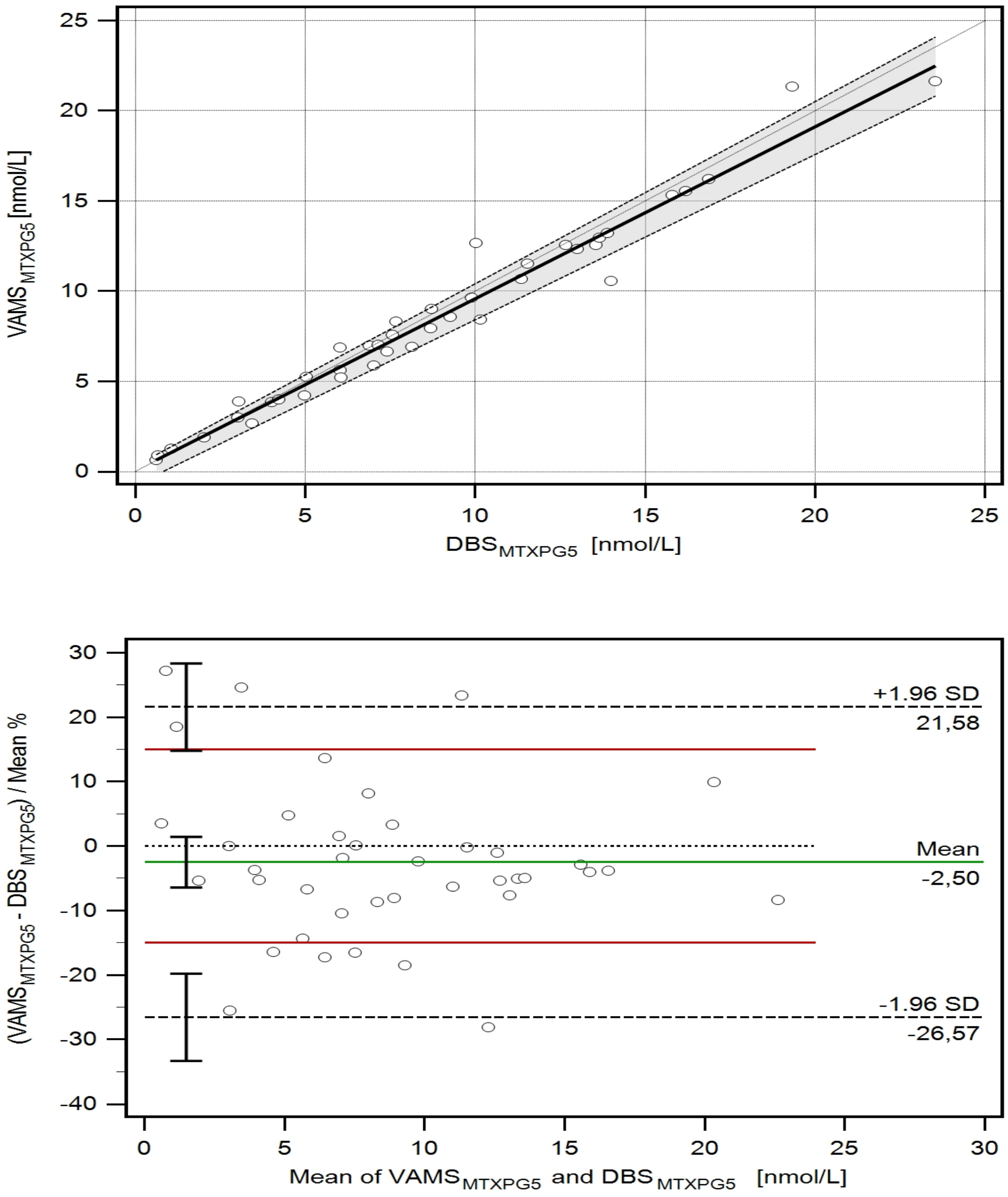

Fig. S5A–B. MTXPG5: PB regression (A) and BA analysis (B), DBS vs RBC.

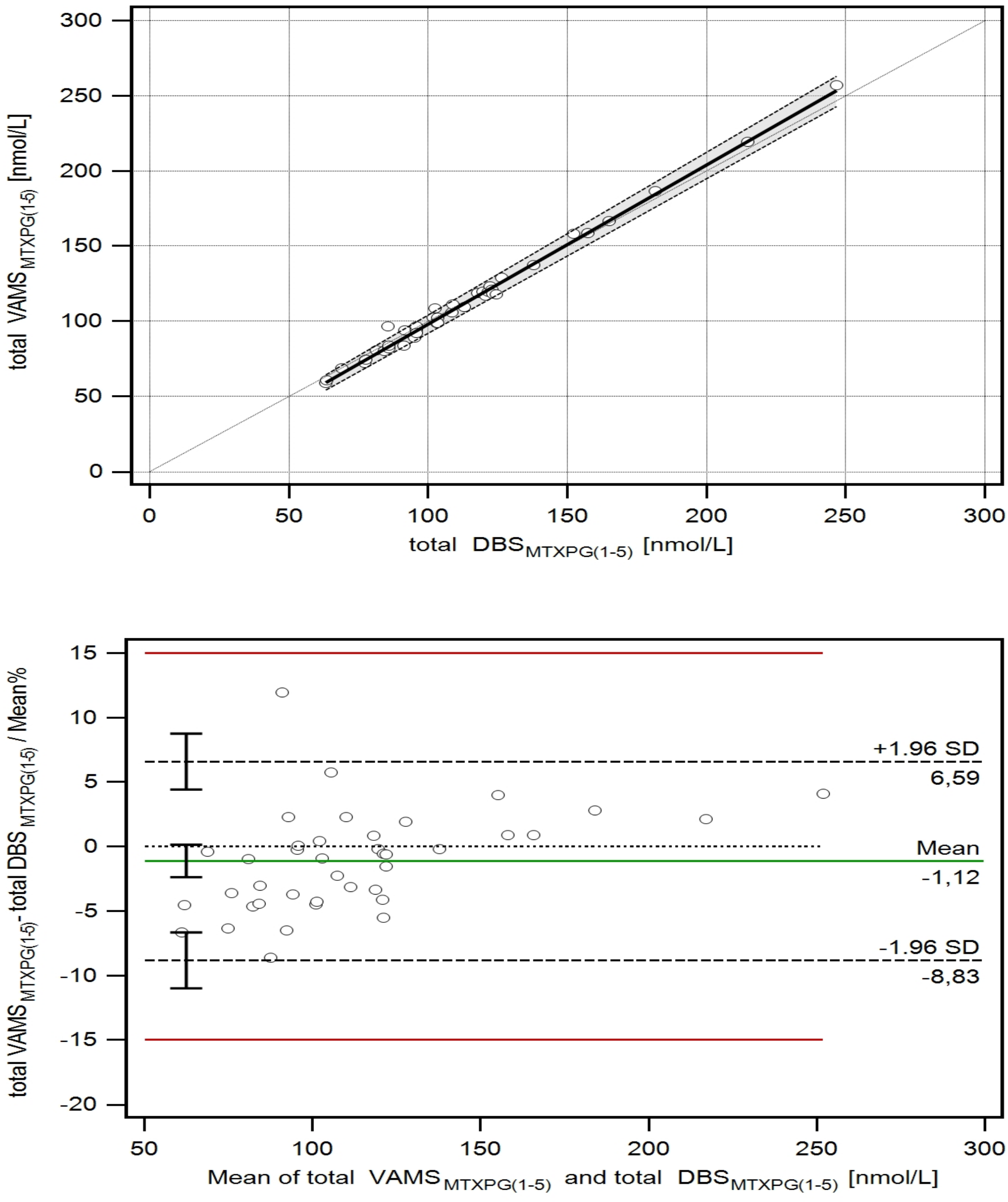

Fig. S6A–B. Total MTXPG (1–5): PB regression (A) and BA analysis (B), DBS vs RBC.

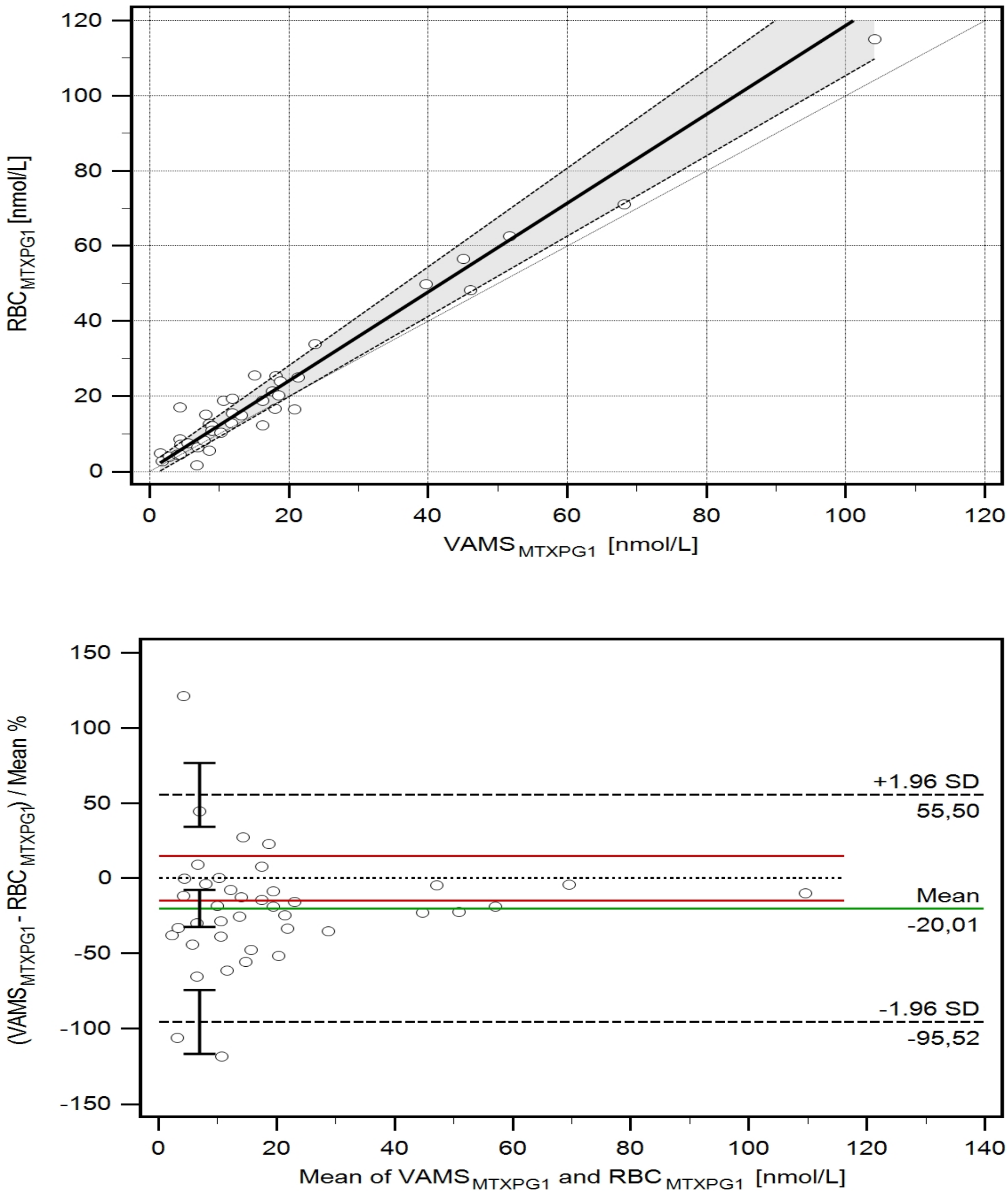

Fig. S7A–B. MTXPG1: PB regression (A) and BA analysis (B), DBS vs RBC.

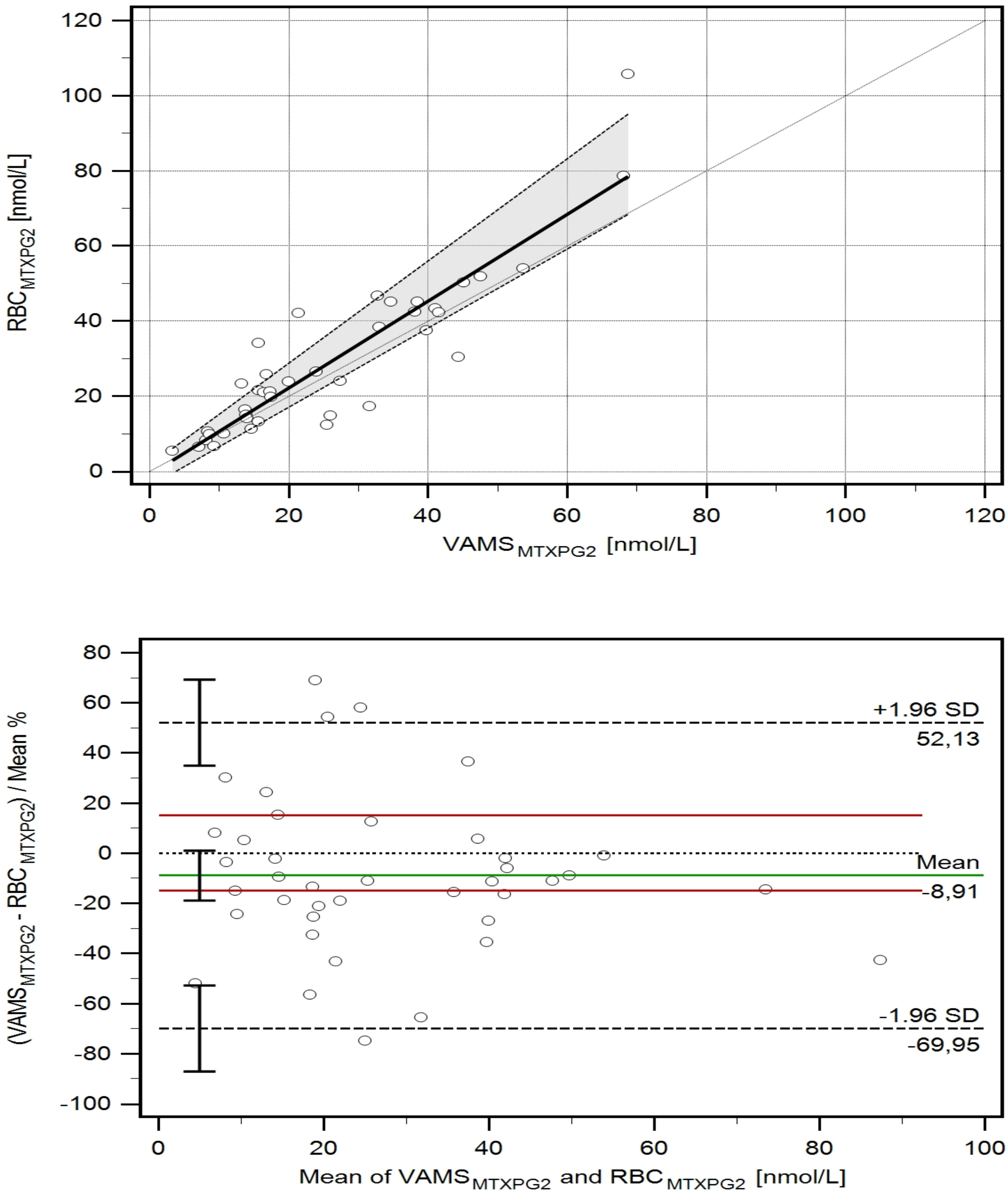

Fig. S8A–B. MTXPG2: PB regression (A) and BA analysis (B), DBS vs RBC.

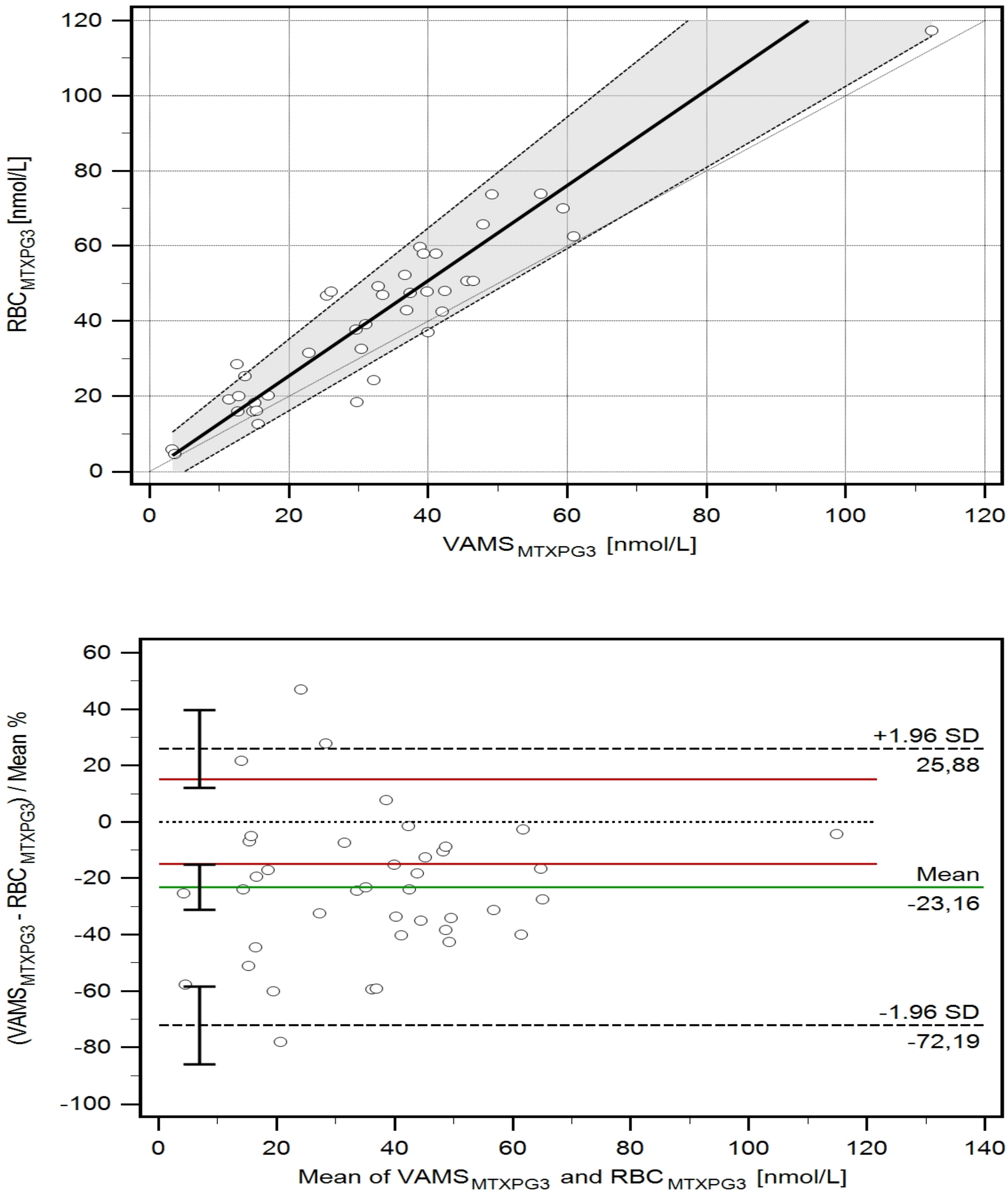

Fig. S9A–B. MTXPG3: PB regression (A) and BA analysis (B), DBS vs RBC.

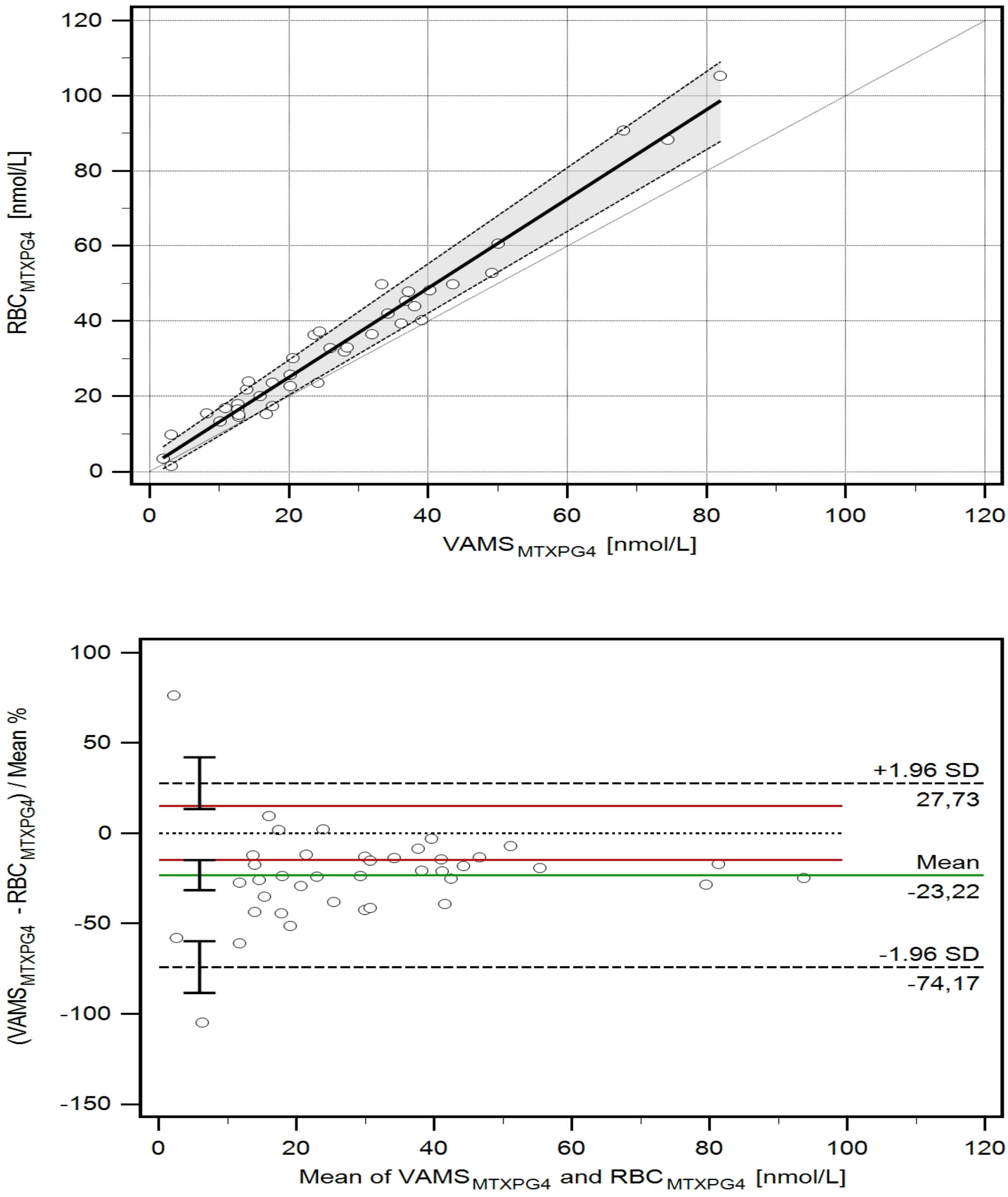

Fig. S10A–B. MTXPG4: PB regression (A) and BA analysis (B), DBS vs RBC.

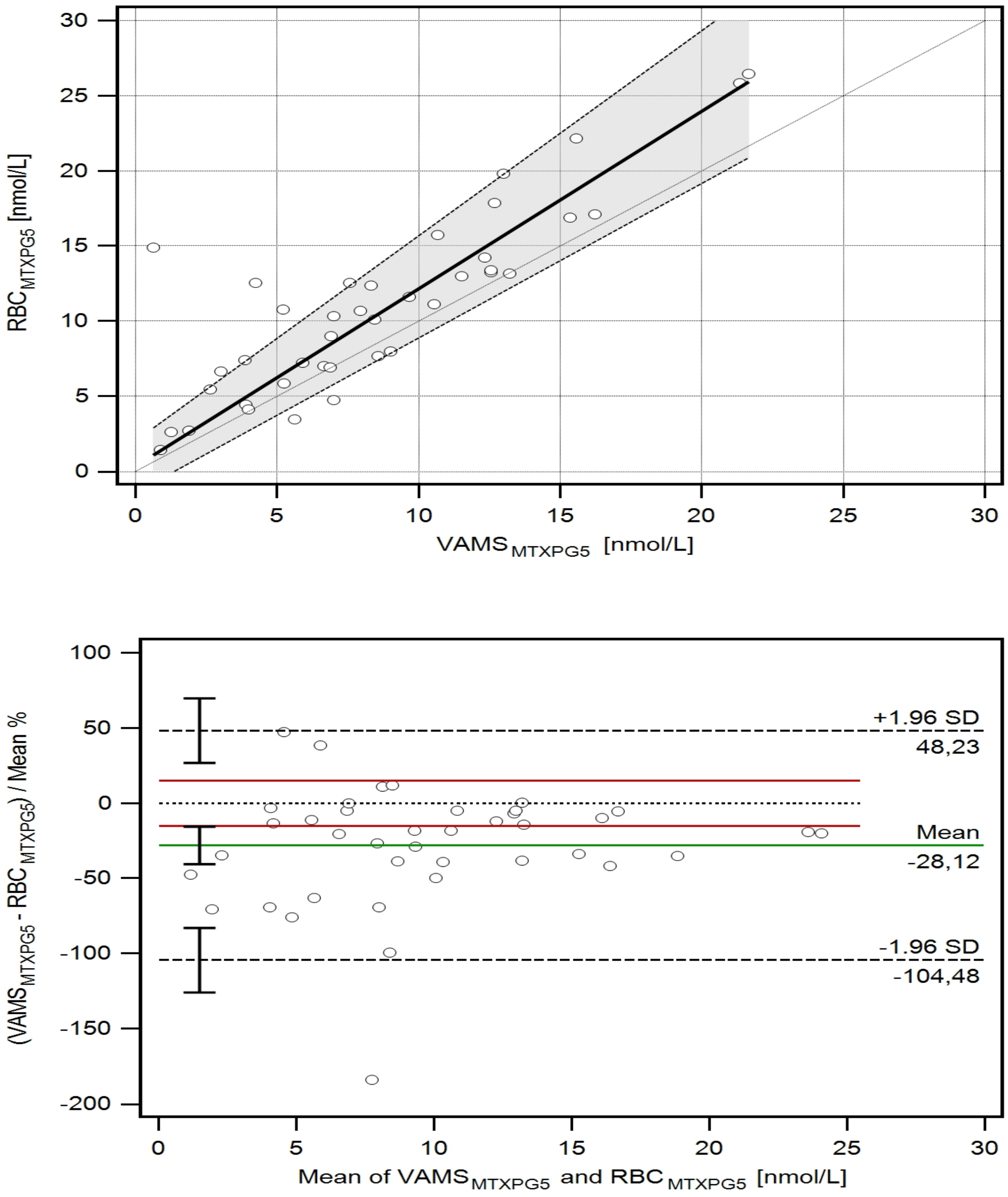

Fig. S11A–B. MTXPG5: PB regression (A) and BA analysis (B), DBS vs RBC.

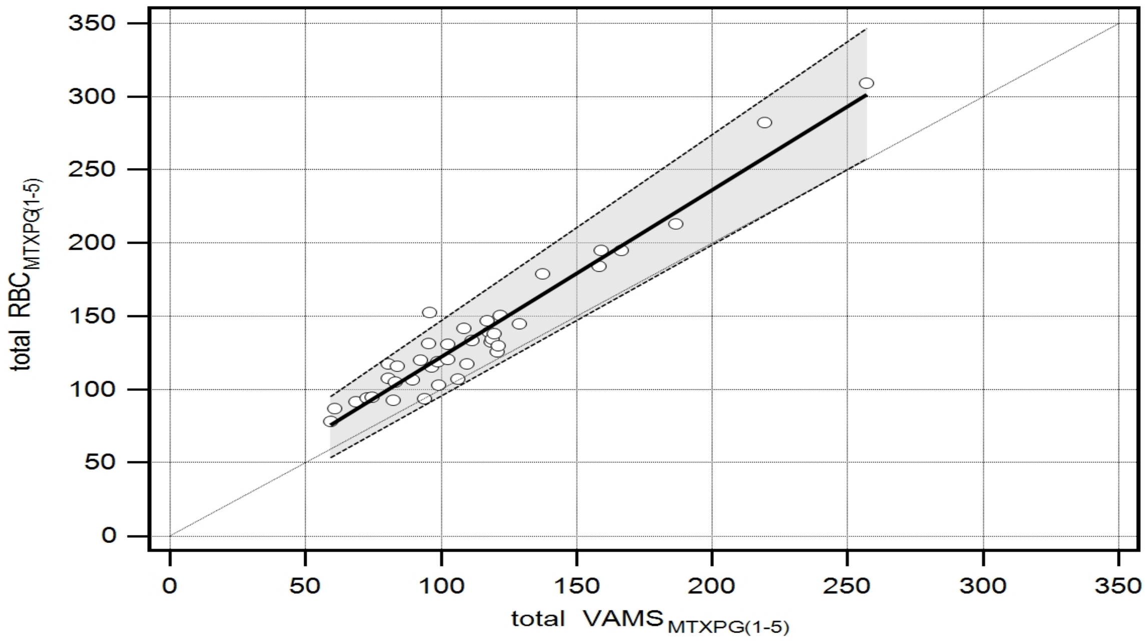

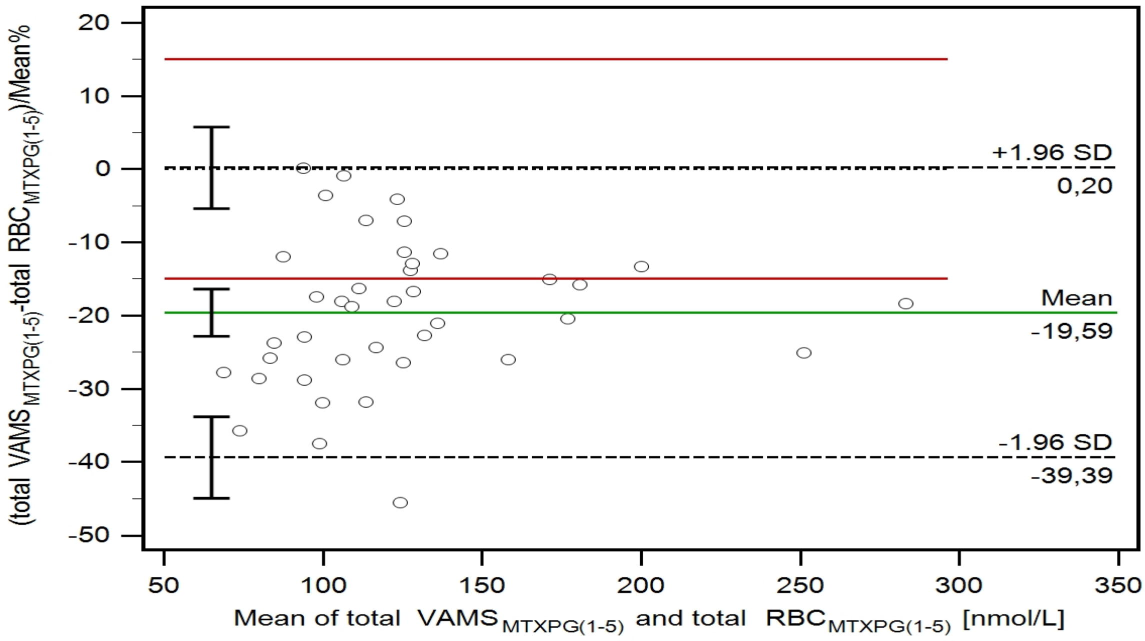

Fig. S12A–B. Total MTXPG (1–5): PB regression (A) and BA analysis (B), DBS vs RBC.

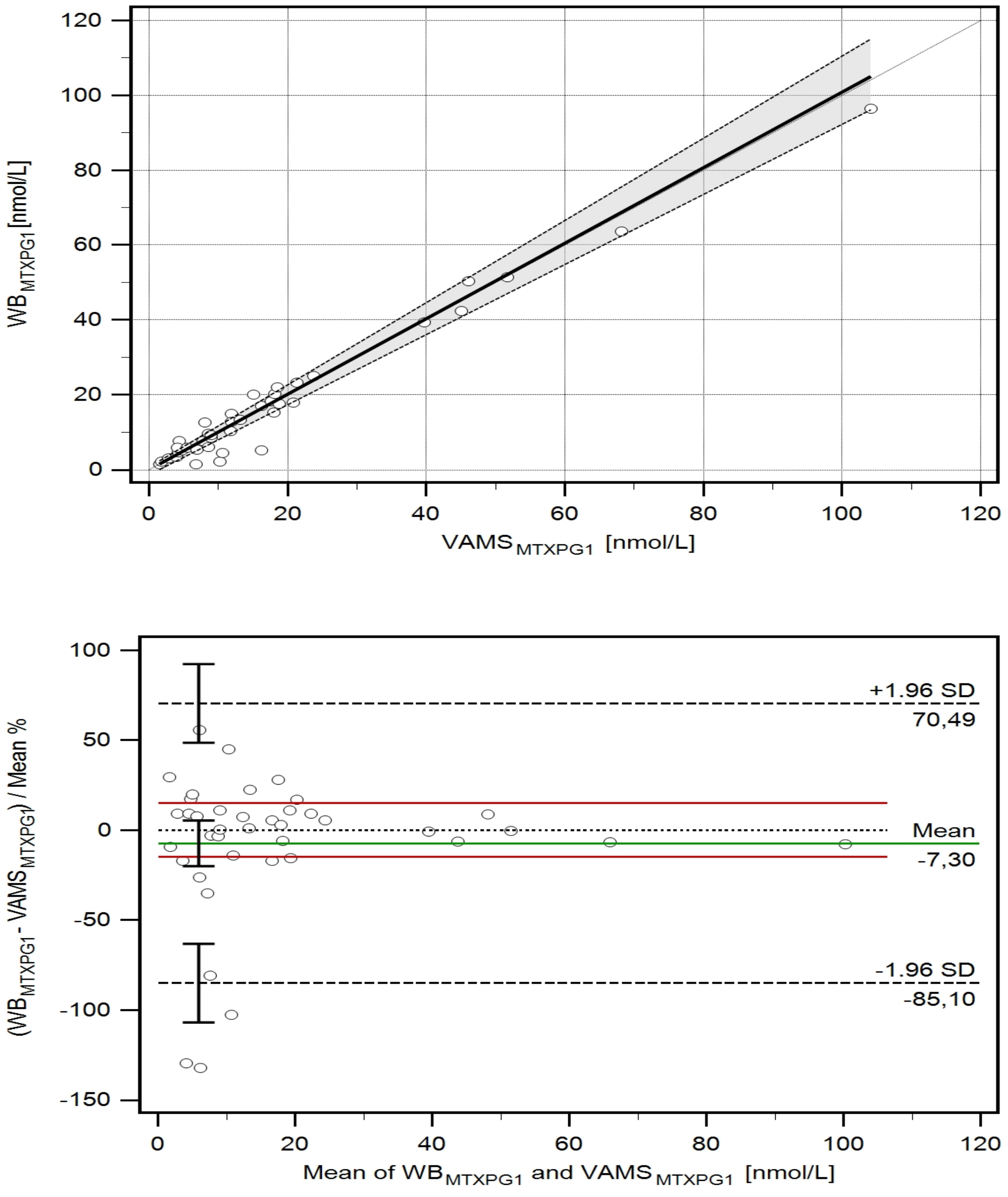

Fig. S13A–B. MTXPG1: PB regression (A) and BA analysis (B), DBS vs WB.

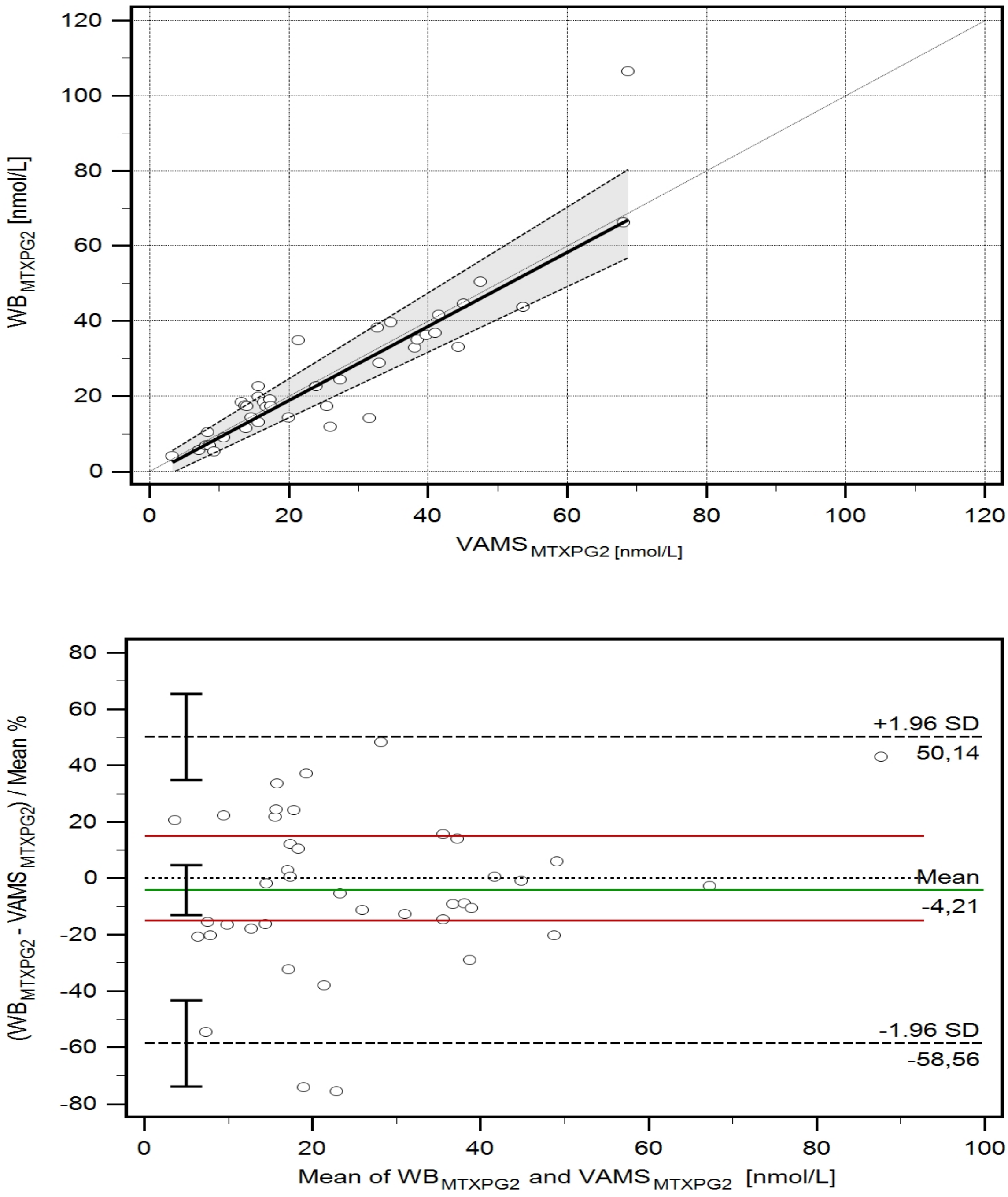

Fig. S14A–B. MTXPG2: PB regression (A) and BA analysis (B), DBS vs WB.

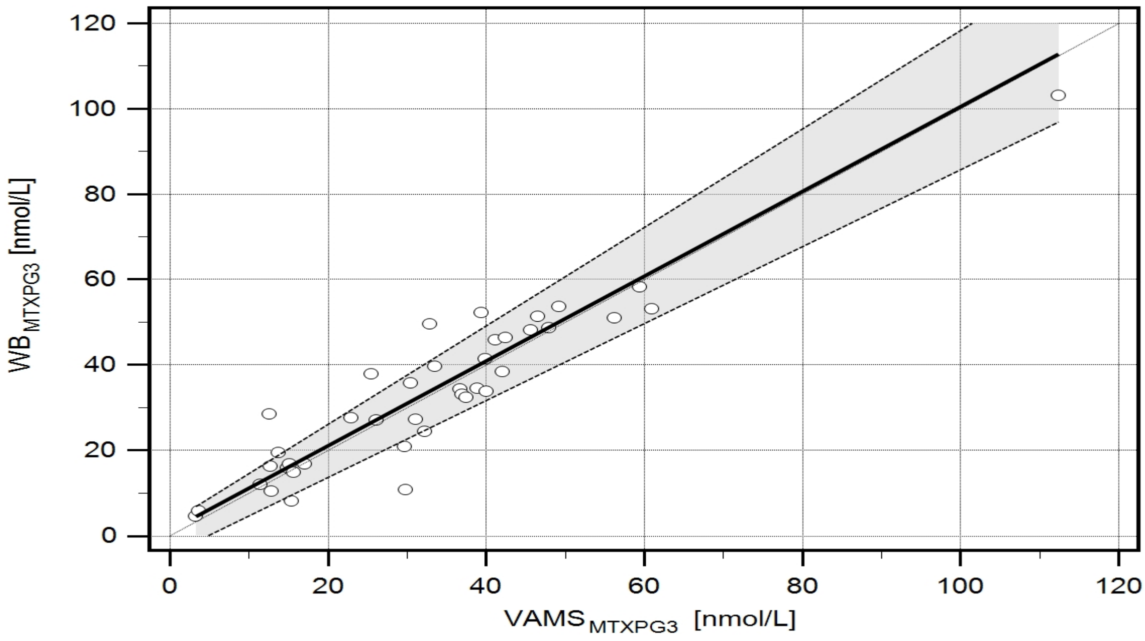

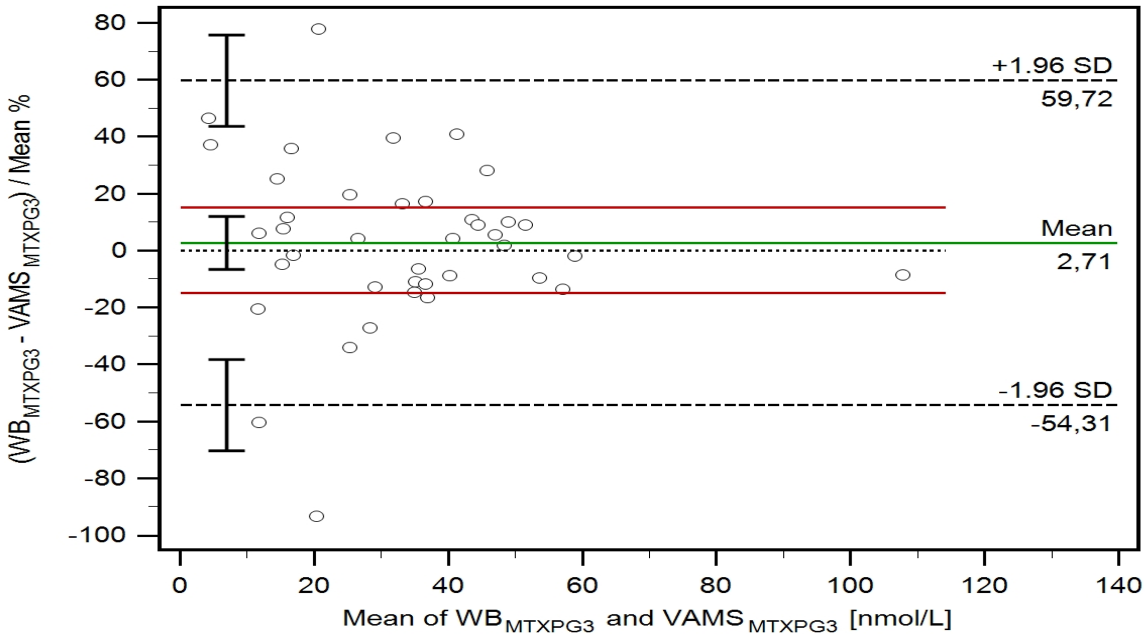

Fig. S15A–B. MTXPG3: PB regression (A) and BA analysis (B), DBS vs WB.

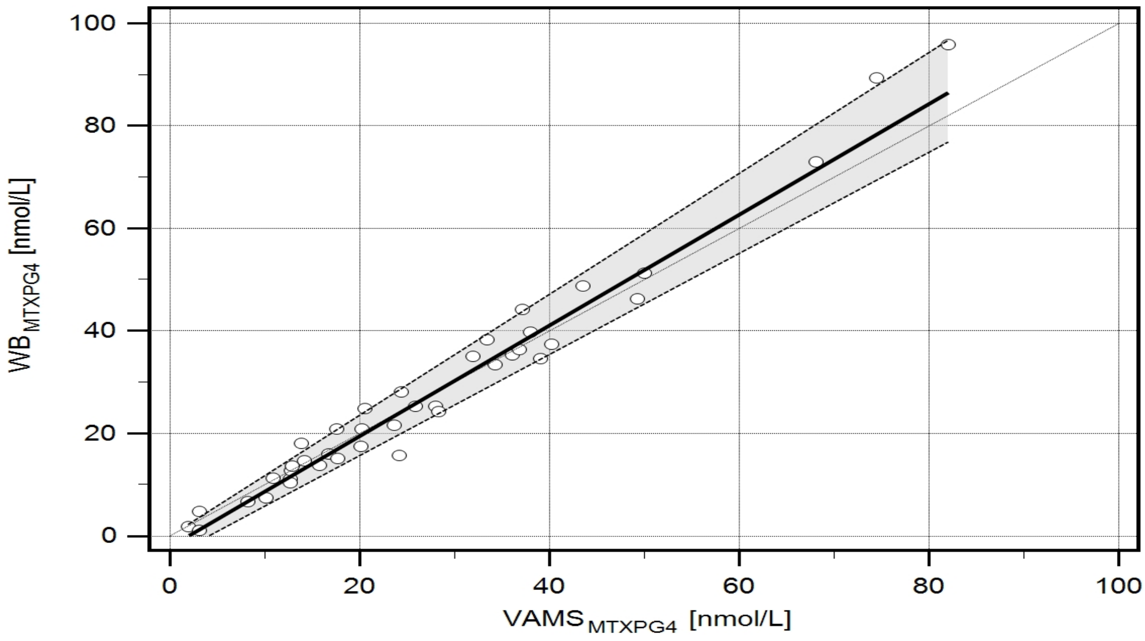

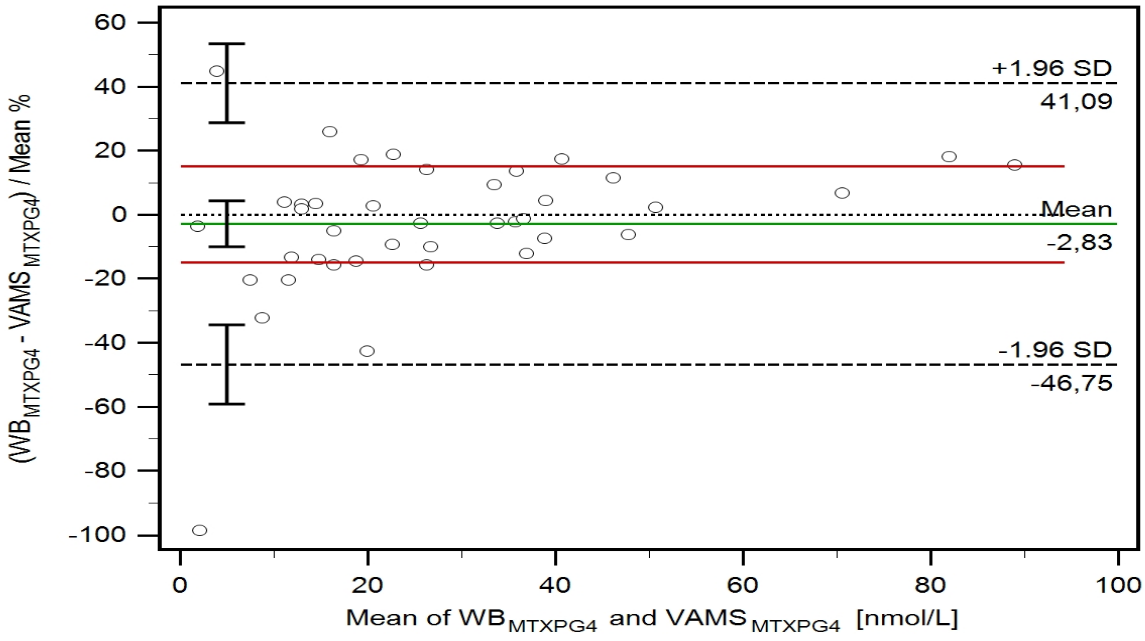

Fig. S16A–B. MTXPG4: PB regression (A) and BA analysis (B), DBS vs WB.

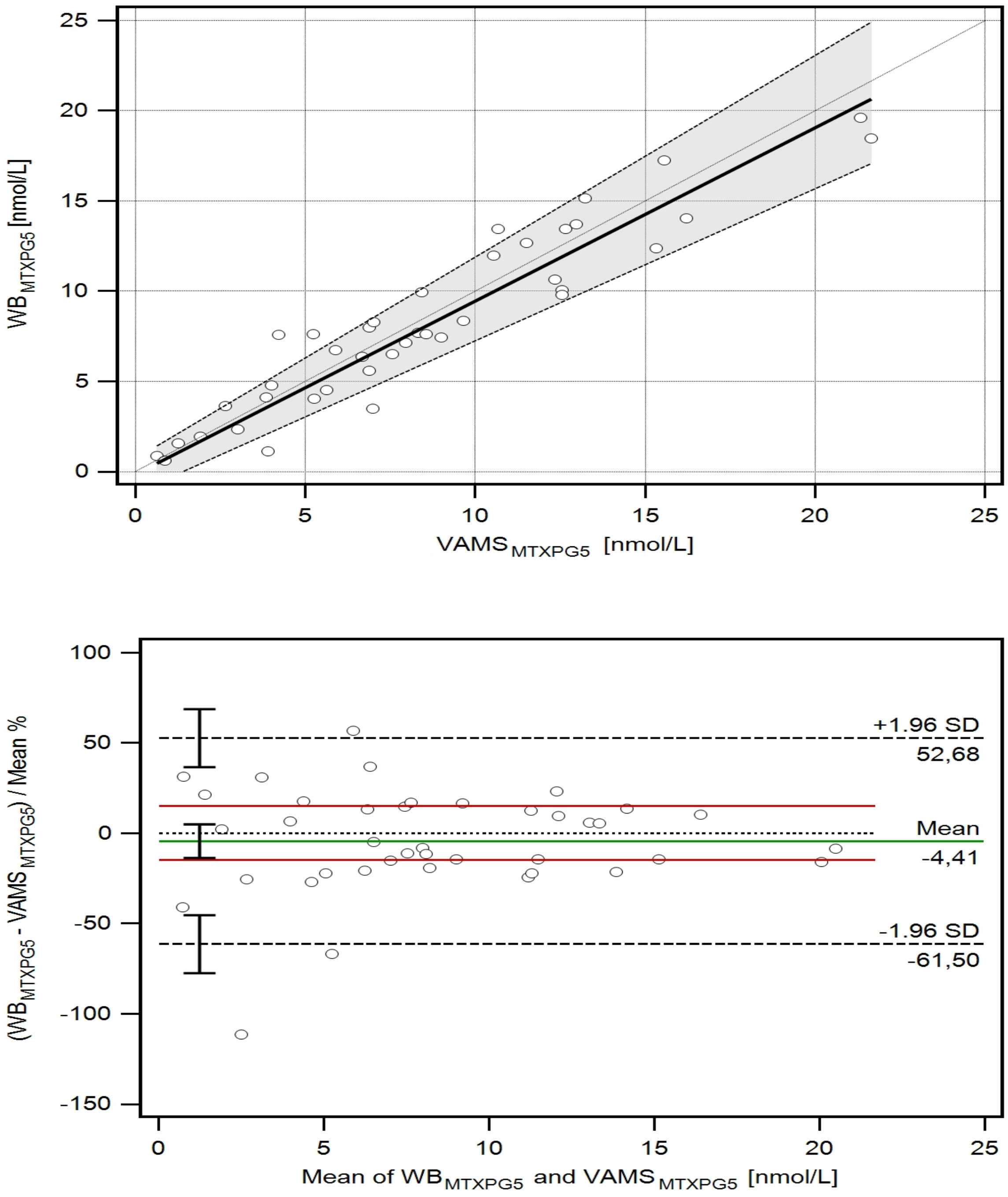

Fig. S17A–B. MTXPG5: PB regression (A) and BA analysis (B), DBS vs WB.

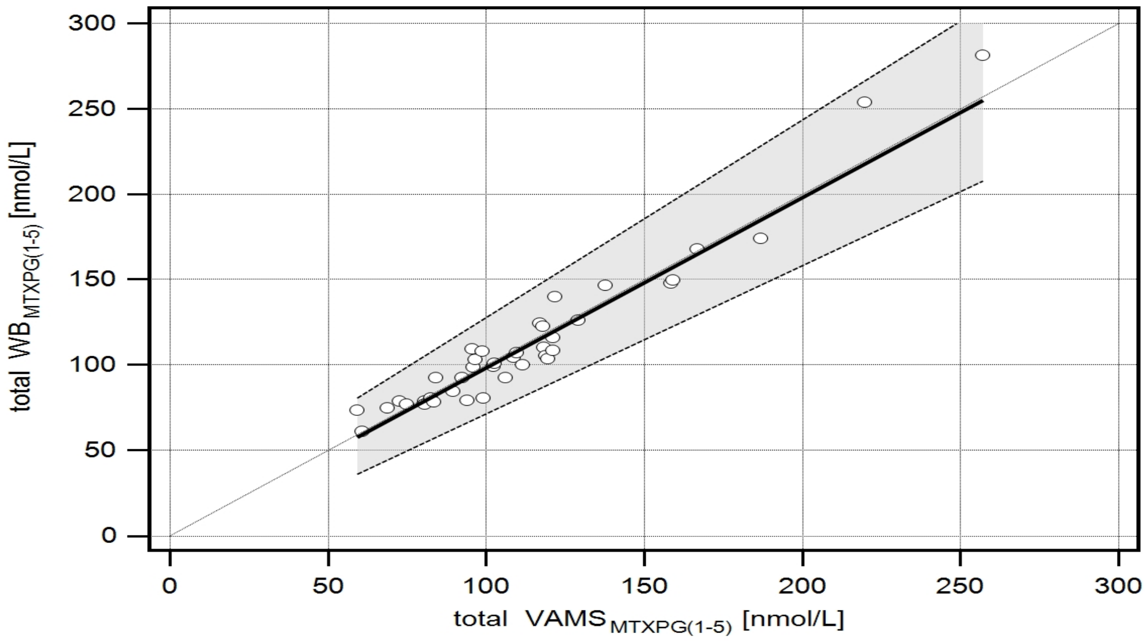

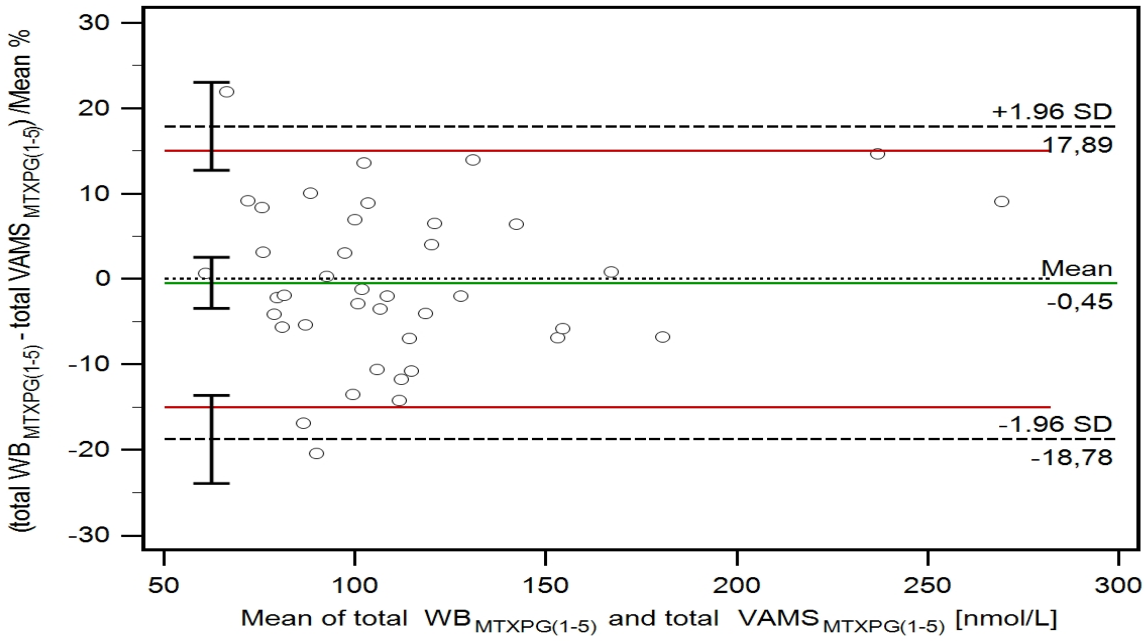

Fig. S18A–B. Total MTXPG (1–5): PB regression (A) and BA analysis (B), DBS vs WB.

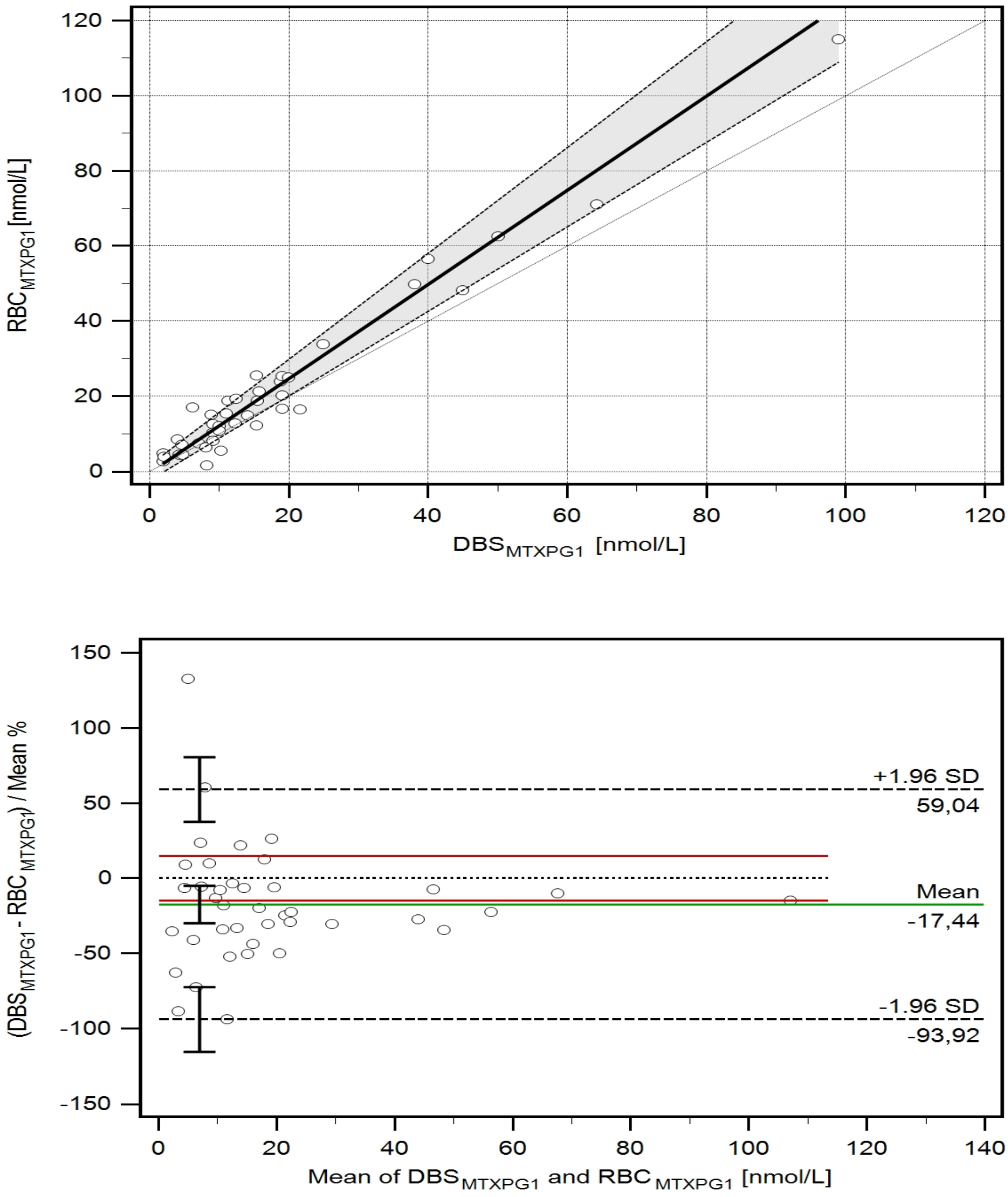

Fig. S19A–B. MTXPG1: PB regression (A) and BA analysis (B), DBS vs WB.

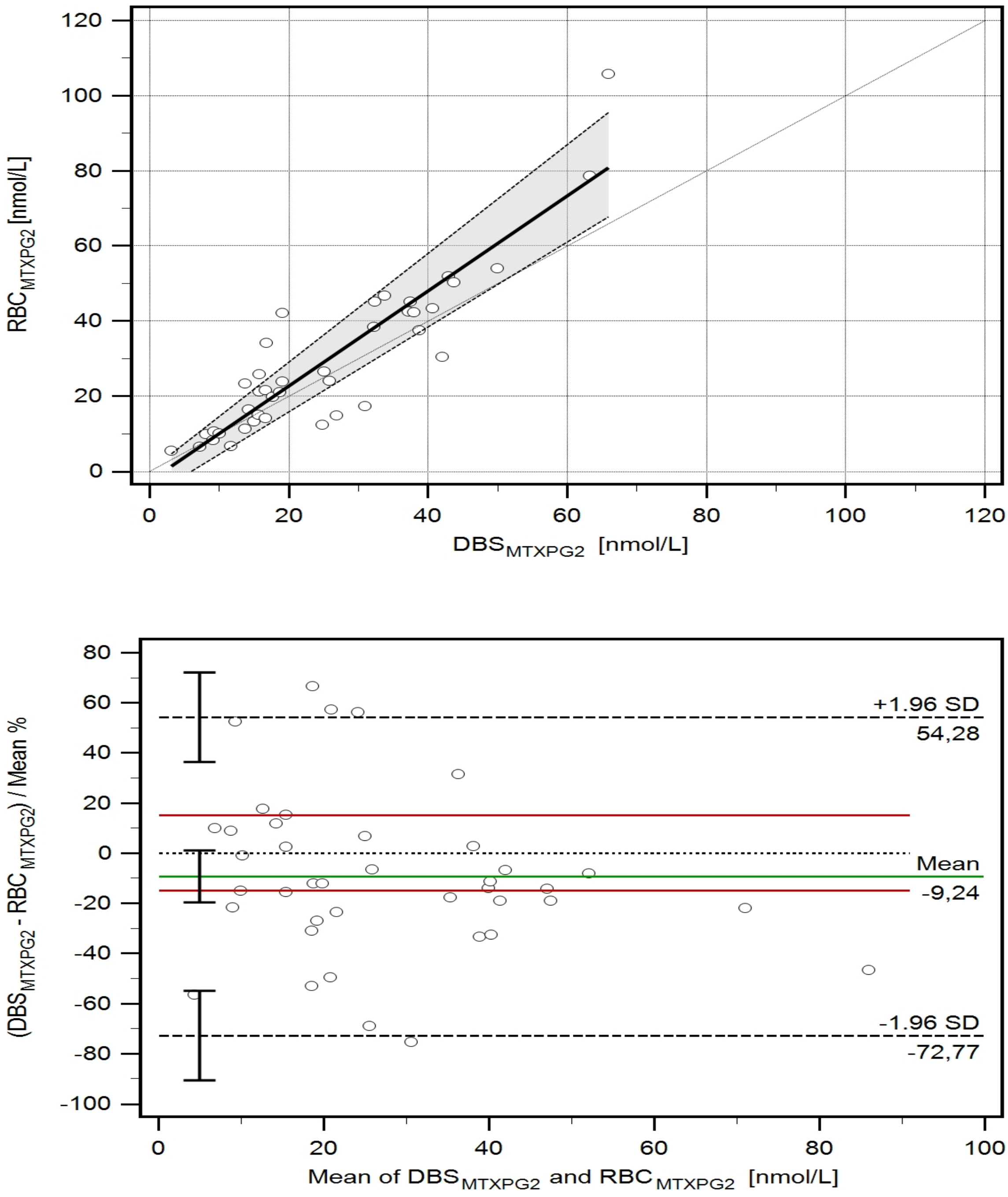

Fig. S20A–B. MTXPG2: PB regression (A) and BA analysis (B), DBS vs WB.

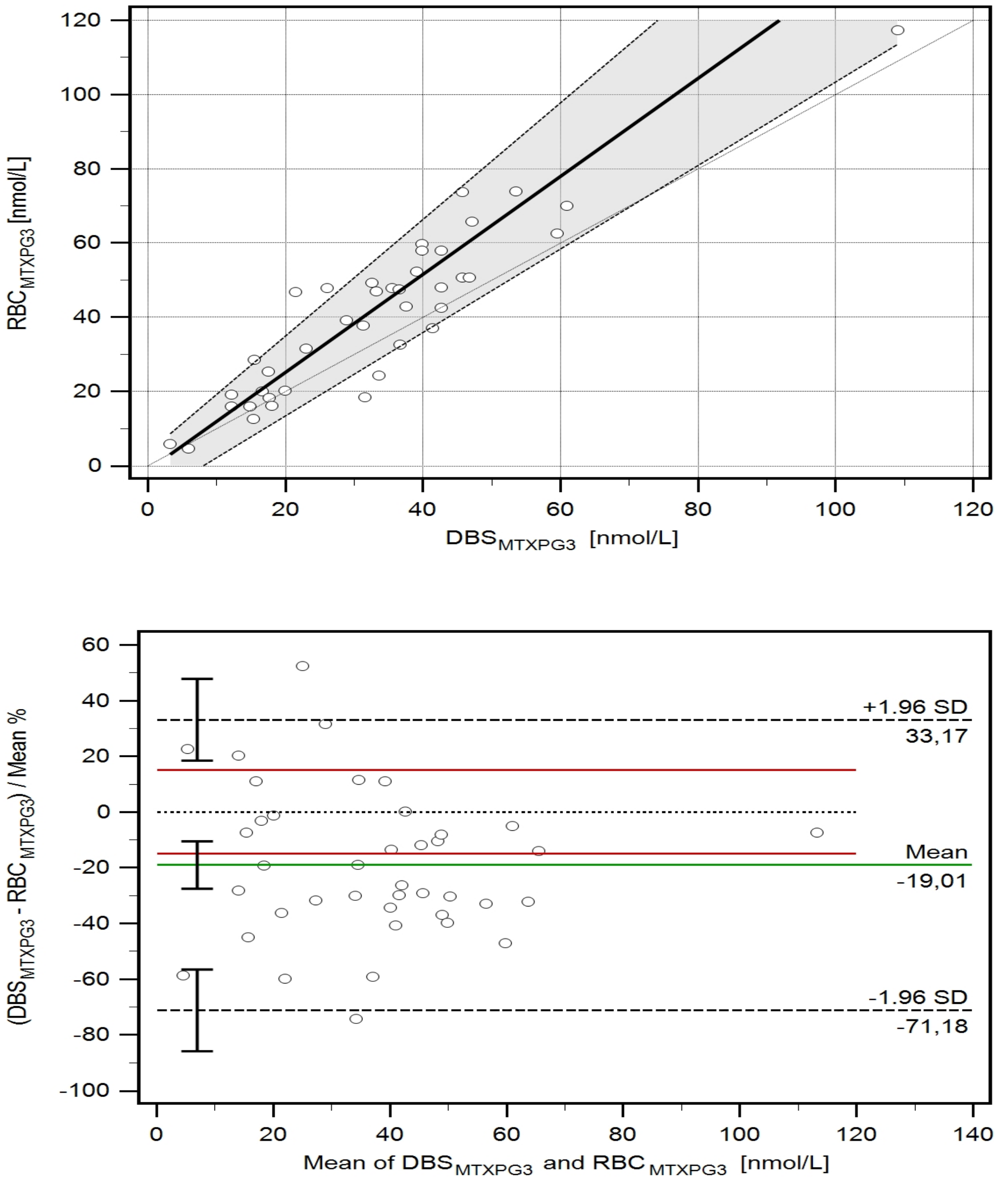

Fig. S21A–B. MTXPG3: PB regression (A) and BA analysis (B), DBS vs WB.

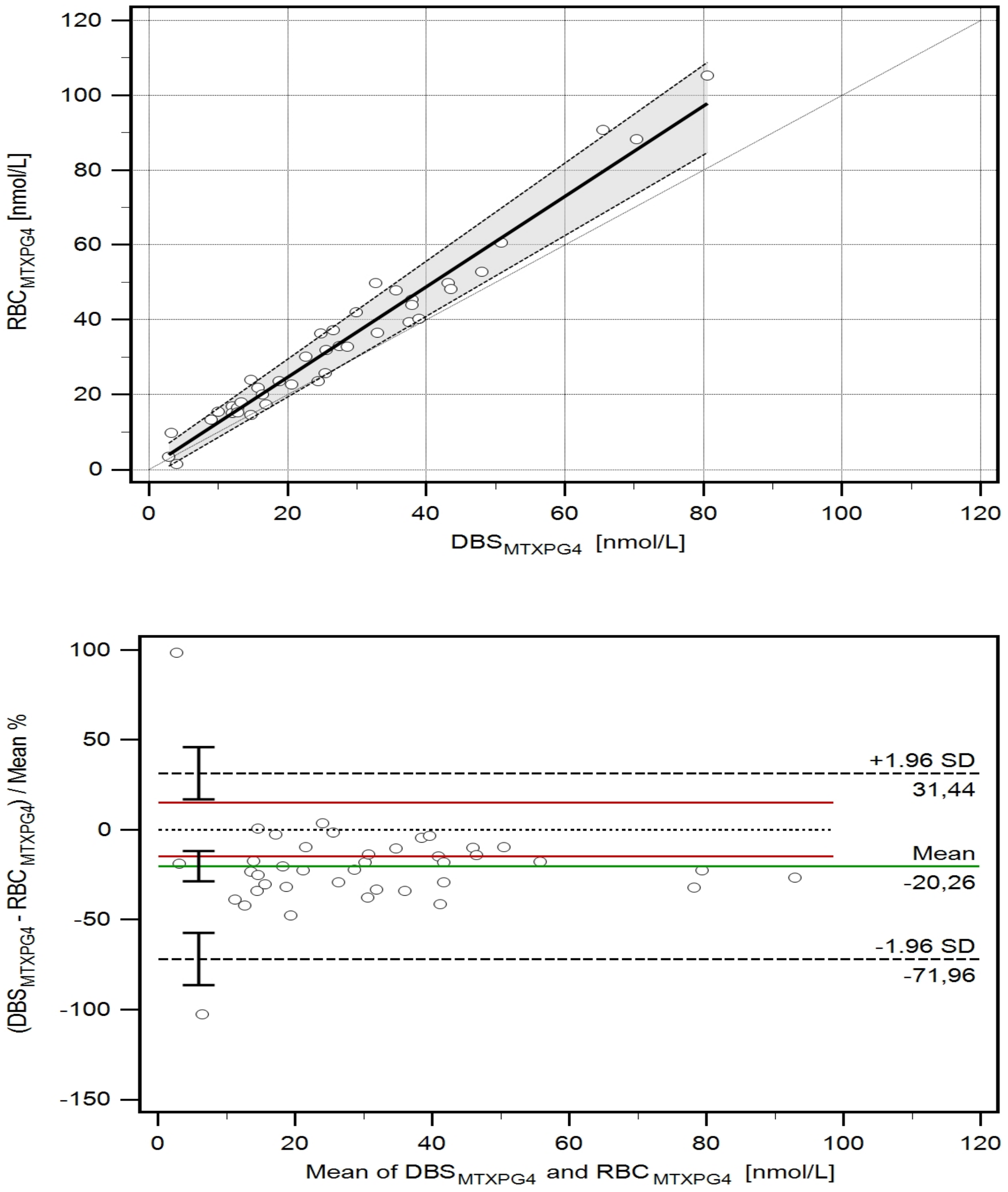

Fig. S22A–B. MTXPG4: PB regression (A) and BA analysis (B), DBS vs WB.

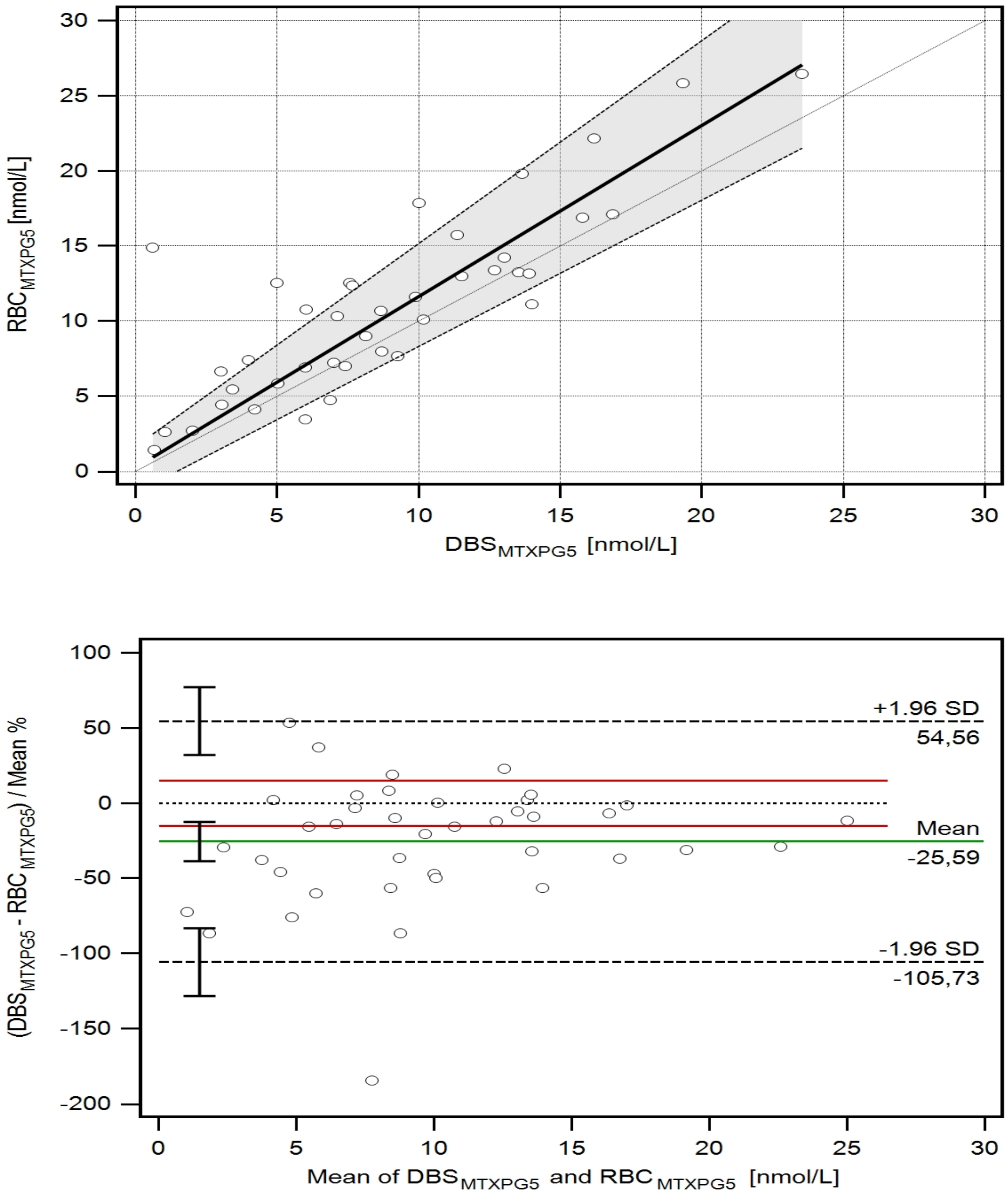

Fig. S23A–B. MTXPG5: PB regression (A) and BA analysis (B), DBS vs WB.

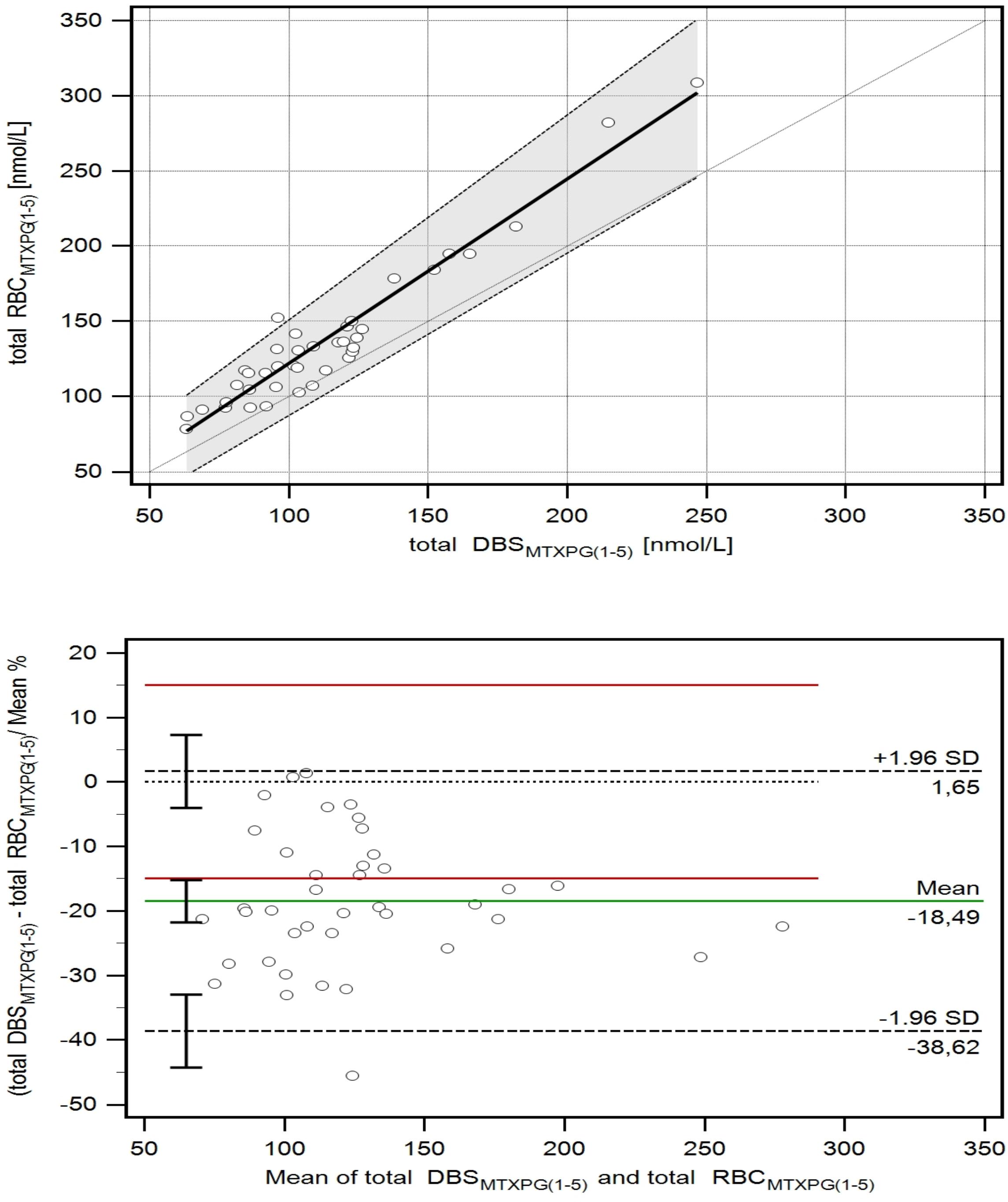

Fig. S24A–B. Total MTXPG (1–5): PB regression (A) and BA analysis (B), DBS vs WB.

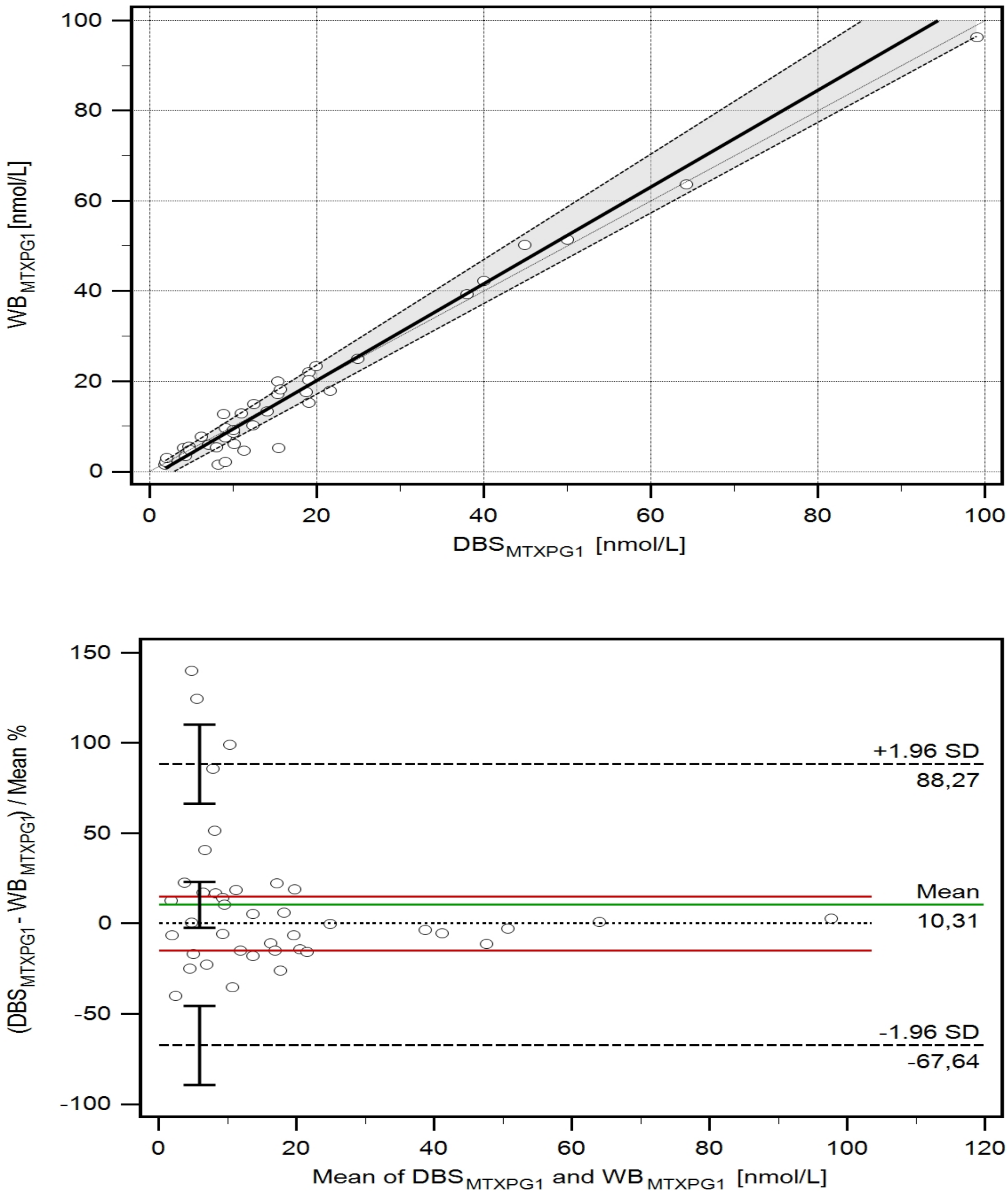

Fig. S25A–B. MTXPG1: PB regression (A) and BA analysis (B), RBC vs WB.

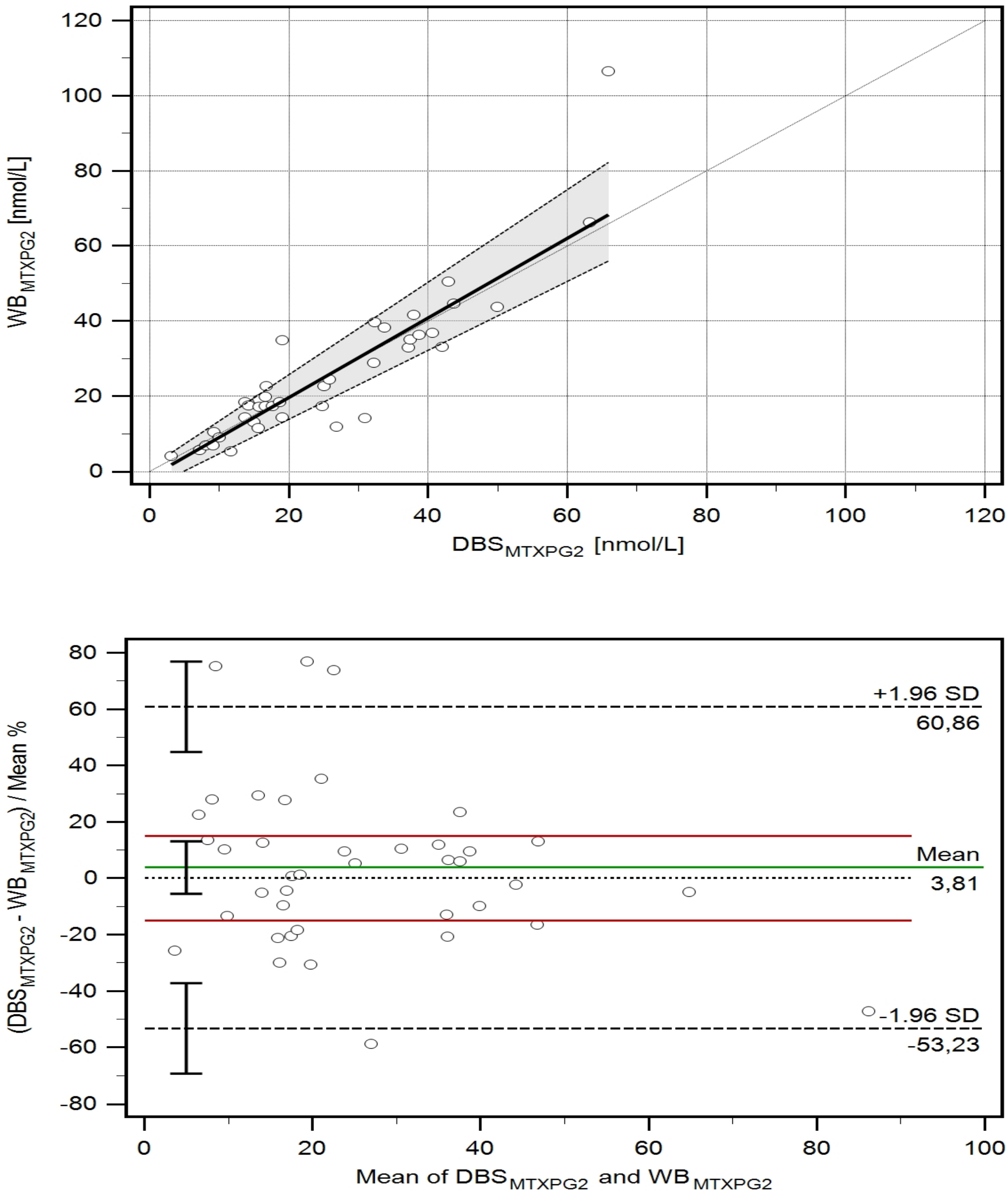

Fig. S26A–B. MTXPG2: PB regression (A) and BA analysis (B), RBC vs WB.

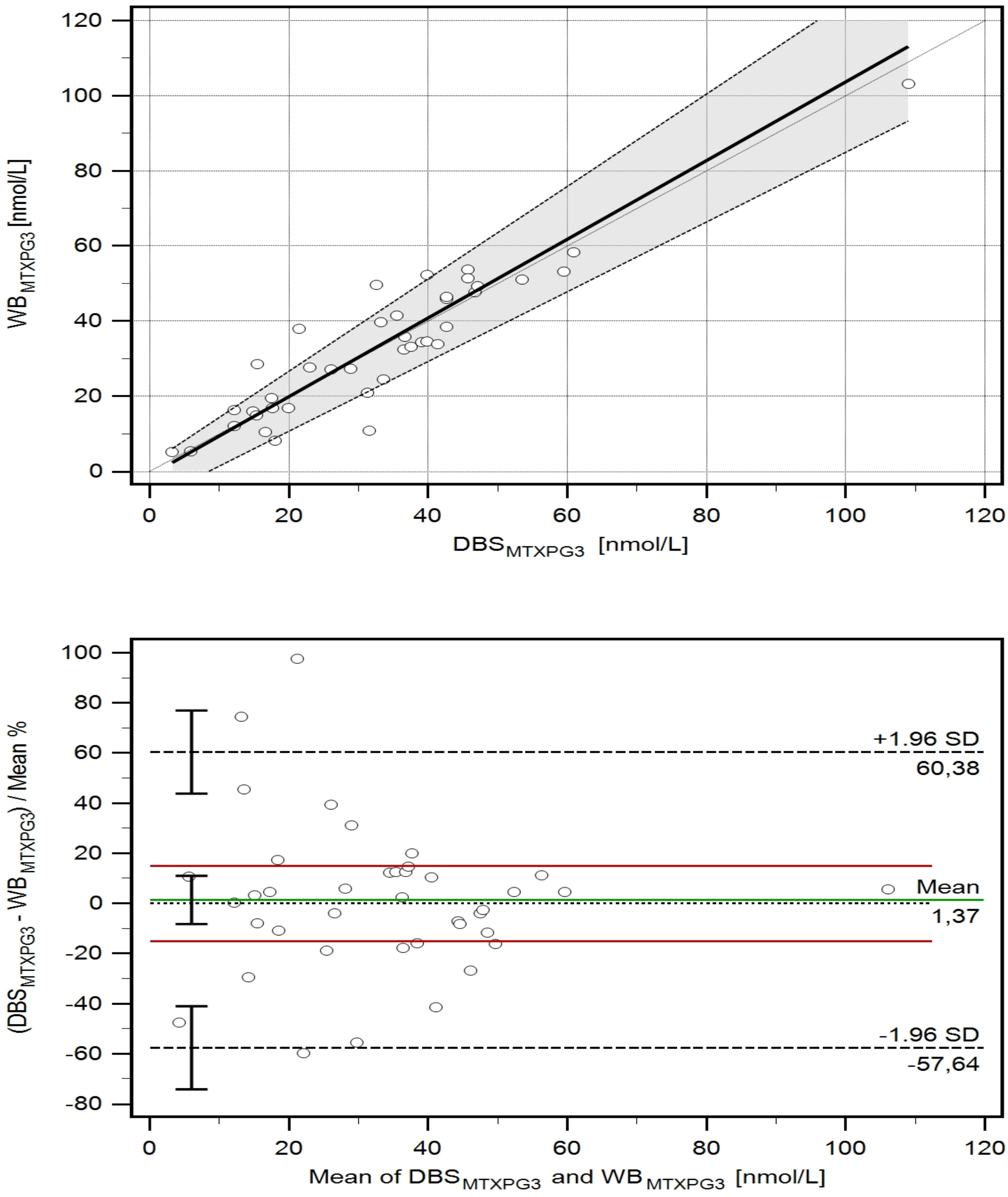

Fig. S27A–B. MTXPG3: PB regression (A) and BA analysis (B), RBC vs WB.

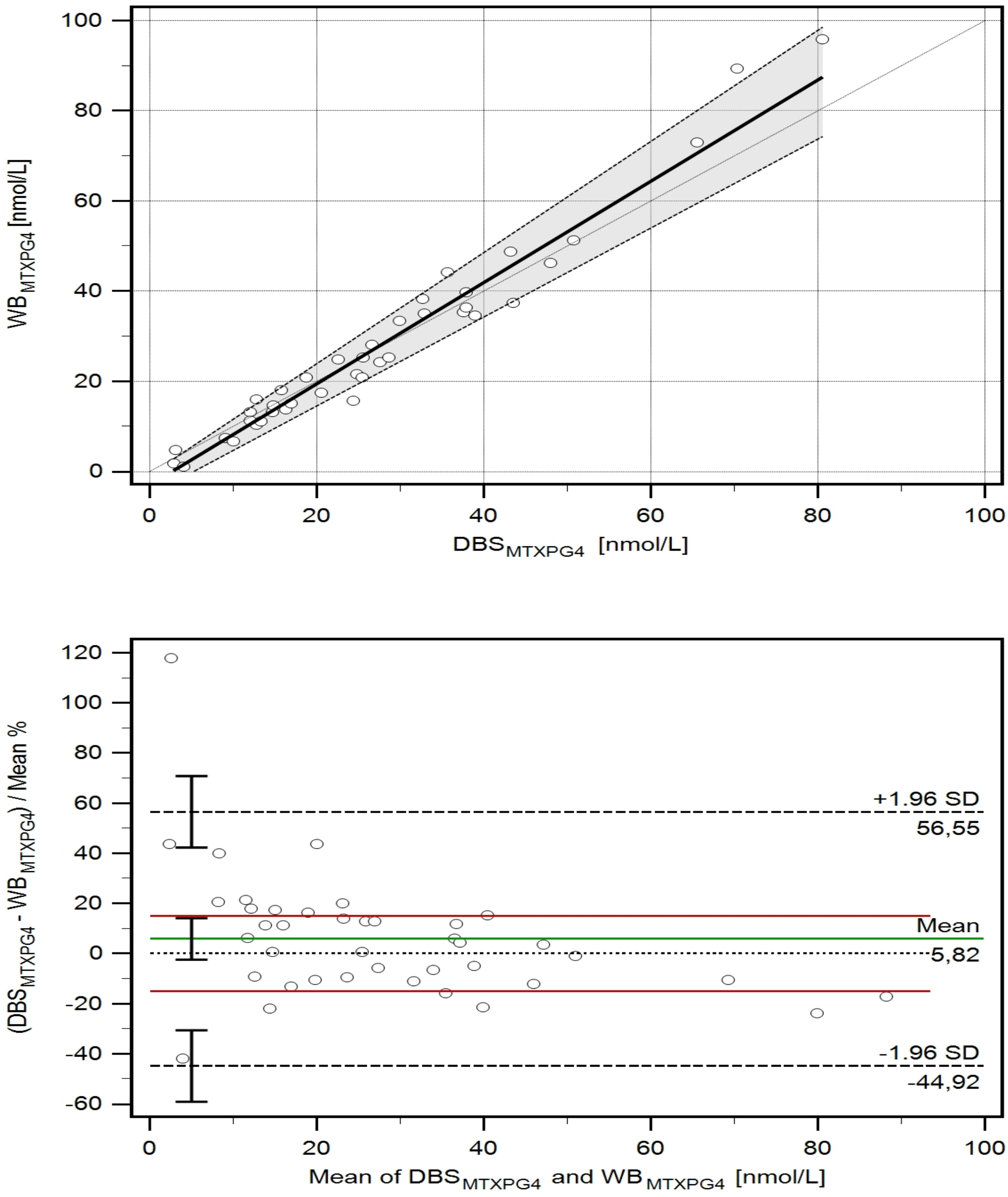

Fig. S28A–B. MTXPG4: PB regression (A) and BA analysis (B), RBC vs WB.

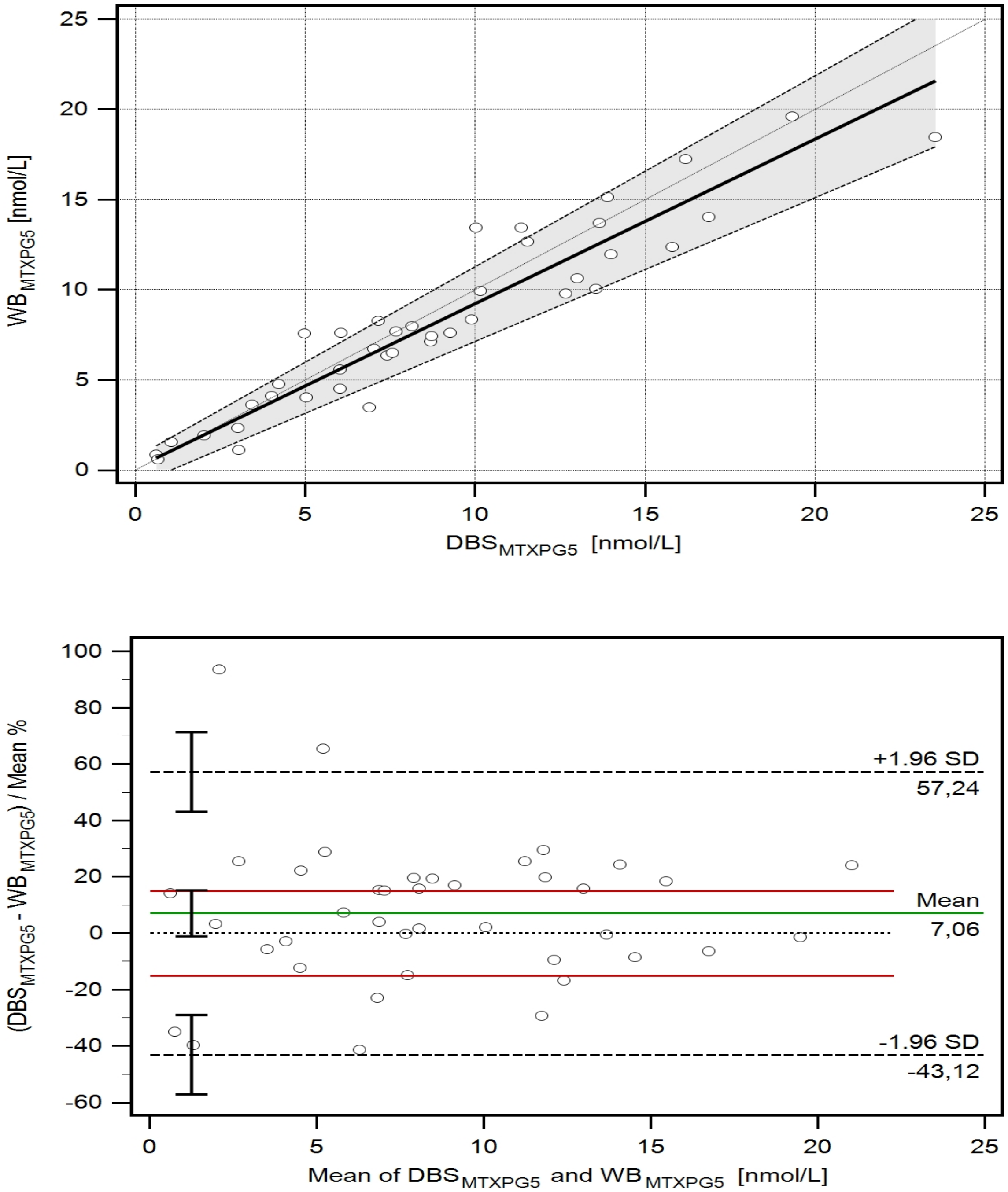

Fig. S29A–B. MTXPG5: PB regression (A) and BA analysis (B), RBC vs WB.

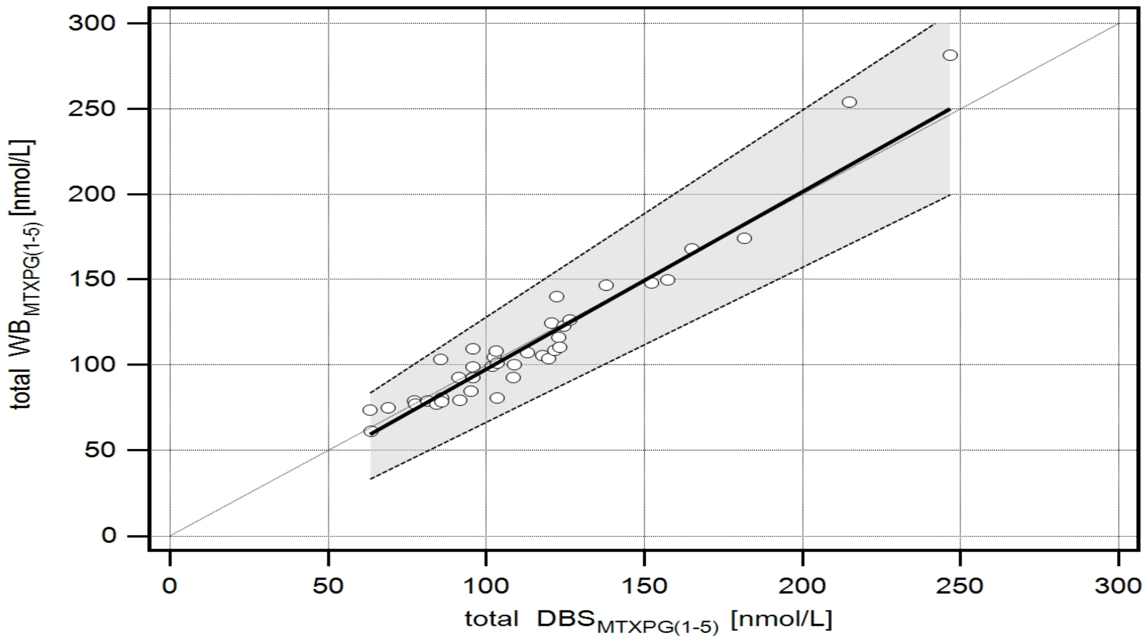

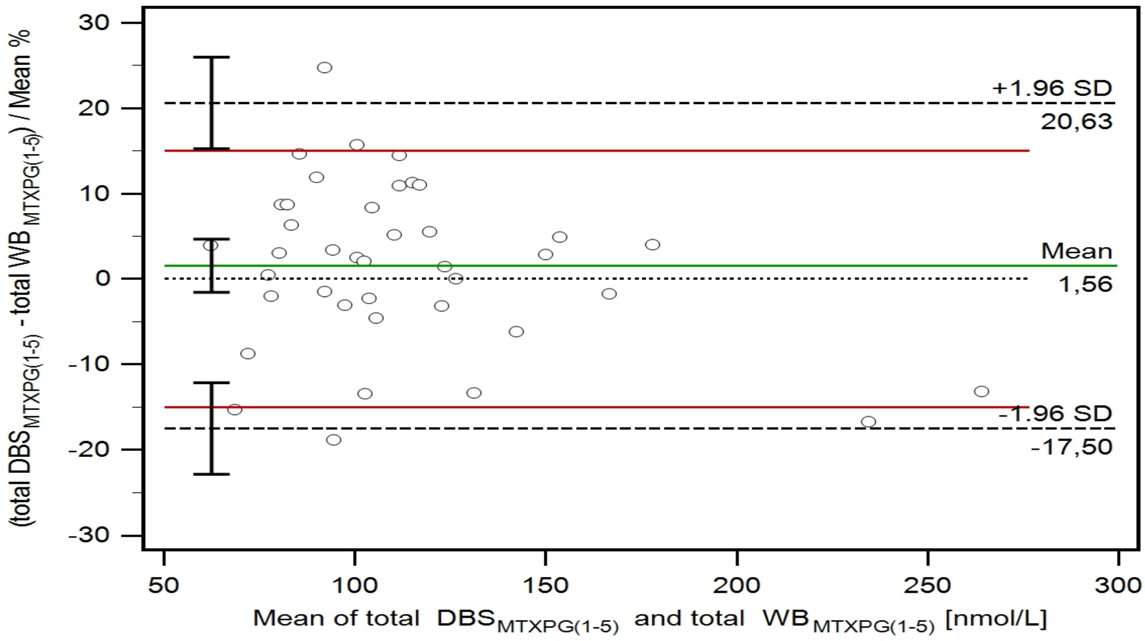

Fig. S30A–B. Total MTXPG (1–5): PB regression (A) and BA analysis (B), RBC vs WB.

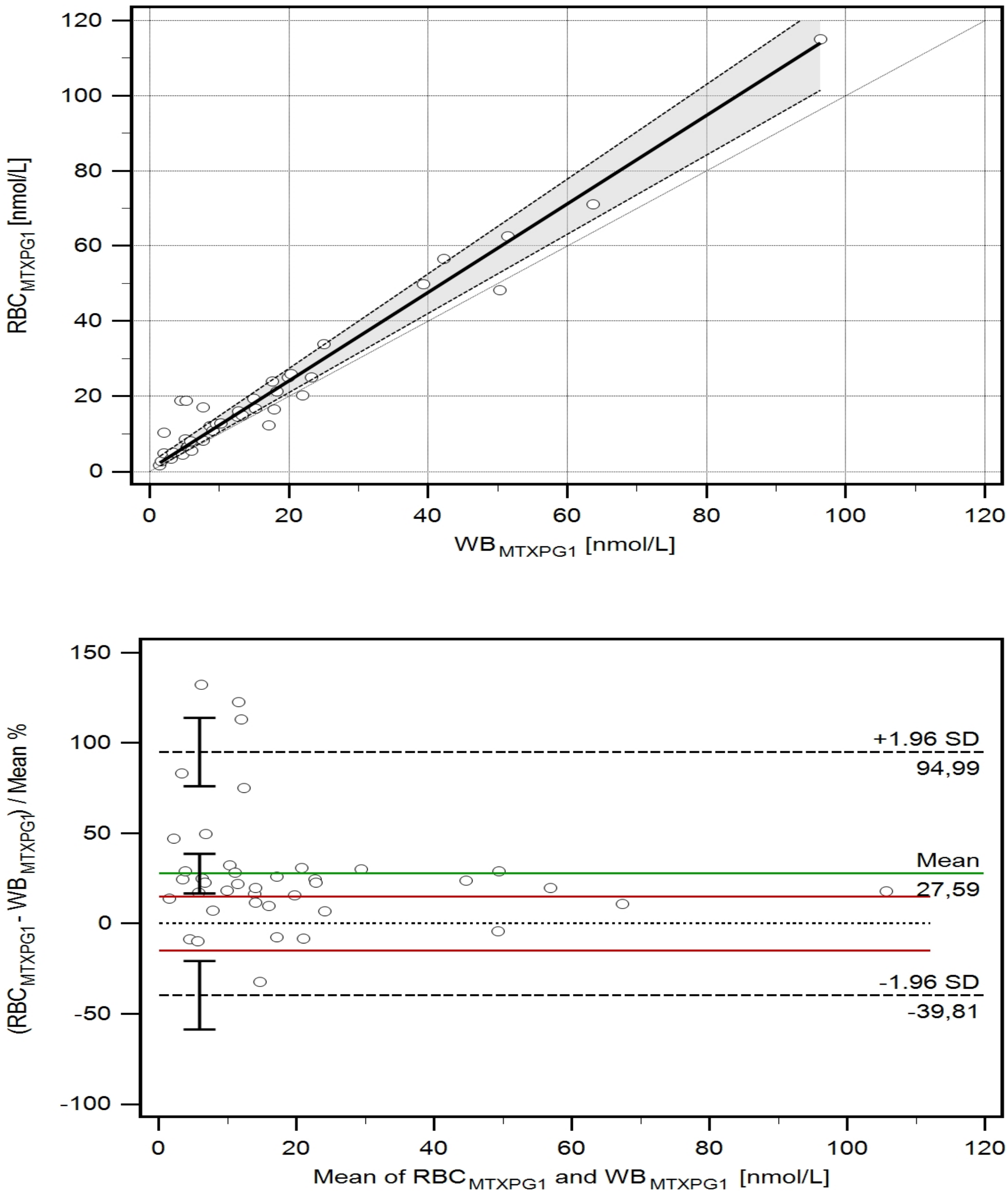

Fig. S31A–B. MTXPG1: PB regression (A) and BA analysis (B), RBC vs WB.

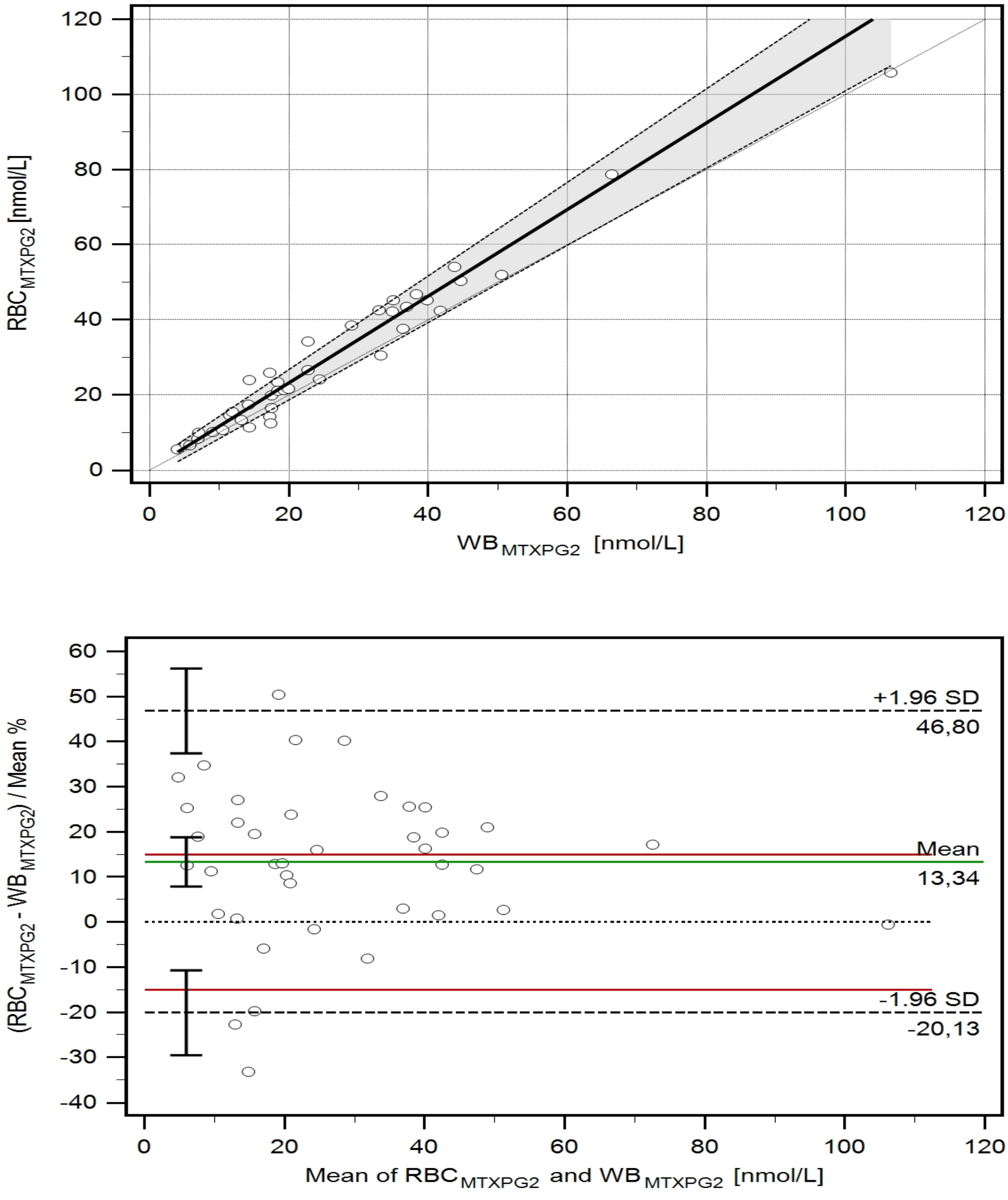

Fig. S32A–B. MTXPG2: PB regression (A) and BA analysis (B), RBC vs WB.

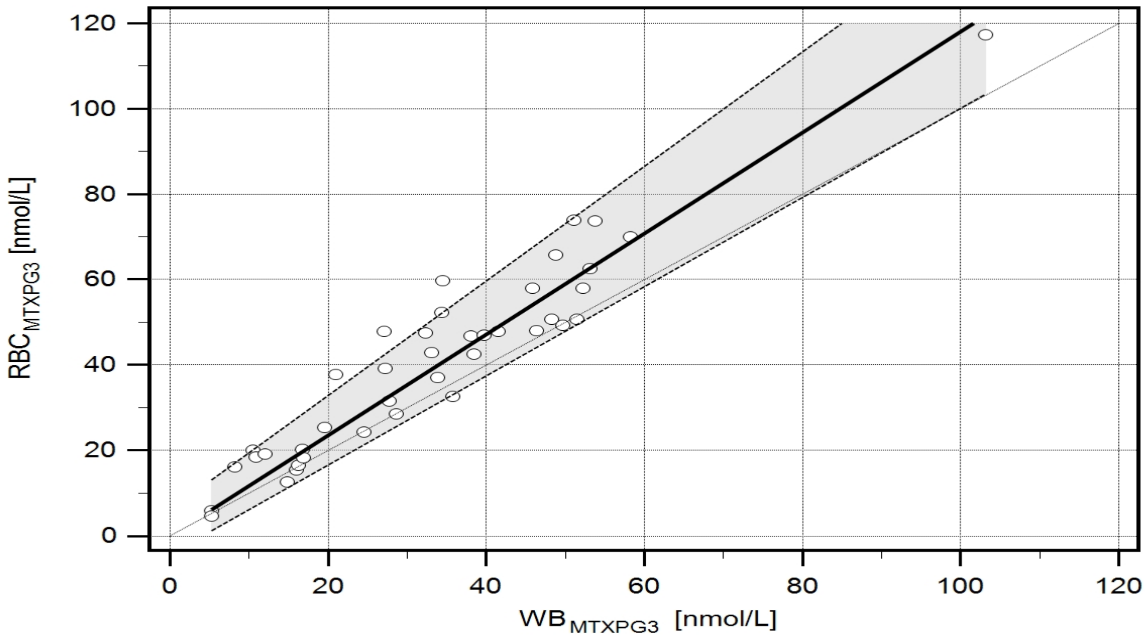

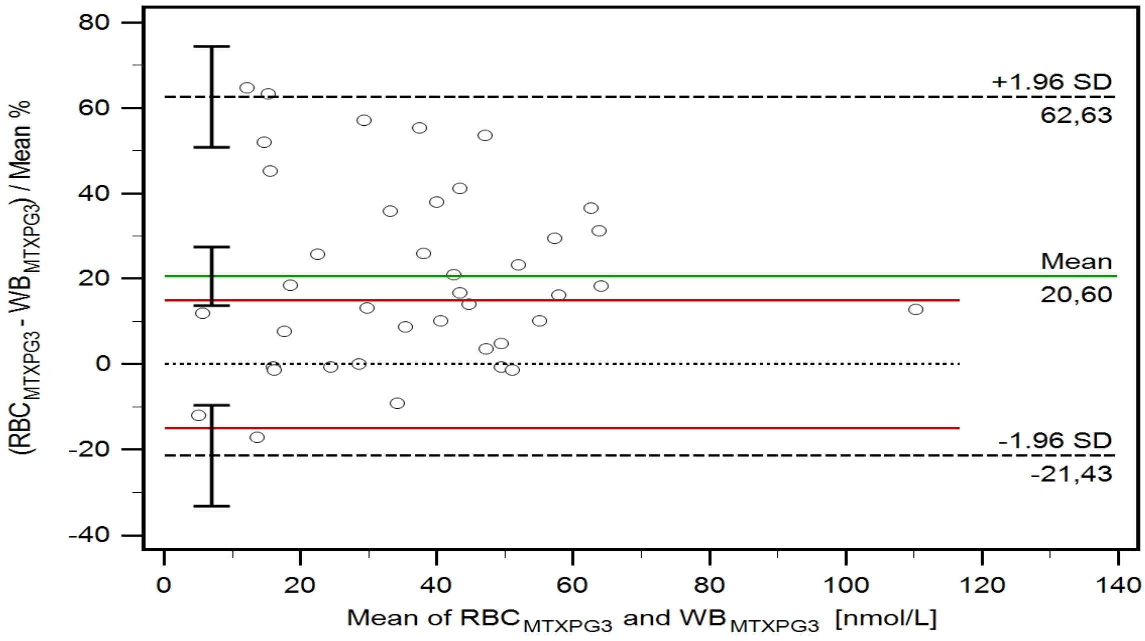

Fig. S33A–B. MTXPG3: PB regression (A) and BA analysis (B), RBC vs WB.

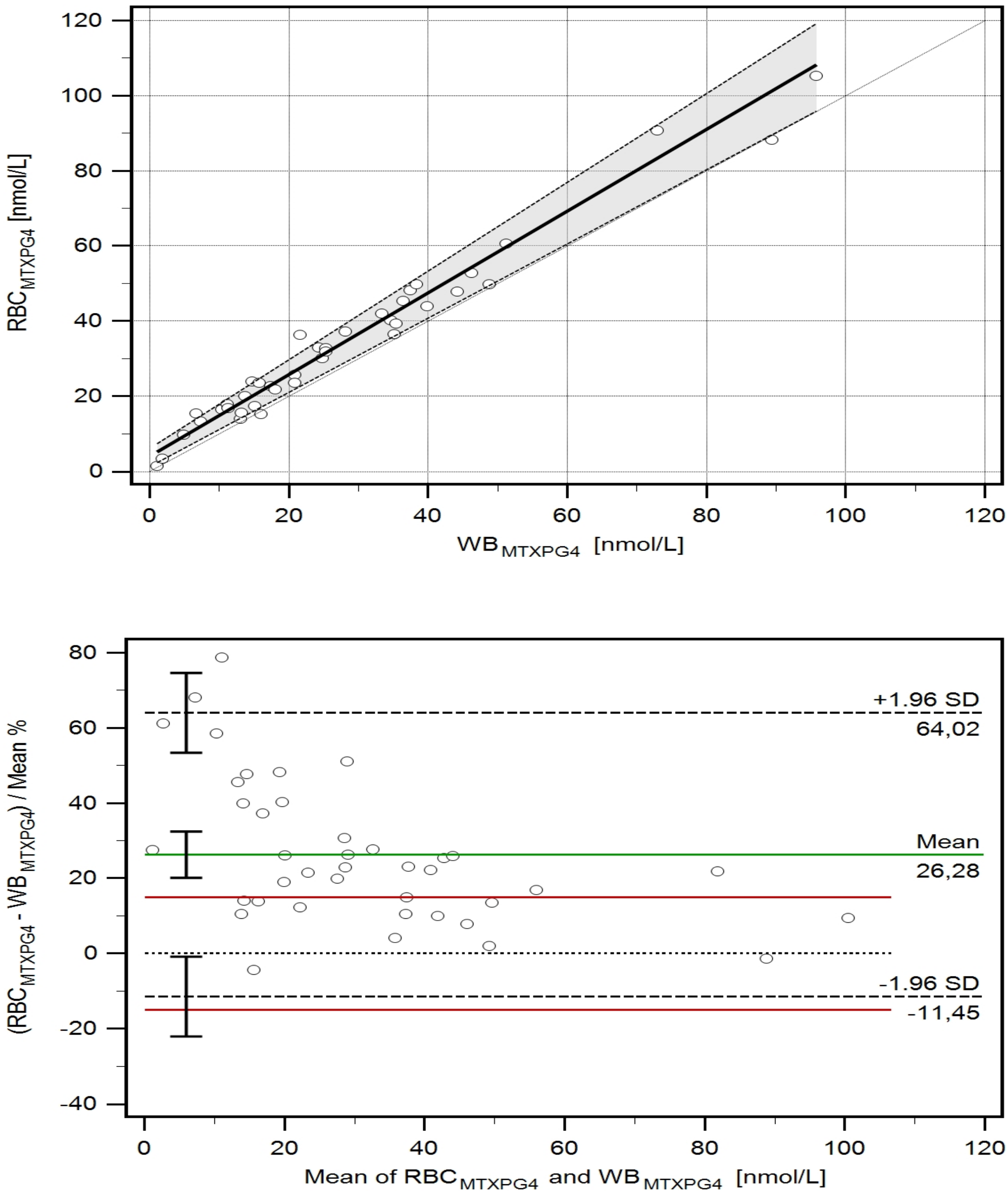

Fig. S34A–B. MTXPG4: PB regression (A) and BA analysis (B), RBC vs WB.

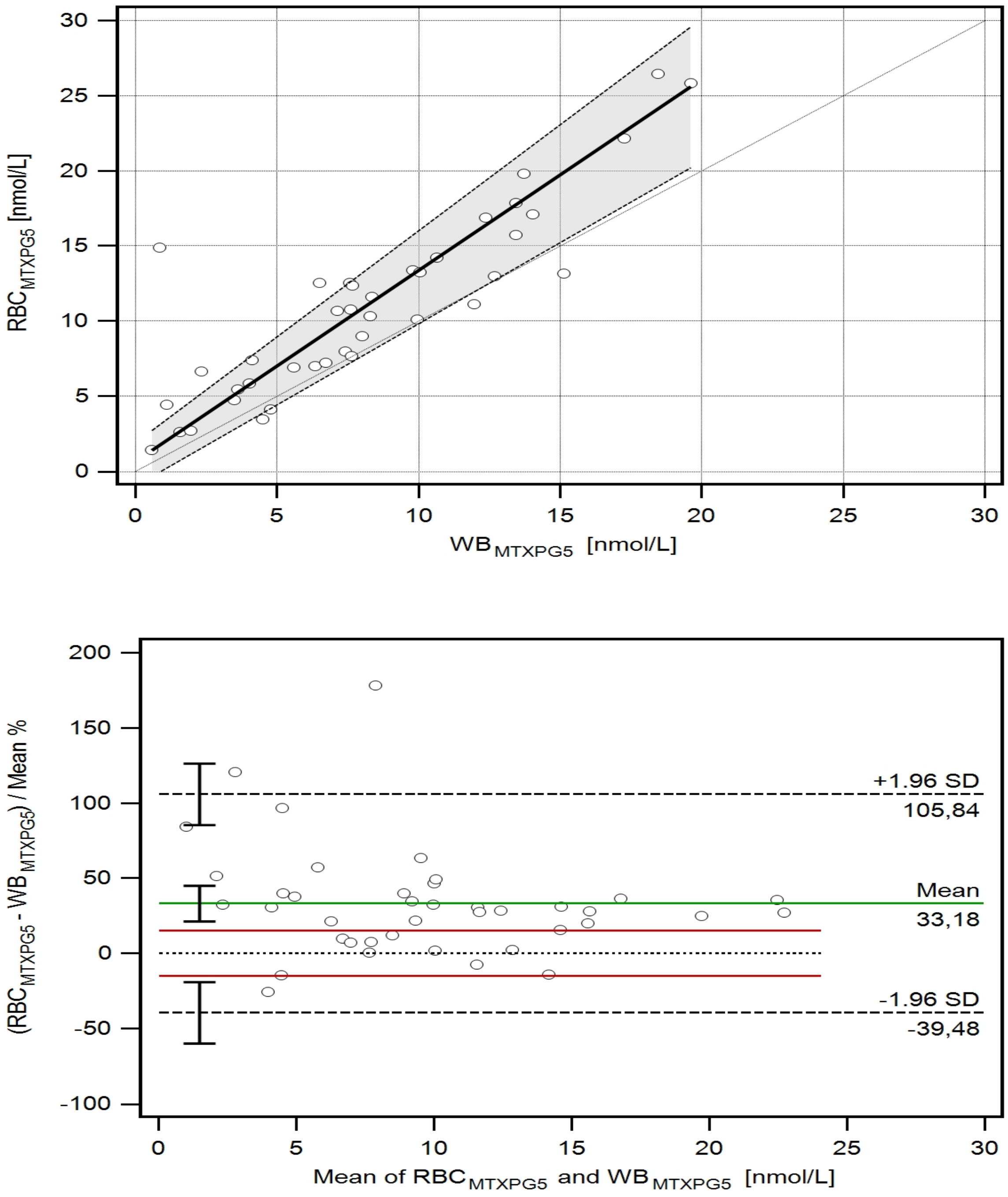

Fig. S35A–B. MTXPG5: PB regression (A) and BA analysis (B), RBC vs WB.

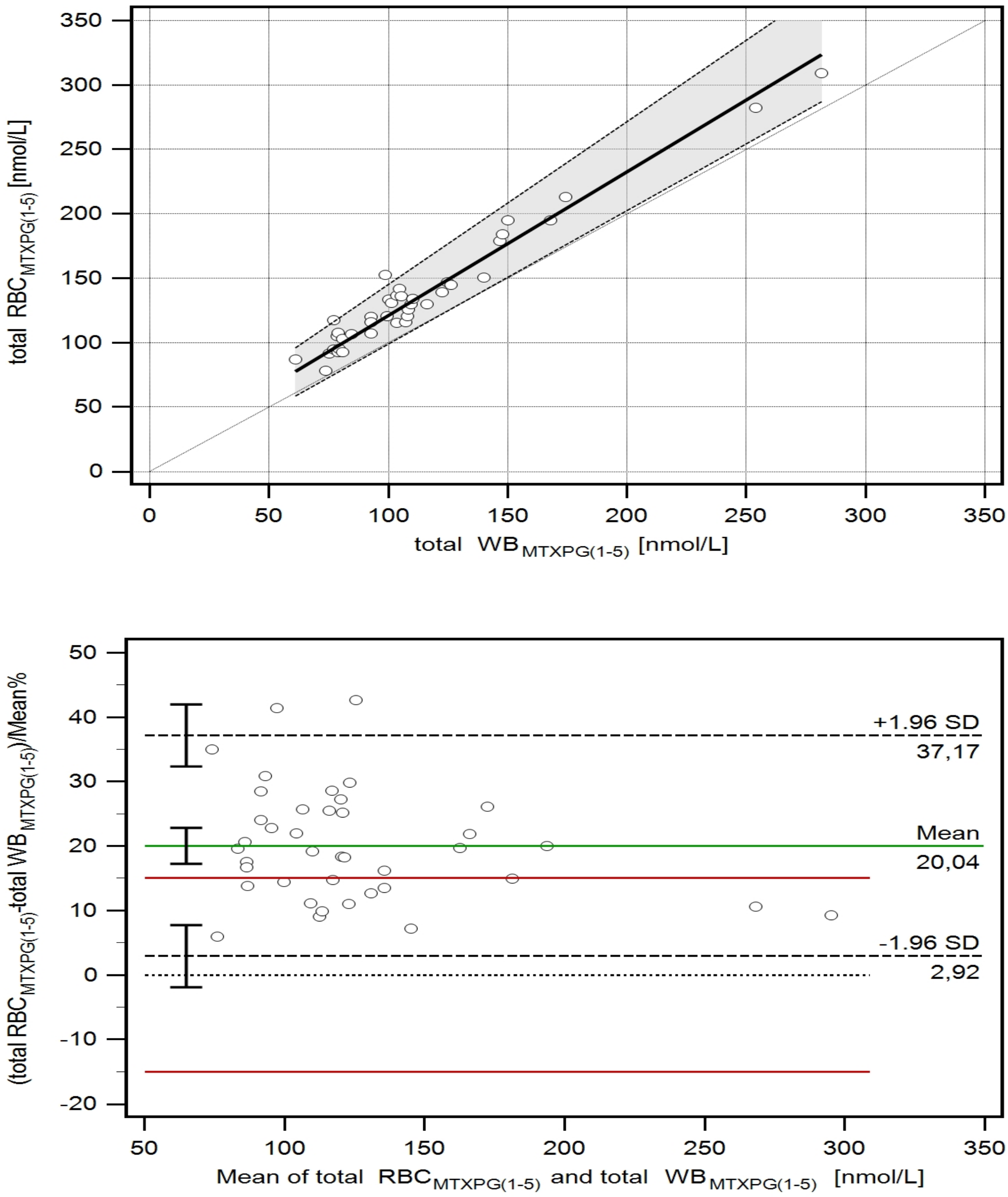

Fig. S36A–B. Total MTXPG (1–5): PB regression (A) and BA analysis (B), RBC vs WB.

Abbreviations: PB – Passing–Bablok regression; BA – Bland–Altman analysis; DBS – dried blood spots; WB – whole blood; RBC – red blood cells; MTXPG – methotrexate polyglutamates.

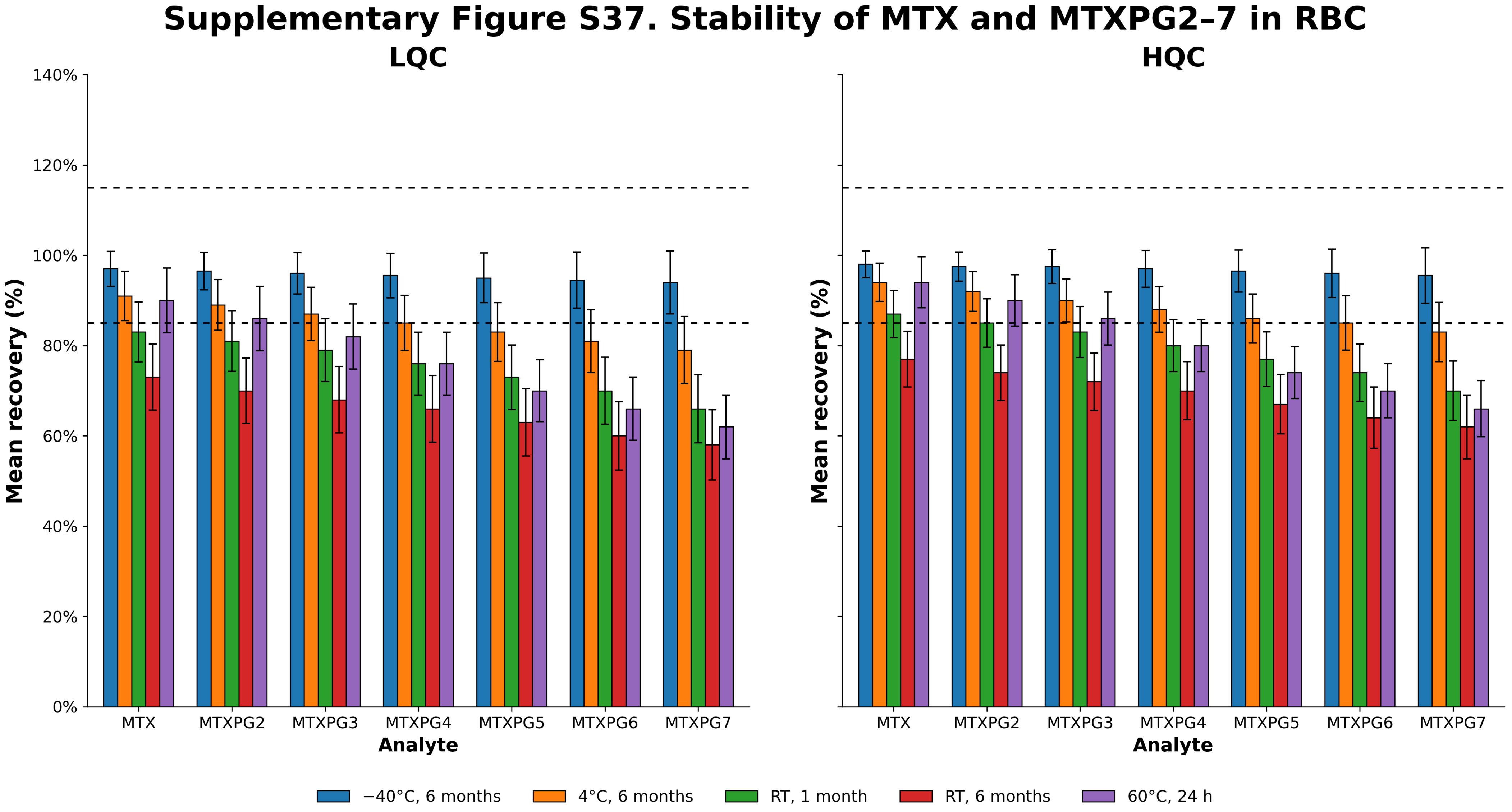

Supplementary Figure S37. Stability of MTX and MTXPG2-7 in RBC at LQC and HQC. Bars represent estimated mean recovery (%) and error bars represent CV-derived variability.

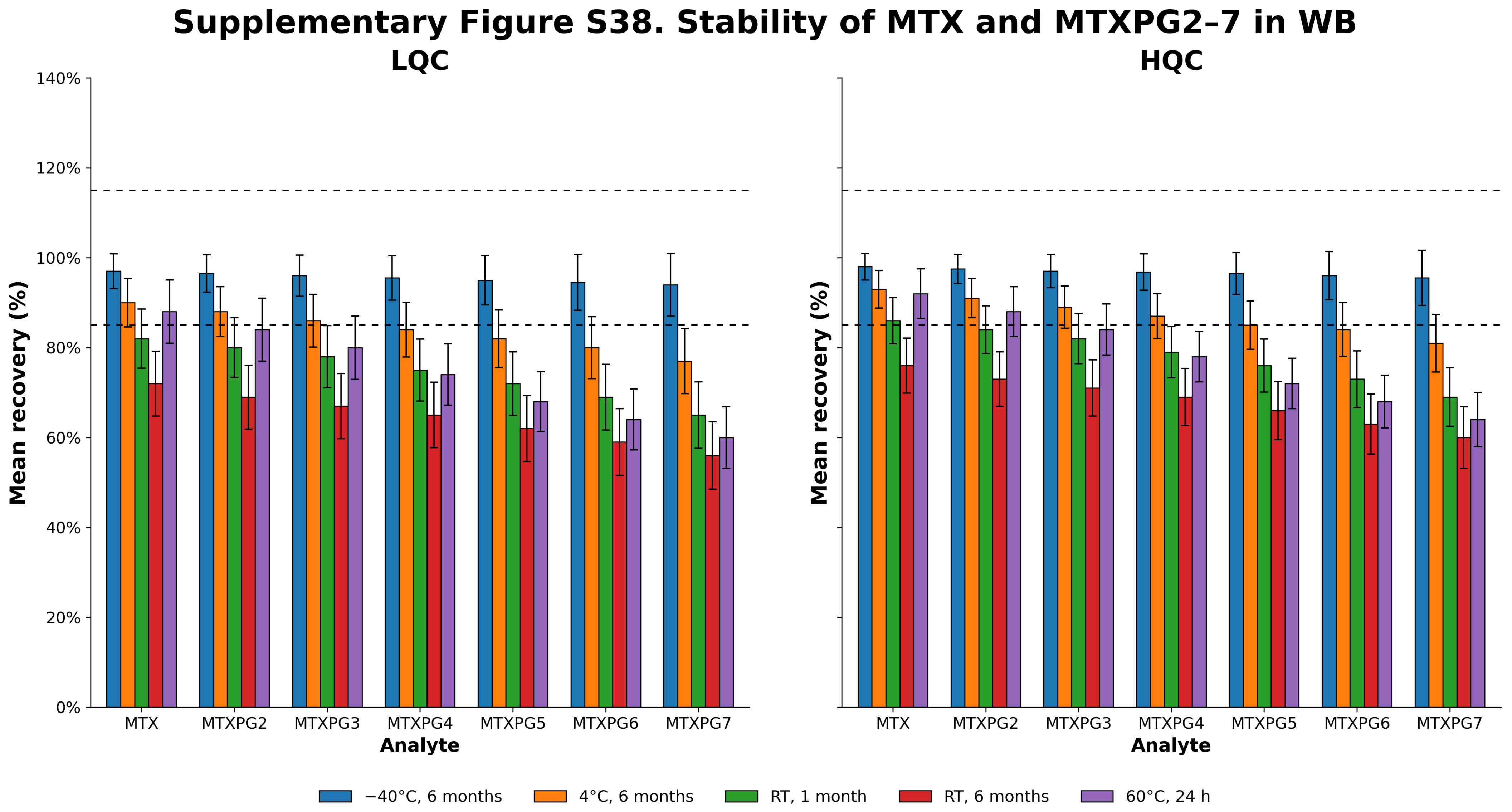

Supplementary Figure S38. Stability of MTX and MTXPG2-7 in WB at LQC and HQC. Bars represent estimated mean recovery (%) and error bars represent CV-derived variability.

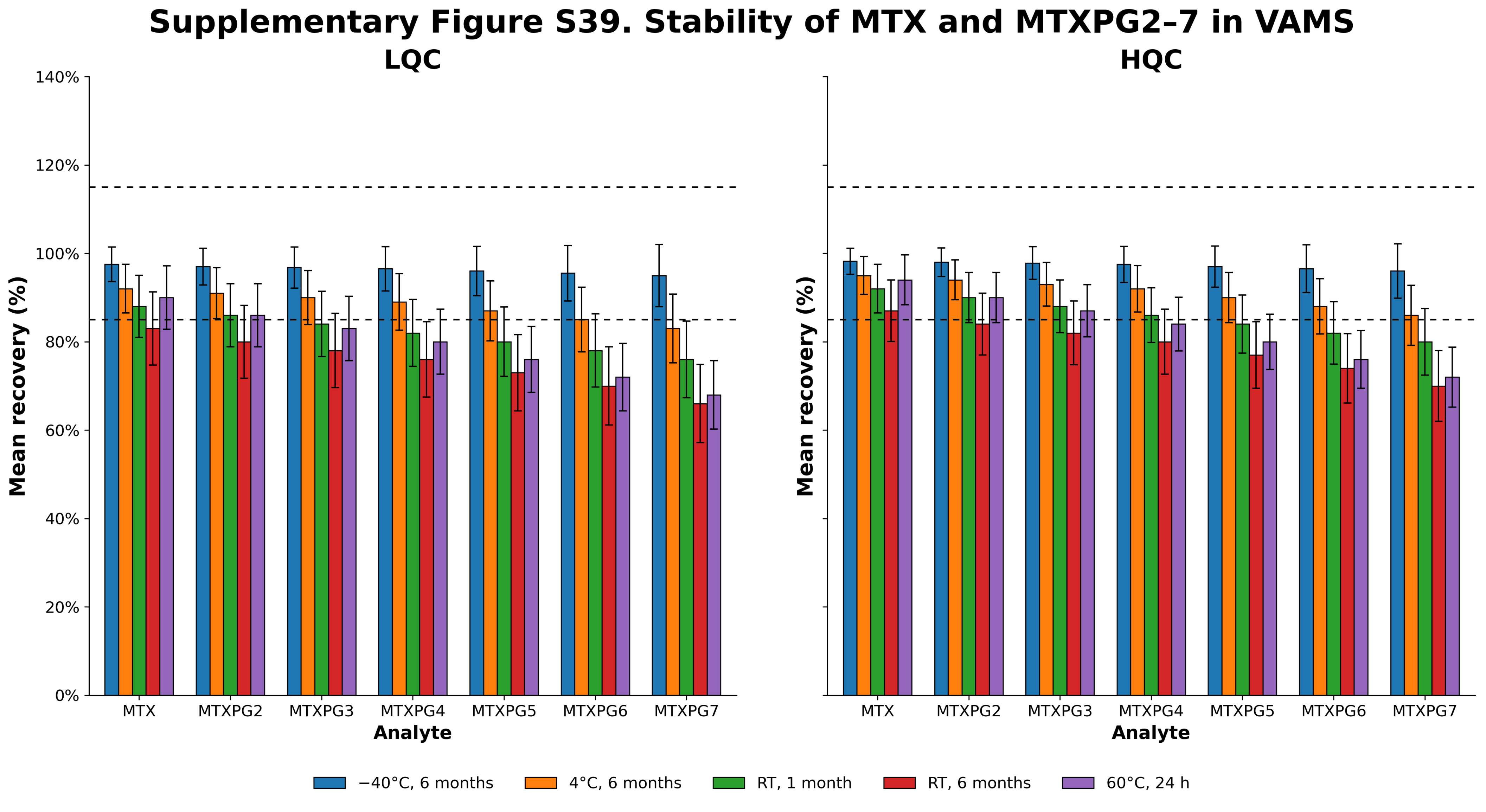

Supplementary Figure S39. Stability of MTX and MTXPG2-7 in VAMS at LQC and HQC. Bars represent estimated mean recovery (%) and error bars represent CV-derived variability.

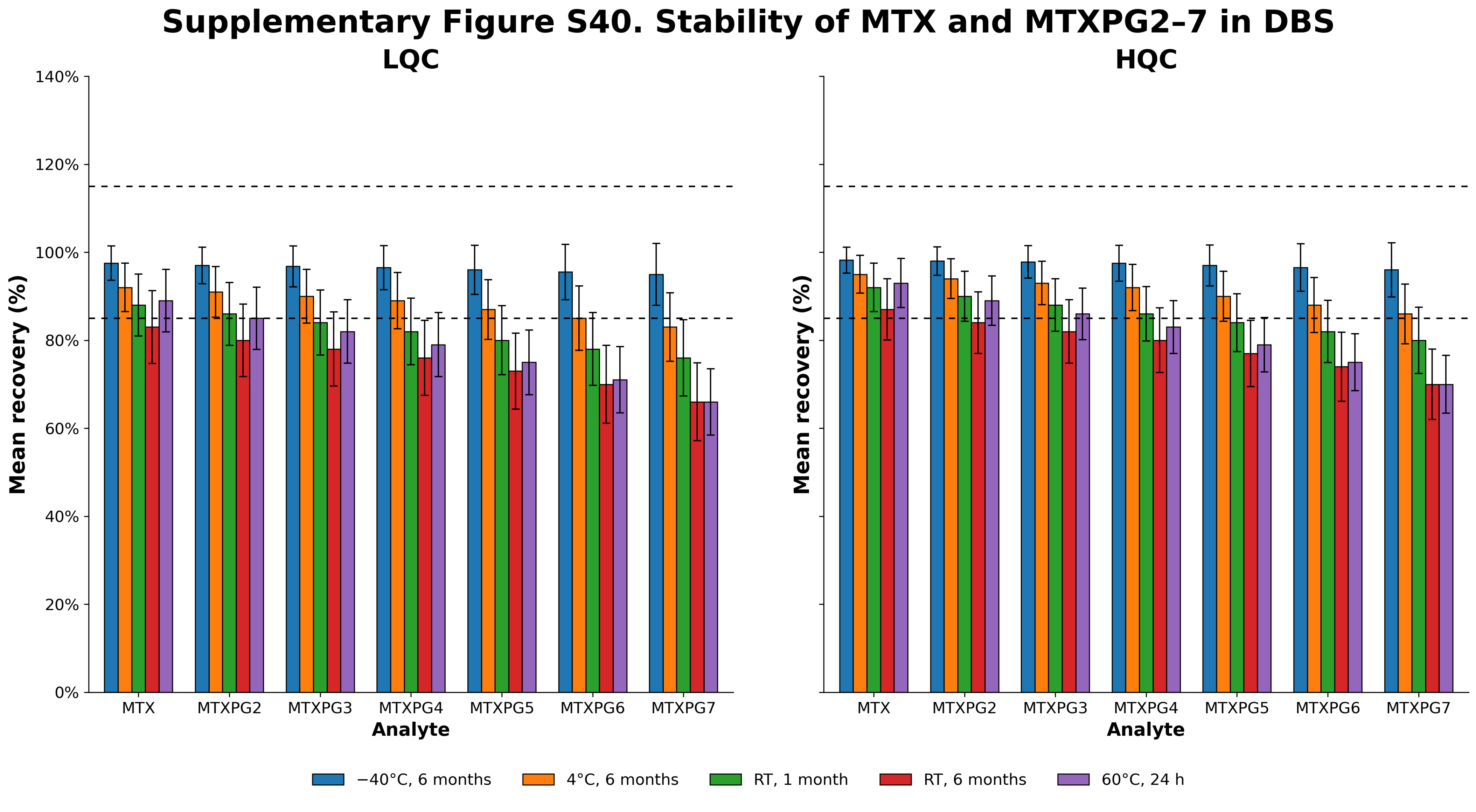

Supplementary Figure S40. Stability of MTX and MTXPG2-7 in DBS at LQC and HQC. Bars represent estimated mean recovery (%) and error bars represent CV-derived variability.

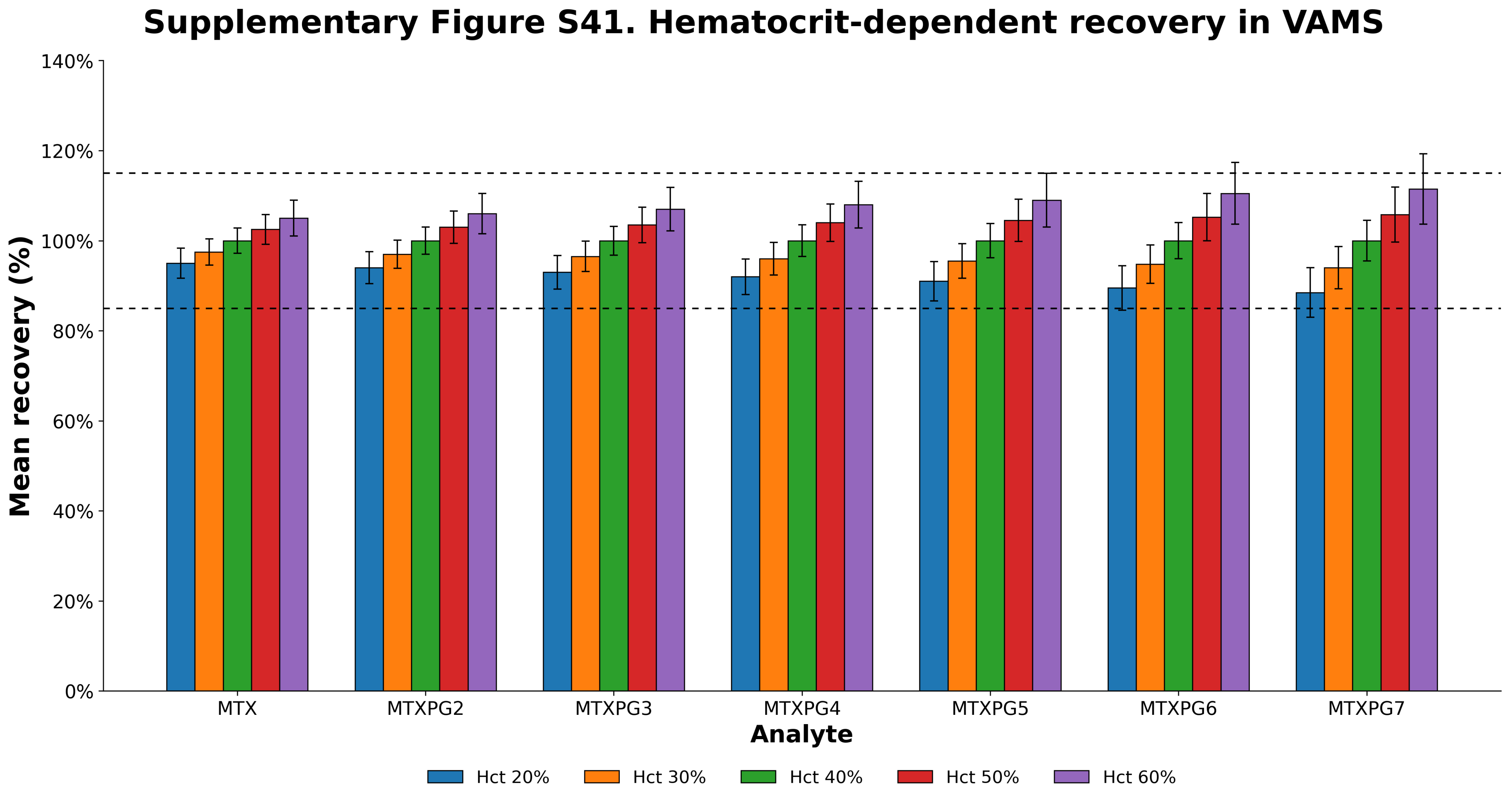

Supplementary Figure S41. Hematocrit-dependent recovery of MTX and MTXPG2-7 in VAMS. Bars represent estimated mean recovery (%) at each hematocrit level and error bars represent CV-derived variability.

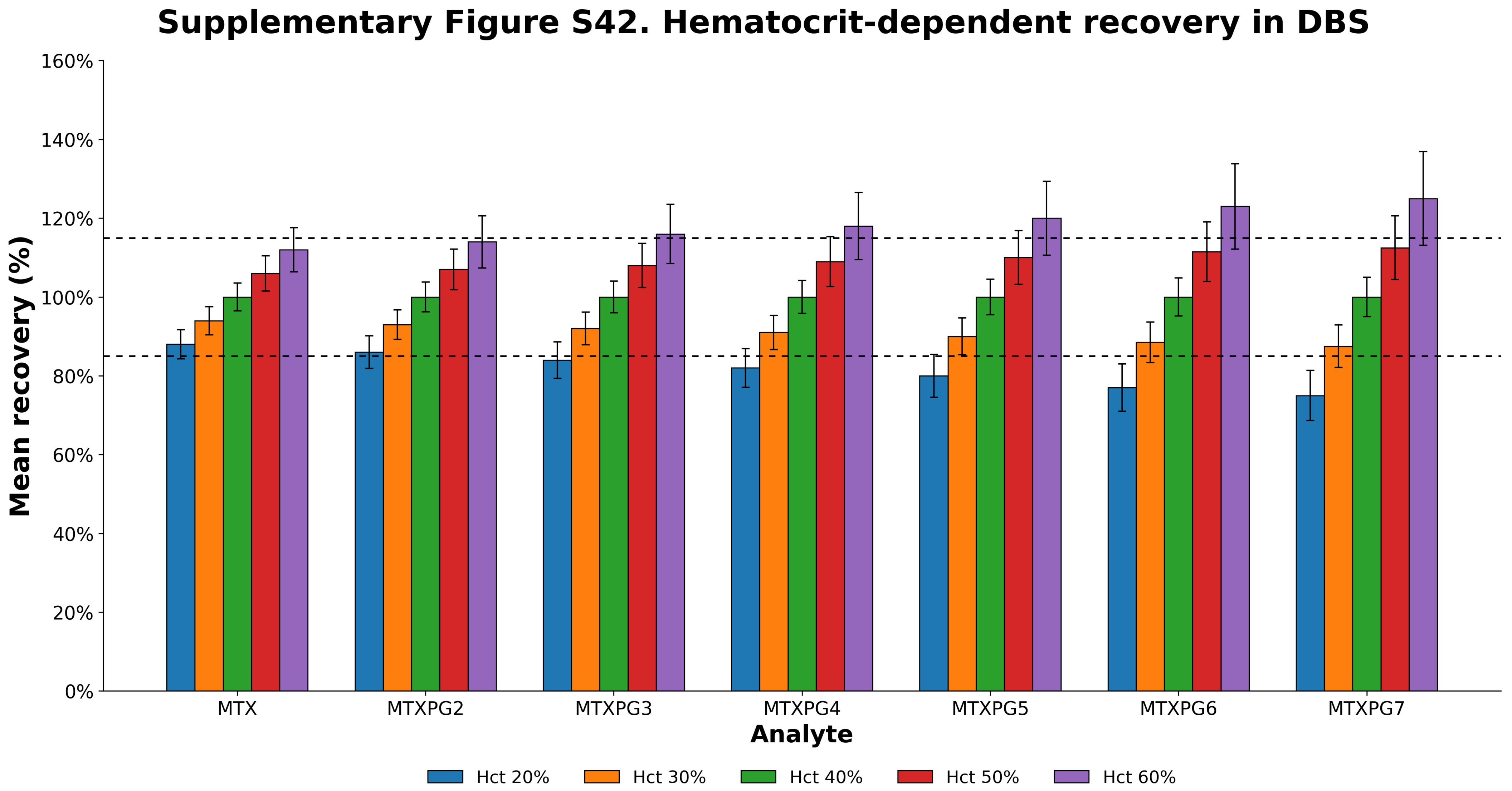

Supplementary Figure S42. Hematocrit-dependent recovery of MTX and MTXPG2-7 in DBS. Bars represent estimated mean recovery (%) at each hematocrit level and error bars represent CV-derived variability.
